# Supplementary material for: Transferability of ISSR, SCoT and SSR Markers for Chrysanthemum × Morifolium Ramat and Genetic Relationships Among Commercial Russian Cultivars
Source: Plants (Basel). 2021 Jun 27;10(7):1302. doi: 10.3390/plants10071302 (PMC8309030; doi:10.3390/plants10071302)
Supplement: Supplementary file 1 [file plants-10-01302-s001.zip › All gel pictures.pdf]

Electrophoresis images obtained by SCoT, ISSR and SSR amplification on chrysanthemum

SCoT20_1-16

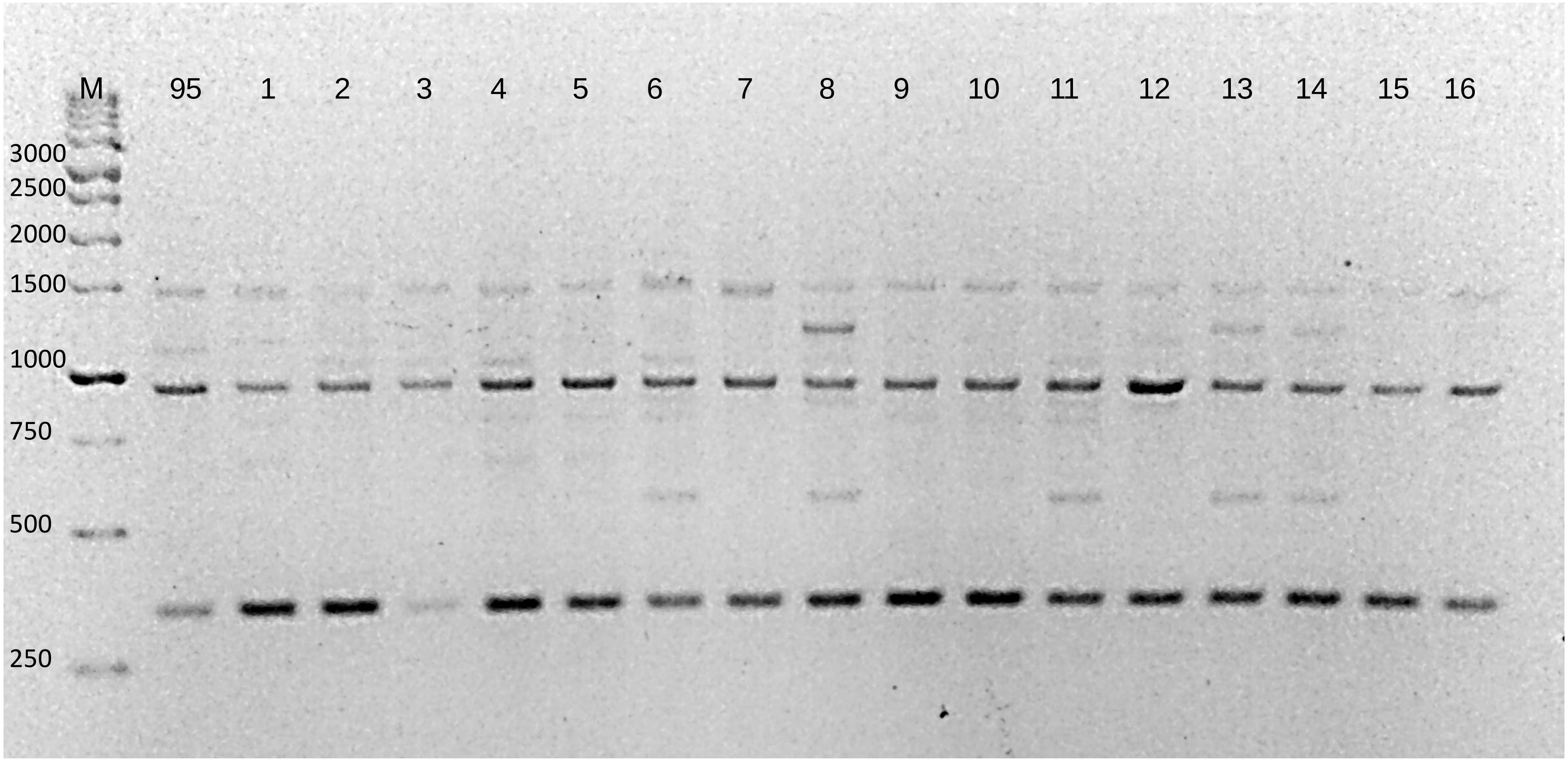
M
95
1
2
3
4
5
6
7
8
9
10
11
12
13
14
15
16
3000
2500
2000
1500
1000
750
500
250

SCoT20_17-32

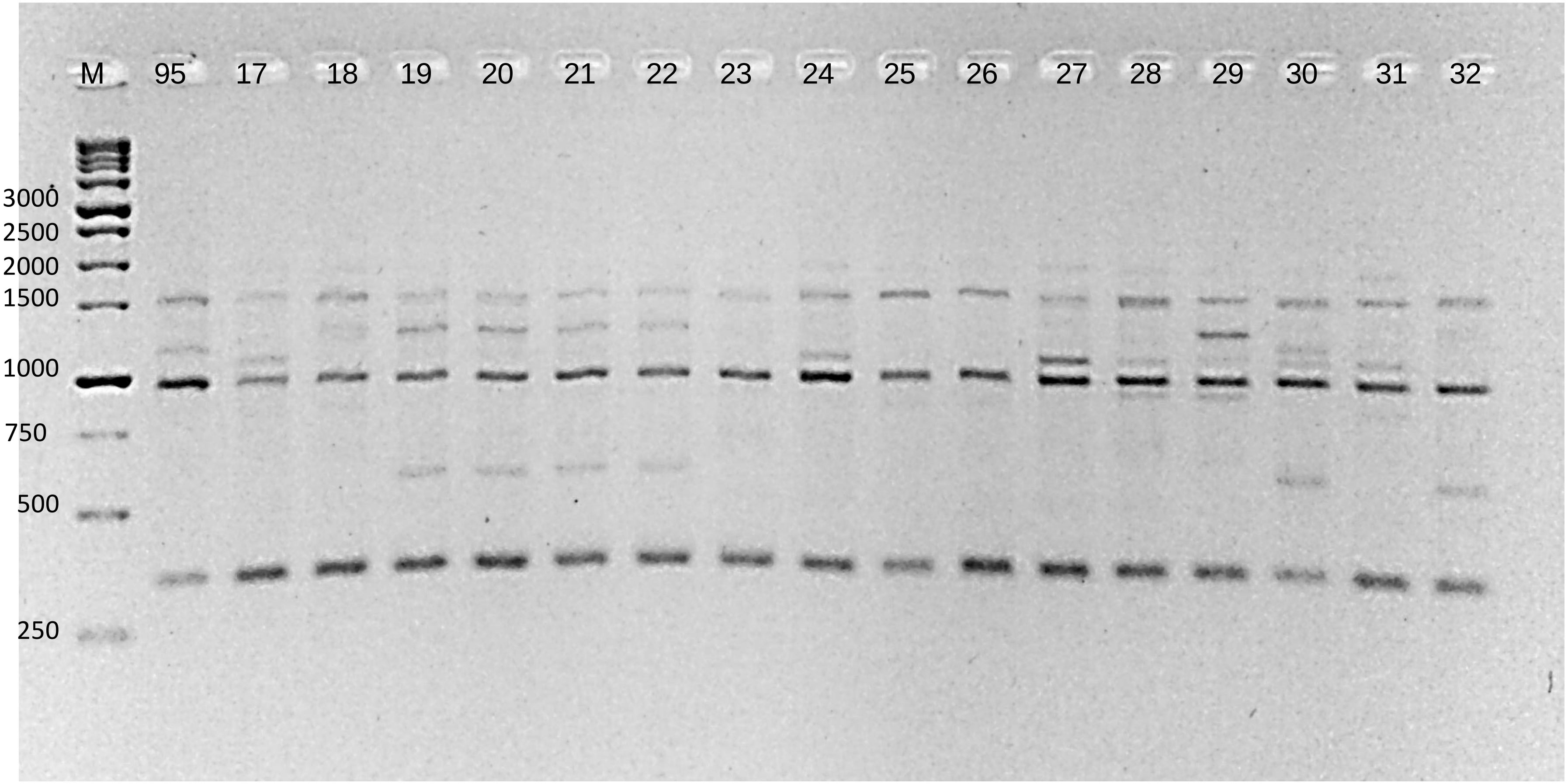

M
95
17
18
19
20
21
22
23
24
25
26
27
28
29
30
31
32
3000
2500
2000
1500
1000
750
500
250

SCoT20_33-48

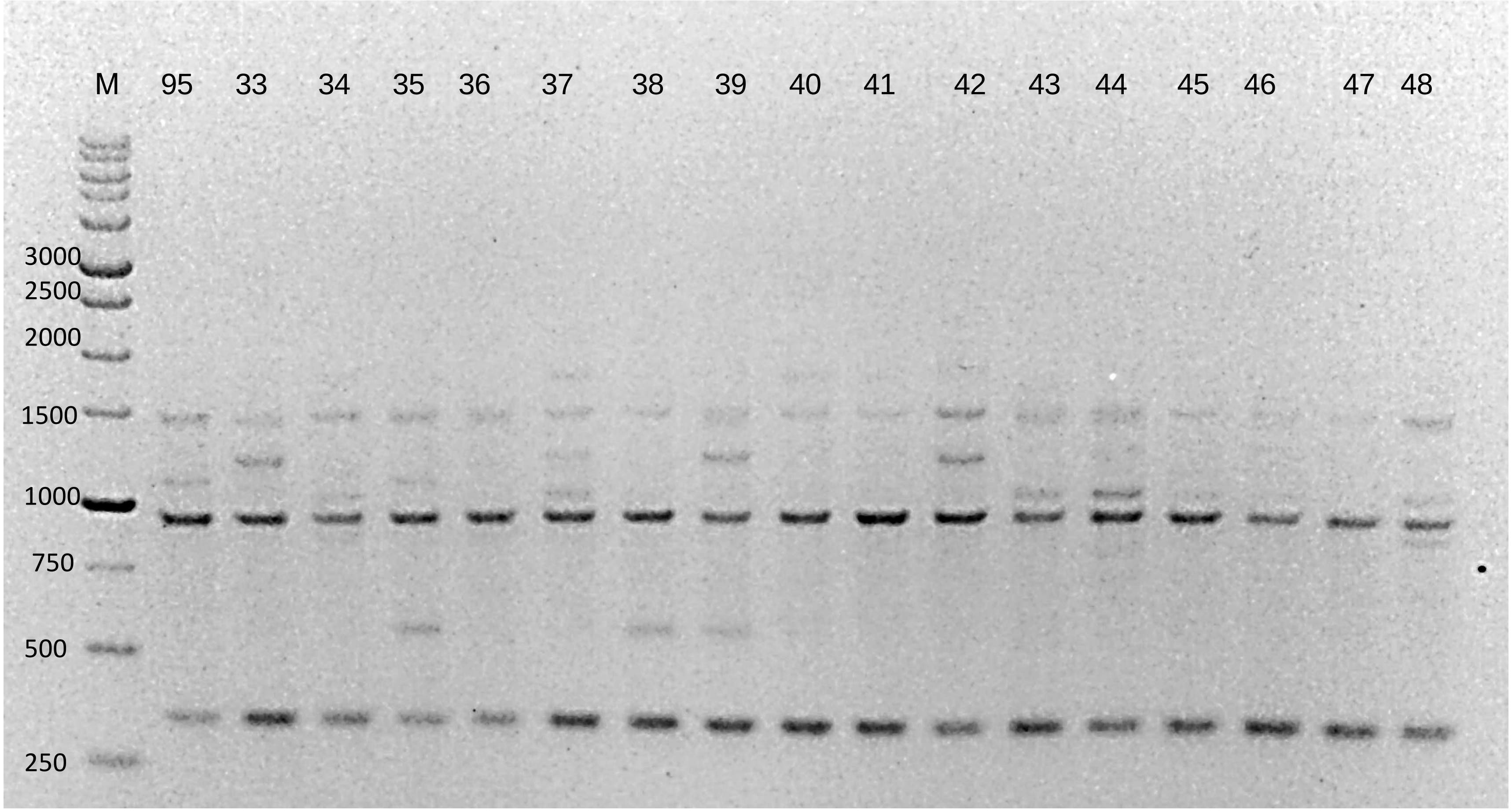

M
95
33
34
35
36
37
38
39
40
41
42
43
44
45
46
47
48
3000
2500
2000
1500
1000
750
500
250

SCoT20_49-64

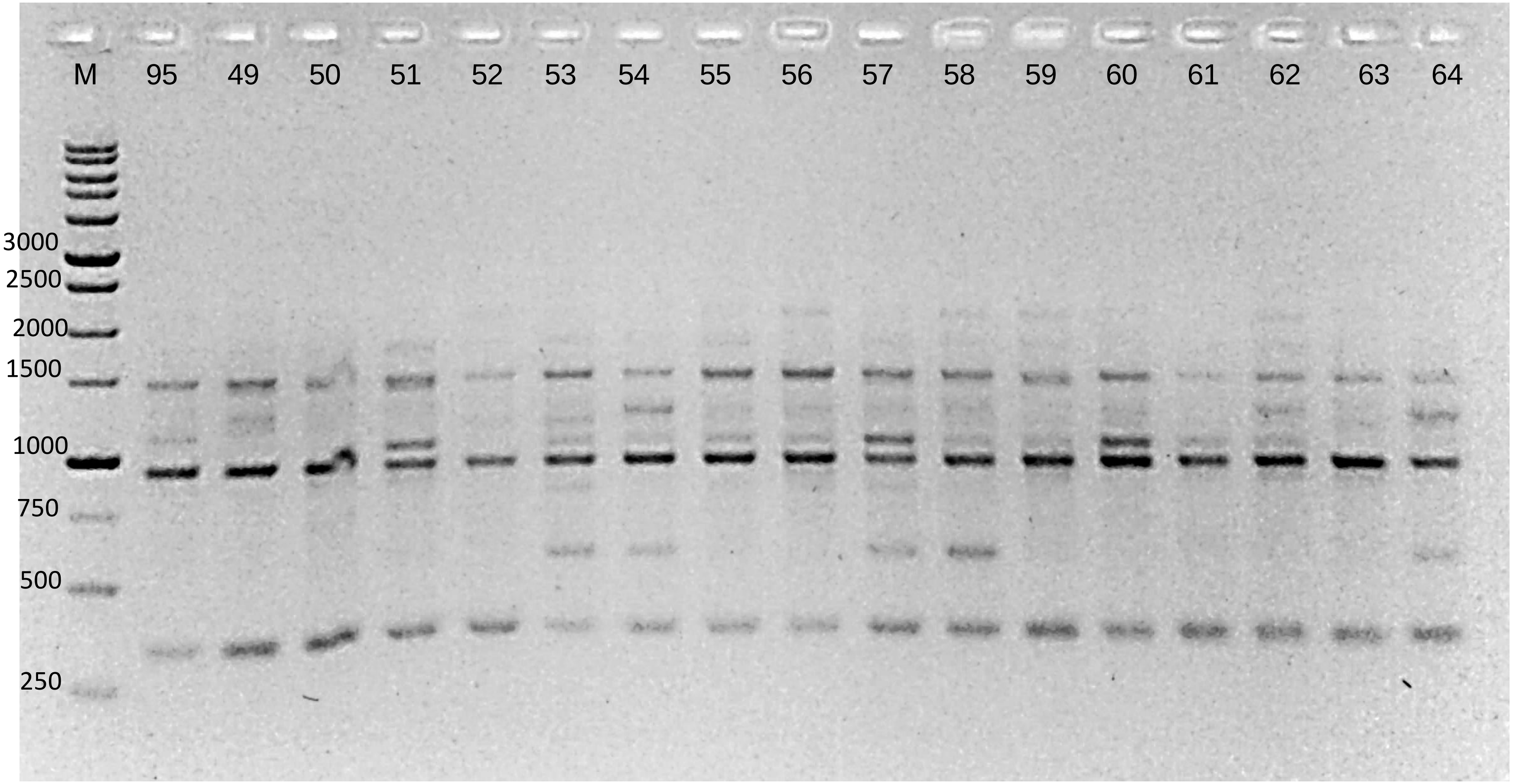
M
95
49
50
51
52
53
54
55
56
57
58
59
60
61
62
63
64
3000
2500
2000
1500
1000
750
500
250

SCoT20_65-81

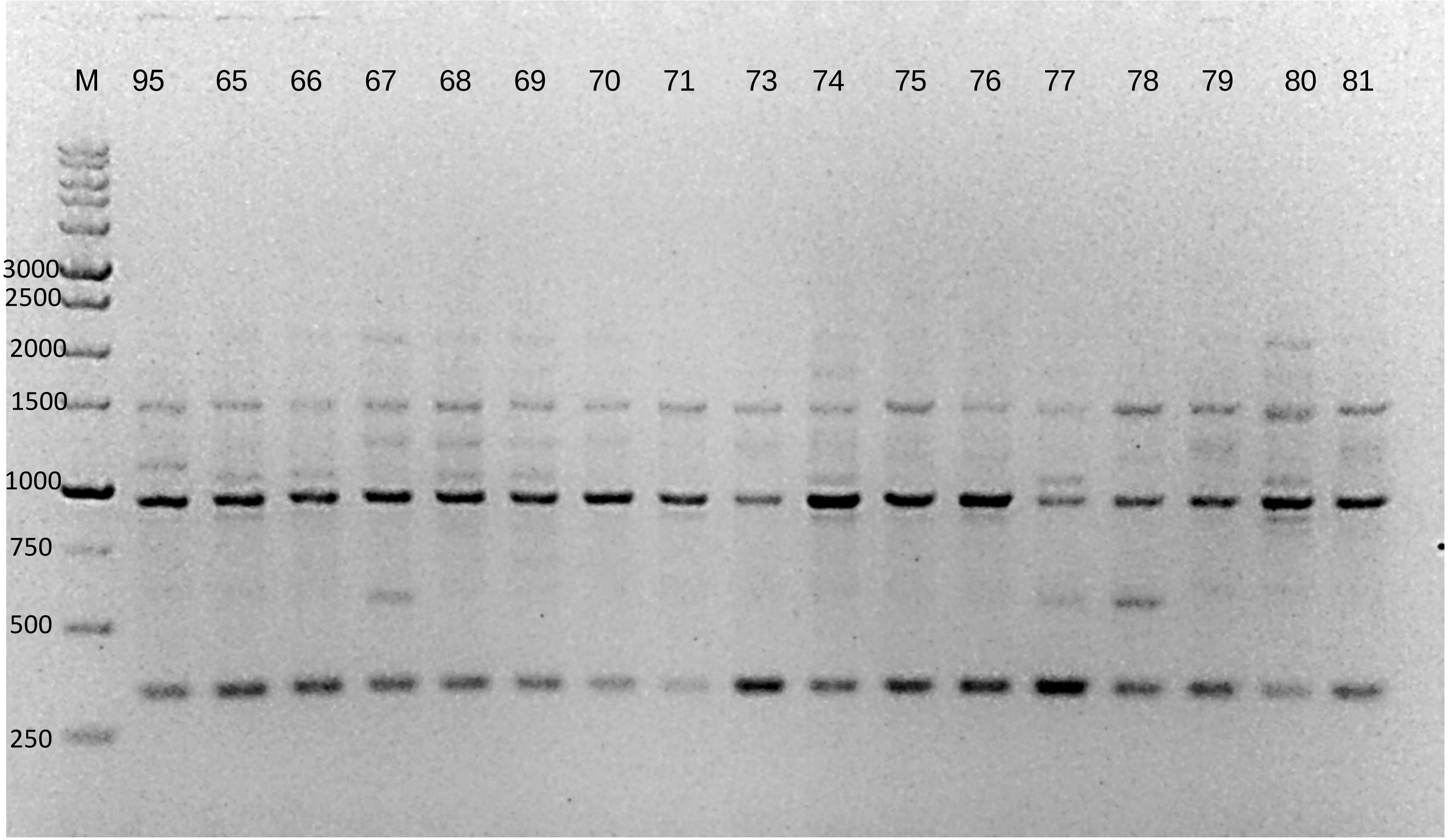
M
95
65
66
67
68
69
70
71
73
74
75
76
77
78
79
80
81
3000
2500
2000
1500
1000
750
500
250

SCoT20_82-94

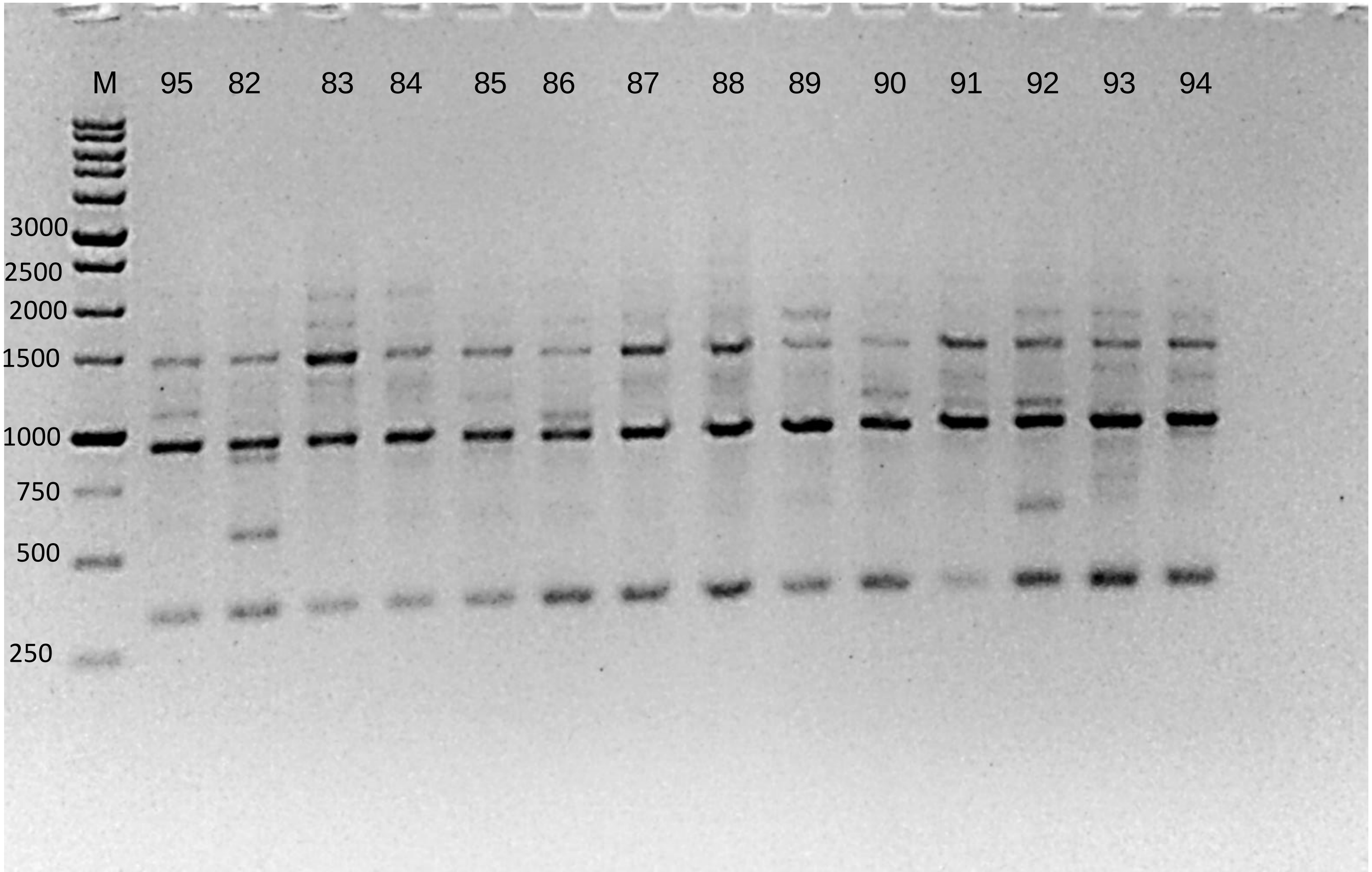
M
95
82
83
84
85
86
87
88
89
90
91
92
93
94
3000
2500
2000
1500
1000
750
500
250

SCoT23_01-16

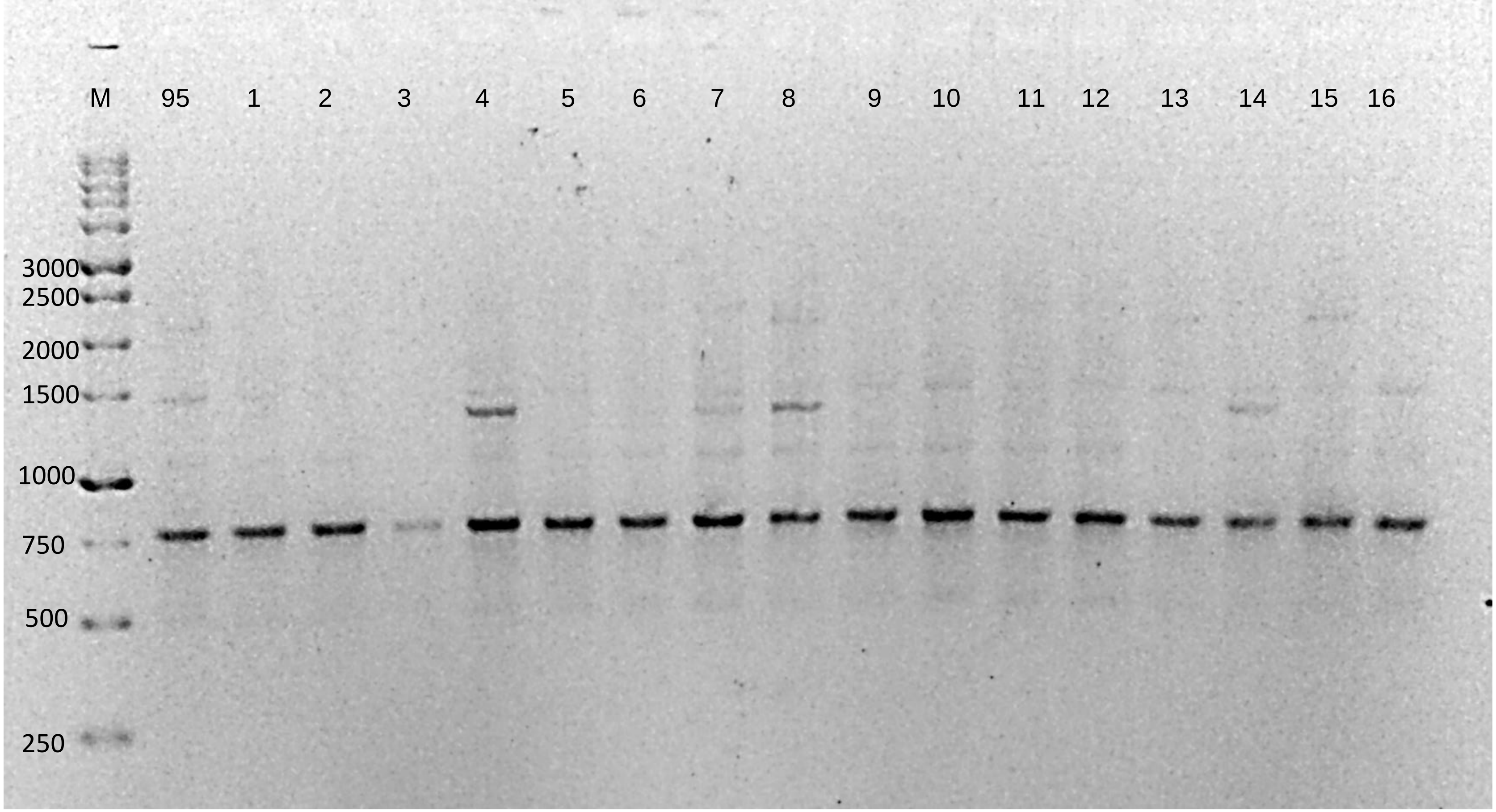
M
95
1
2
3
4
5
6
7
8
9
10
11
12
13
14
15
16
3000
2500
2000
1500
1000
750
500
250

SCoT23_17-32

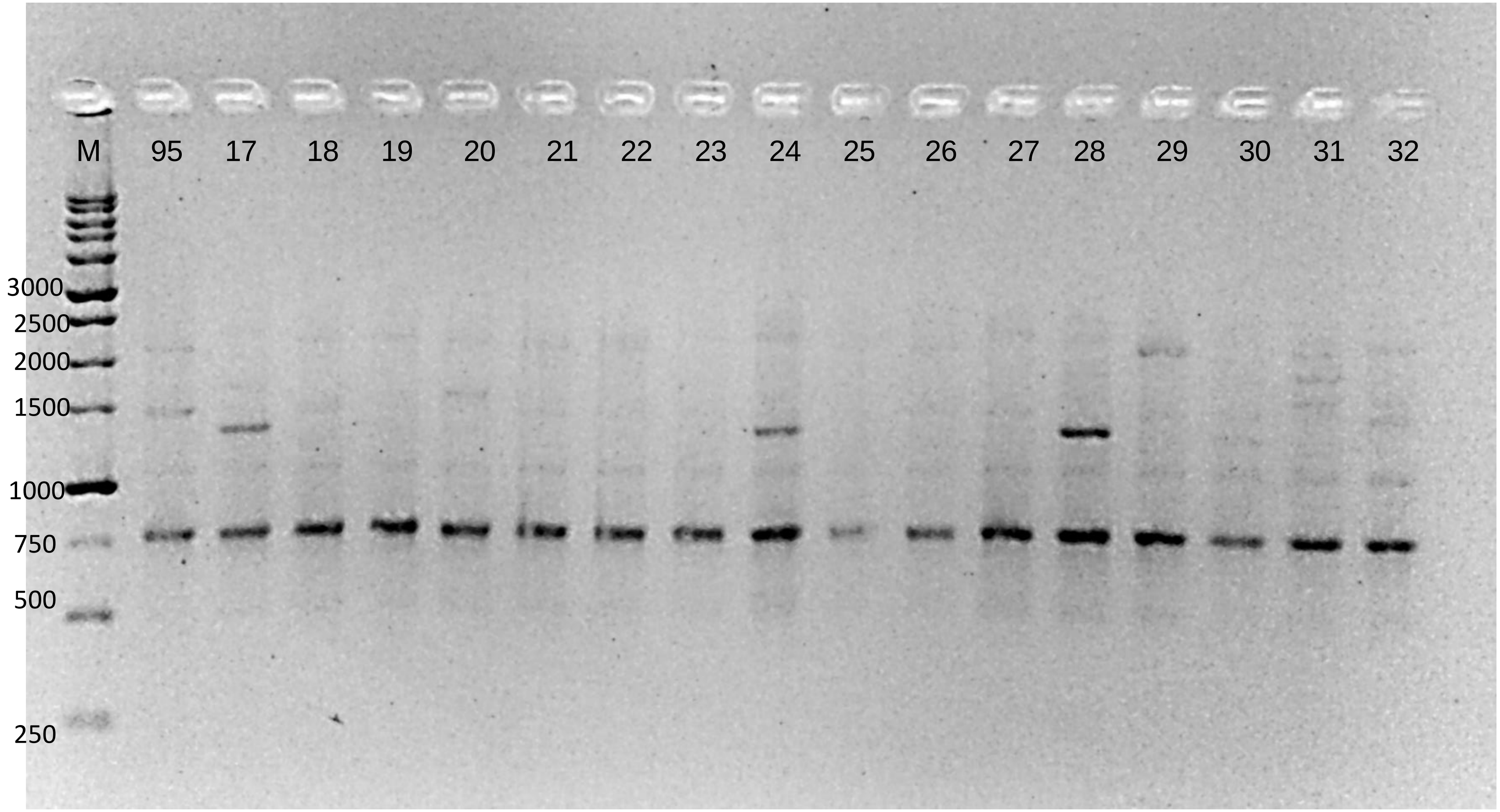

M
95
17
18
19
20
21
22
23
24
25
26
27
28
29
30
31
32
3000
2500
2000
1500
1000
750
500
250

SCoT23_33-48

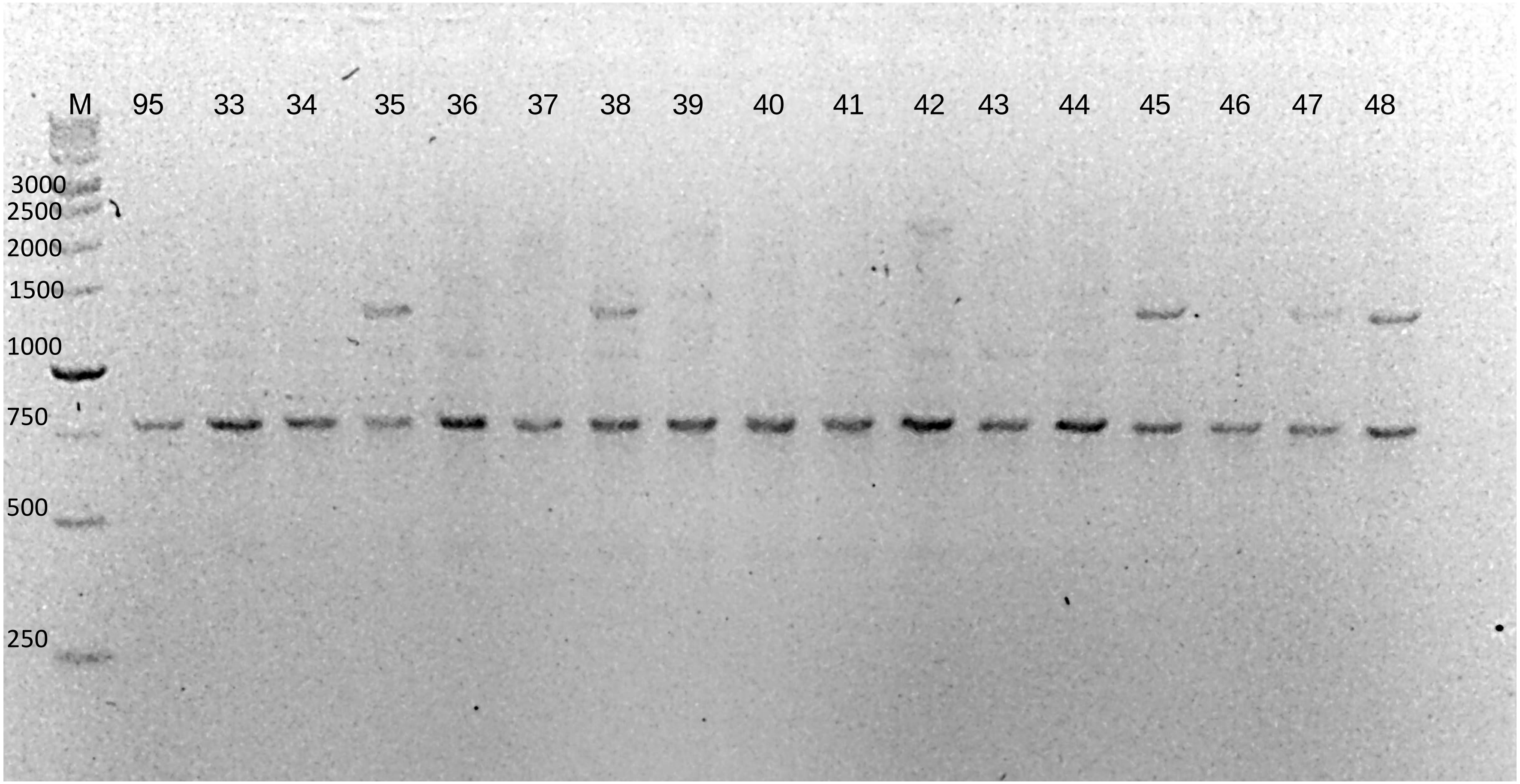

M
95
33
34
35
36
37
38
39
40
41
42
43
44
45
46
47
48
3000
2500
2000
1500
1000
750
500
250

SCoT23_49-64

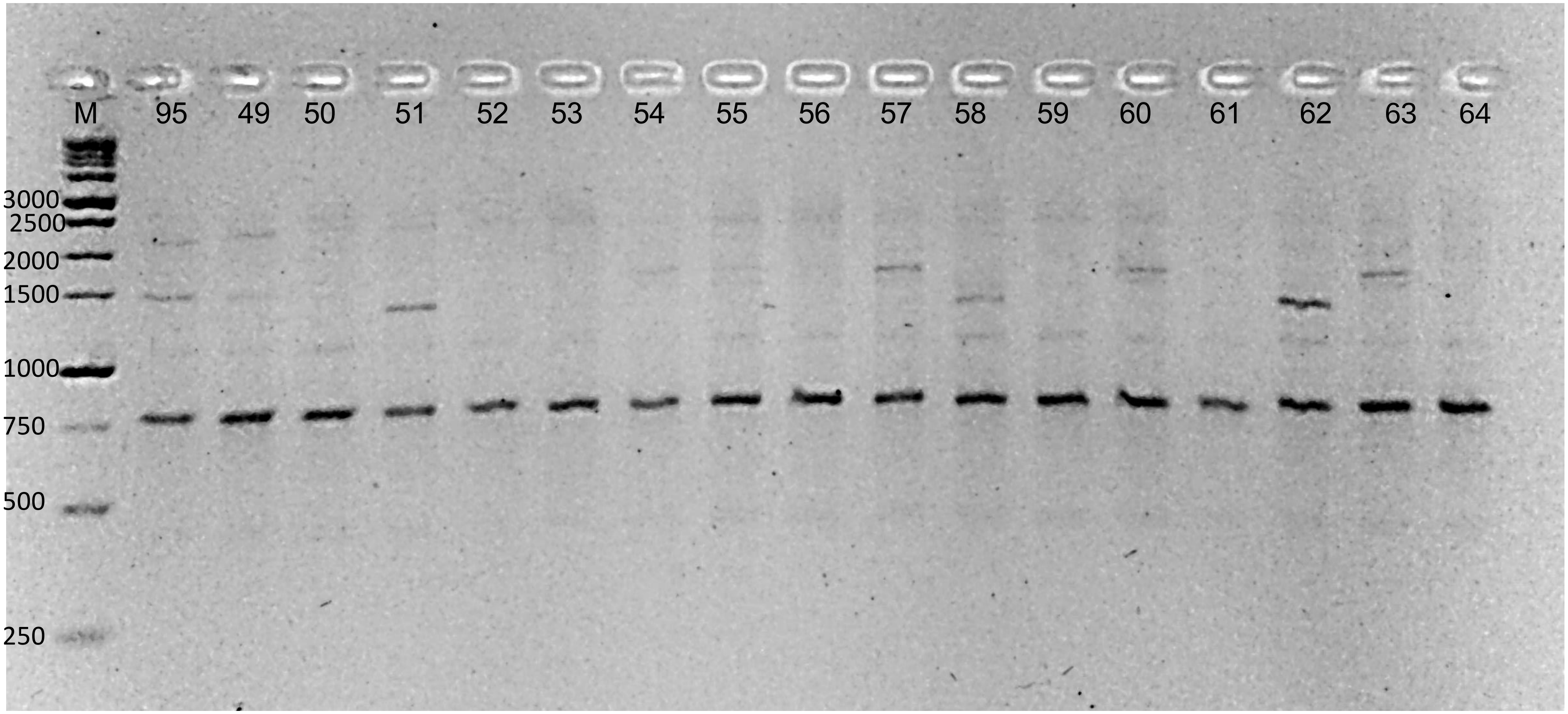
M
95
49
50
51
52
53
54
55
56
57
58
59
60
61
62
63
64
3000
2500
2000
1500
1000
750
500
250

SCoT23_65-81

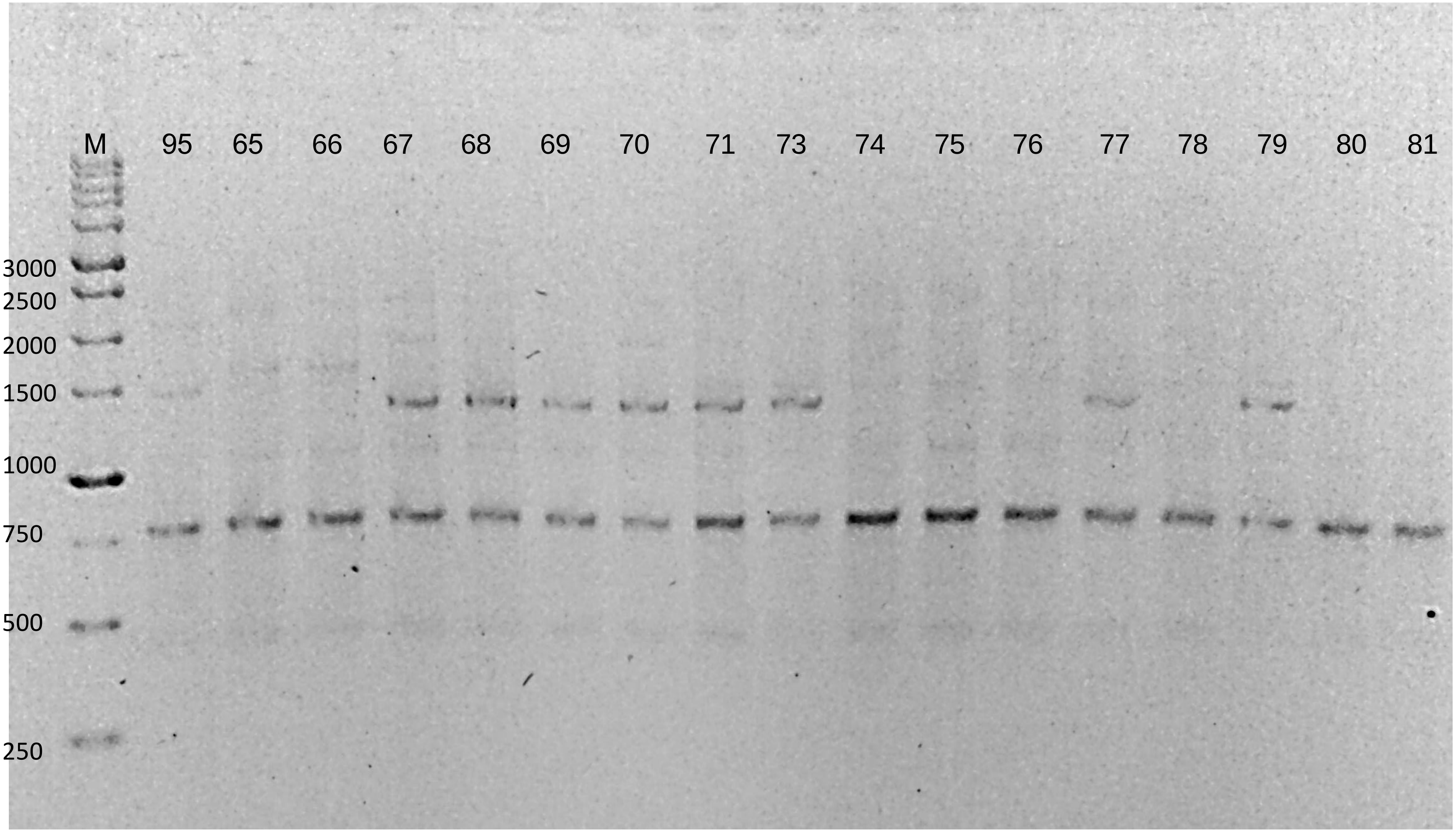
M
95
65
66
67
68
69
70
71
73
74
75
76
77
78
79
80
81
3000
2500
2000
1500
1000
750
500
250

SCoT23_82-94

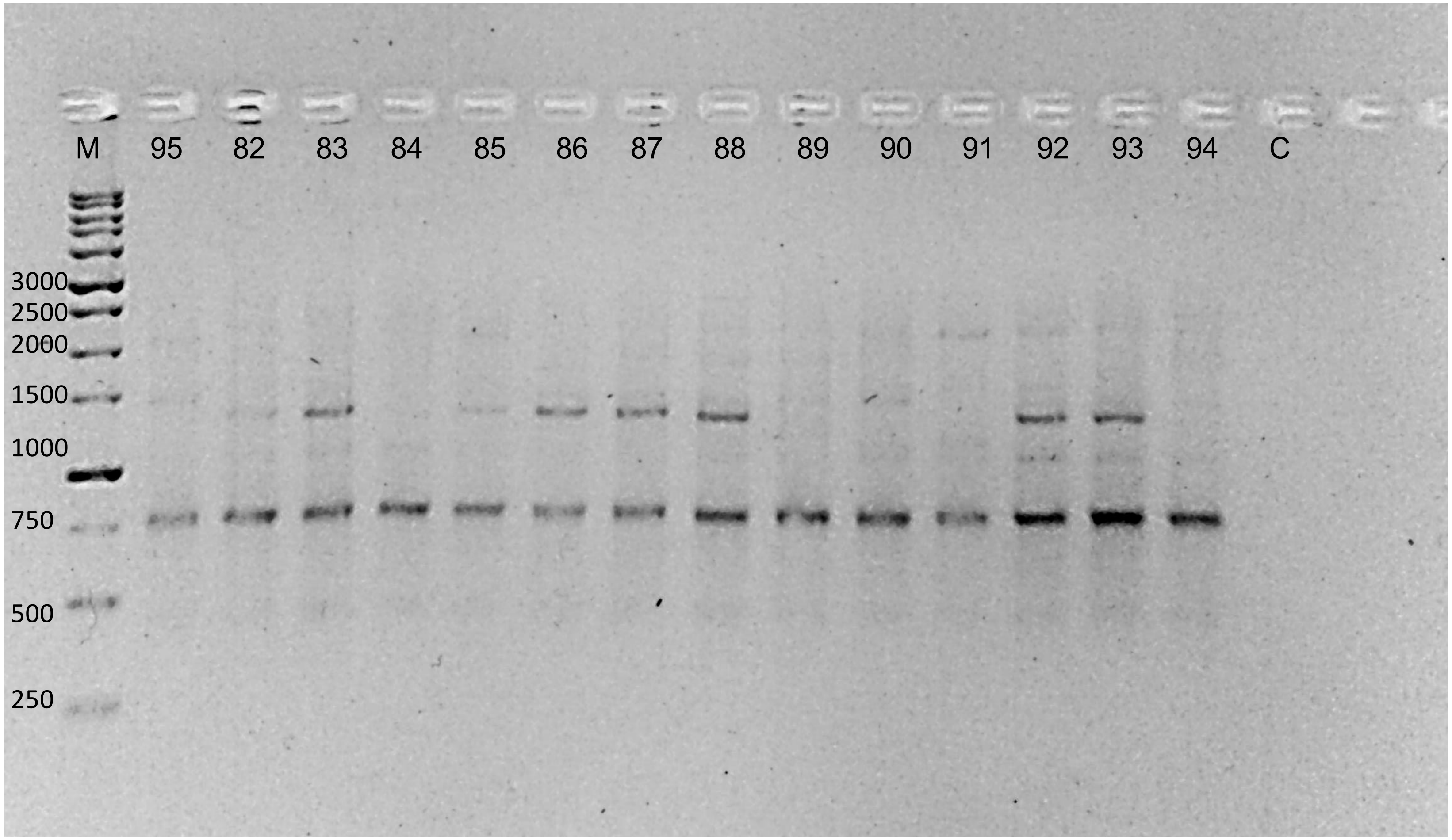
M
95
82
83
84
85
86
87
88
89
90
91
92
93
94
C
3000
2500
2000
1500
1000
750
500
250

SCoT29_1-16

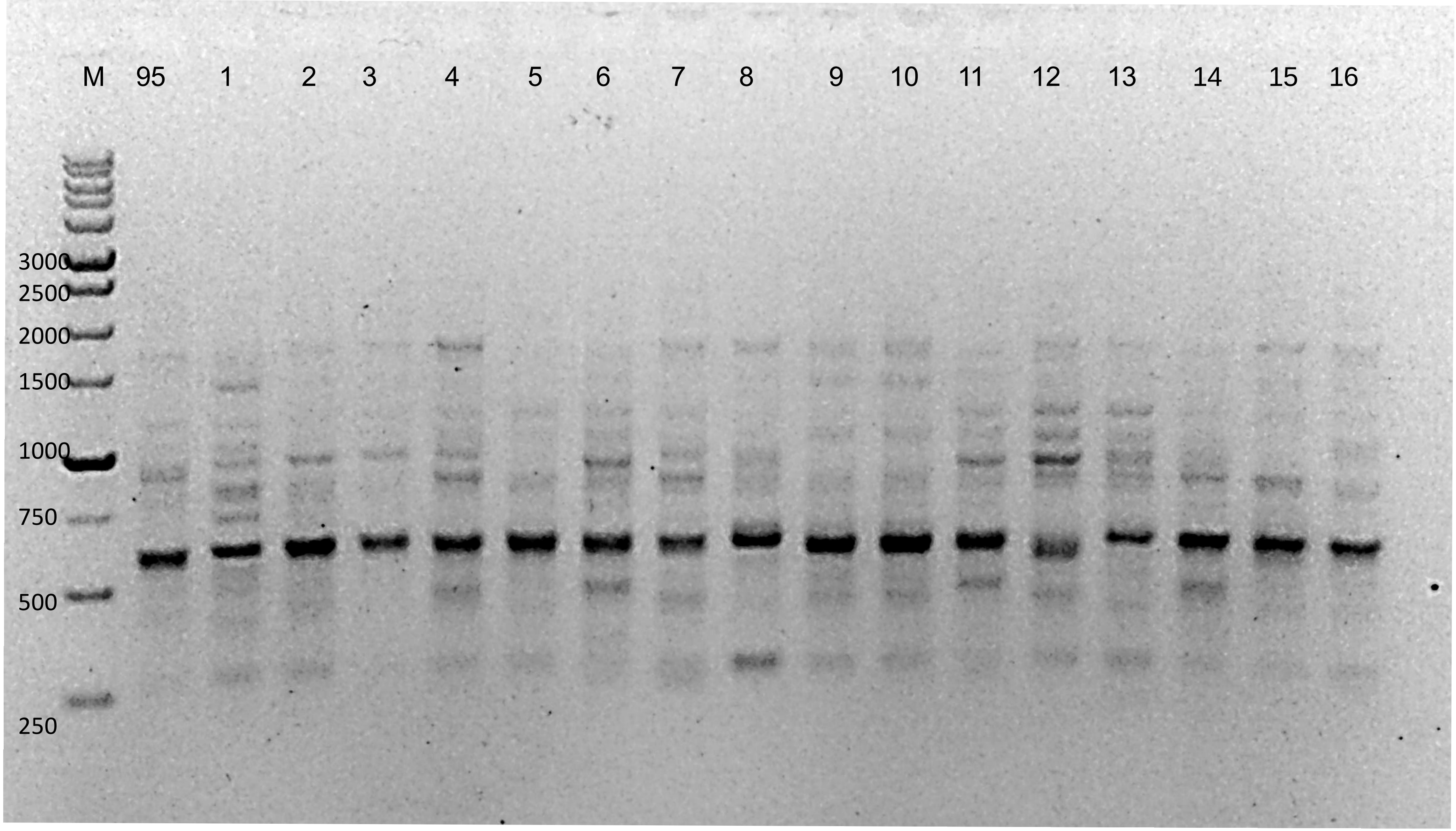

M 95 1 2 3 4 5 6 7 8 9 10 11 12 13 14 15 16
3000
2500
2000
1500
1000
750
500
250

SCoT29_17-32

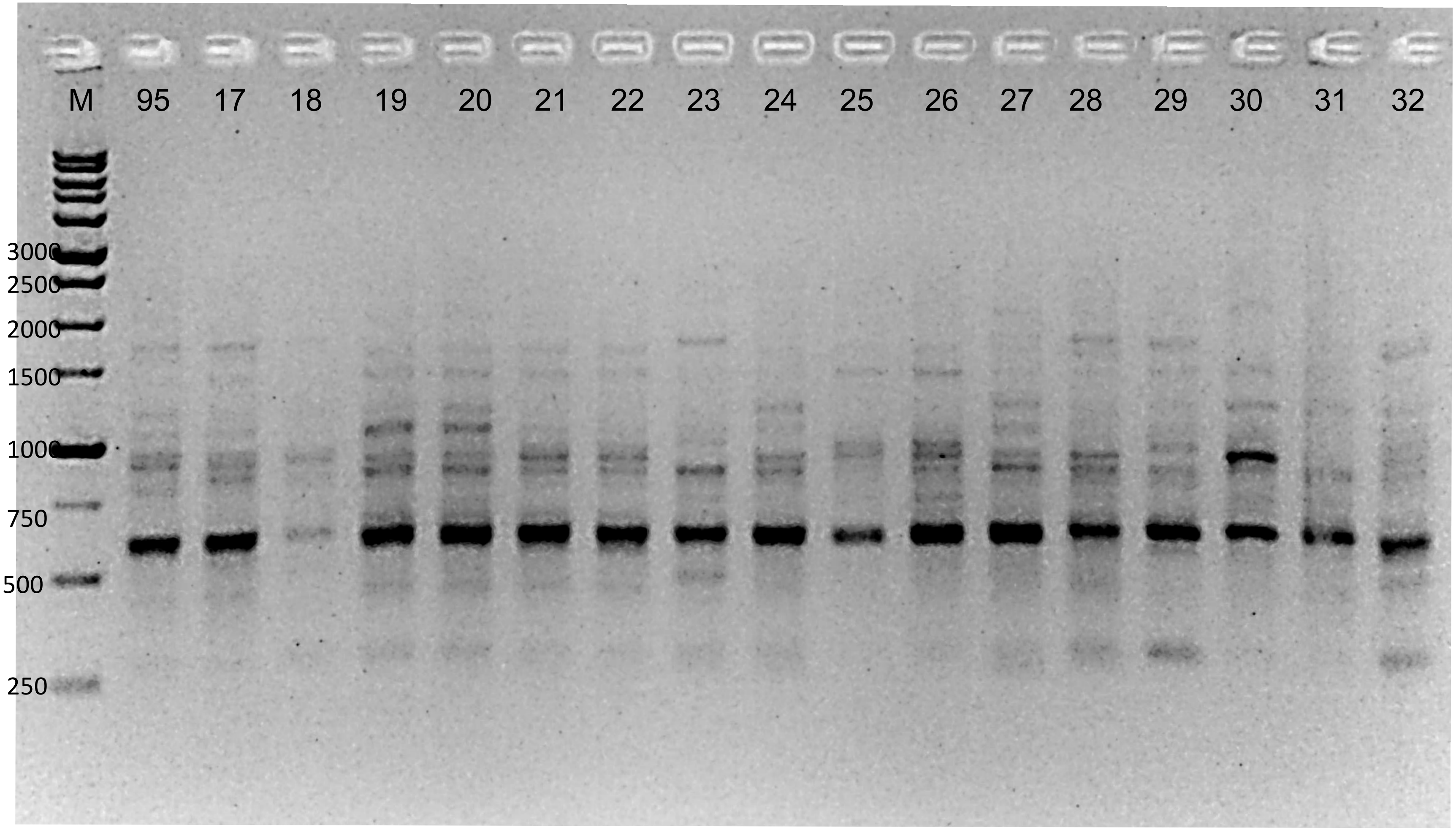
M
95
17
18
19
20
21
22
23
24
25
26
27
28
29
30
31
32
3000
2500
2000
1500
1000
750
500
250

SCoT29_33-48

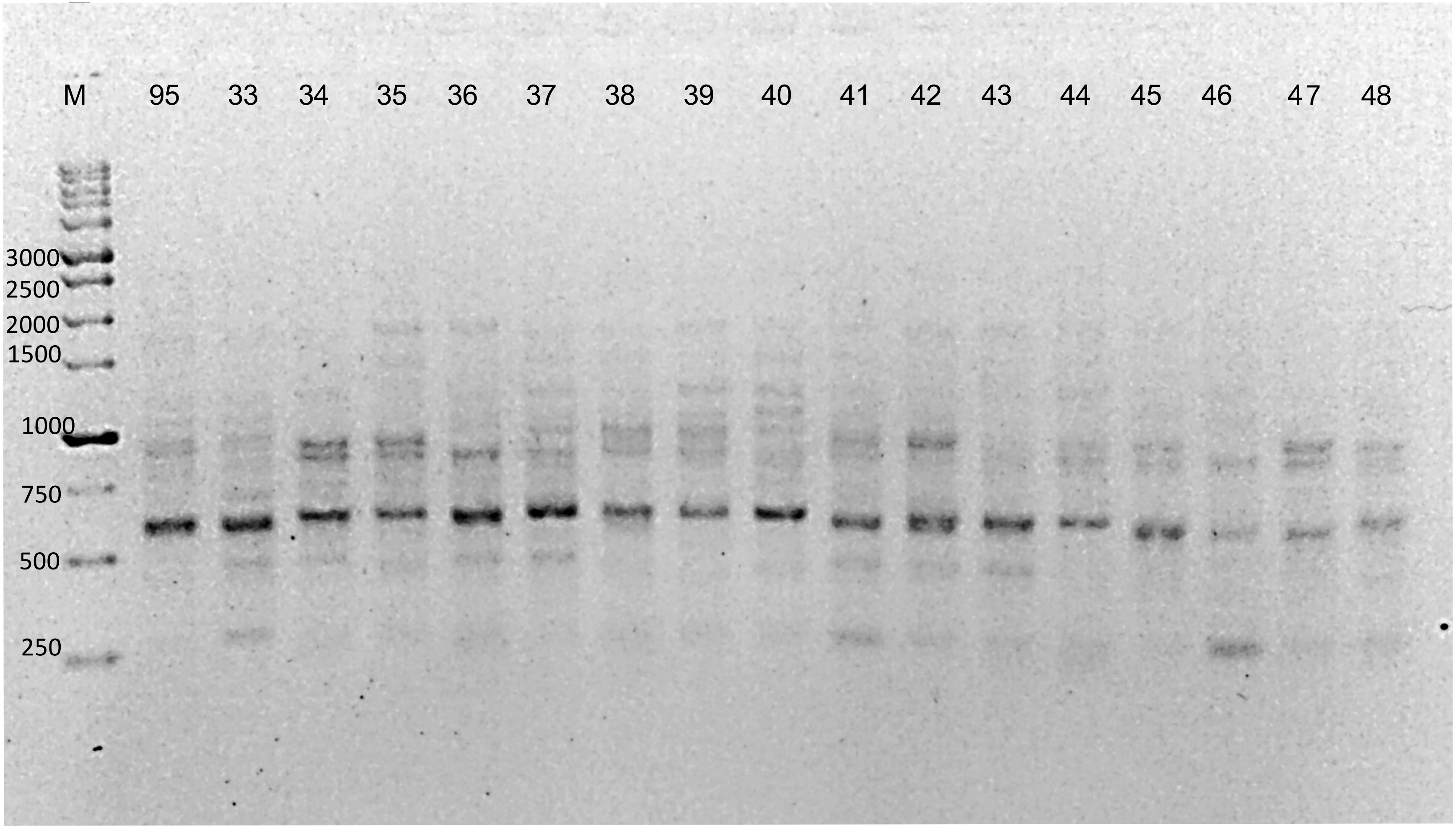
M
95
33
34
35
36
37
38
39
40
41
42
43
44
45
46
47
48
3000
2500
2000
1500
1000
750
500
250

SCoT29_49-64

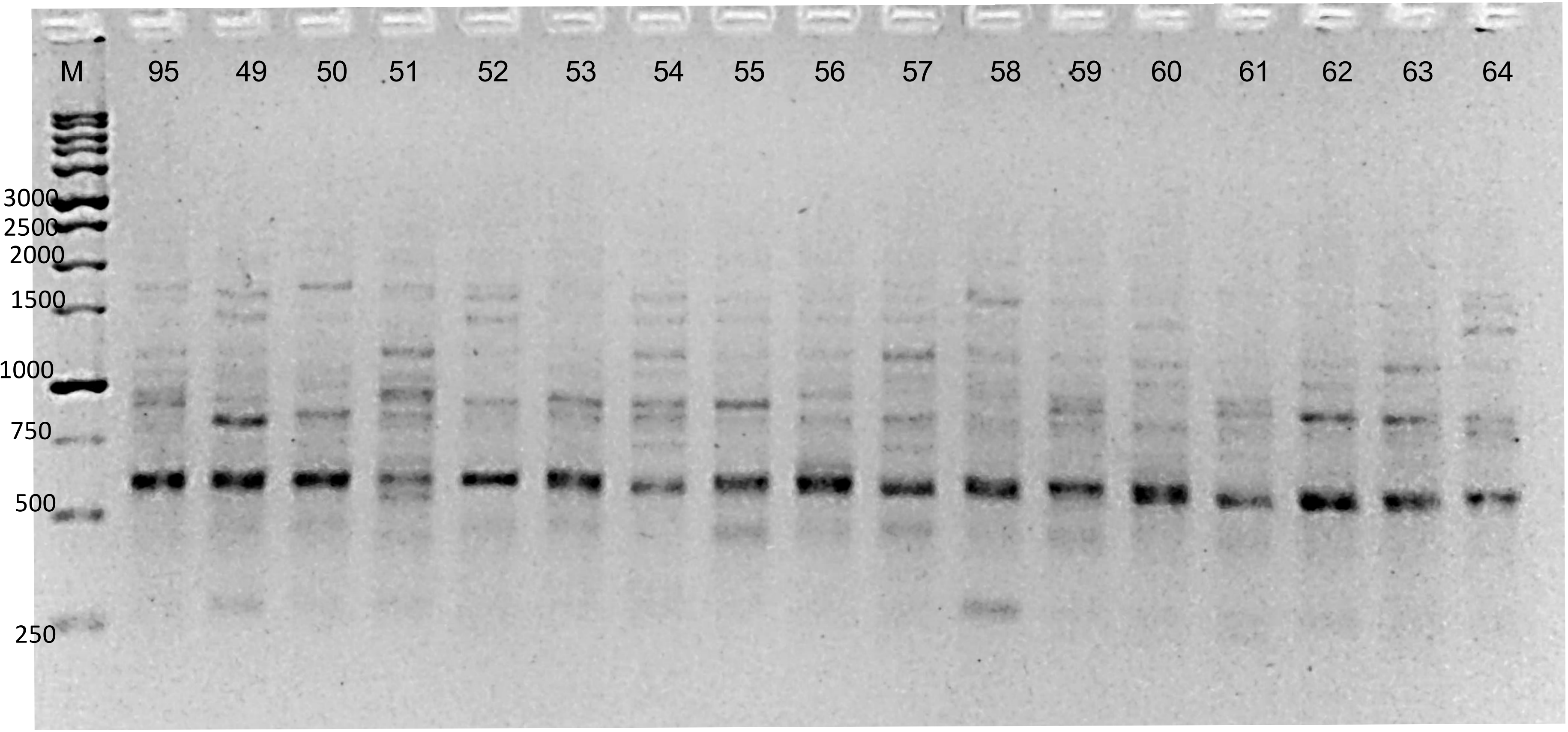
M
95
49
50
51
52
53
54
55
56
57
58
59
60
61
62
63
64
3000
2500
2000
1500
1000
750
500
250

SCoT29_65-81

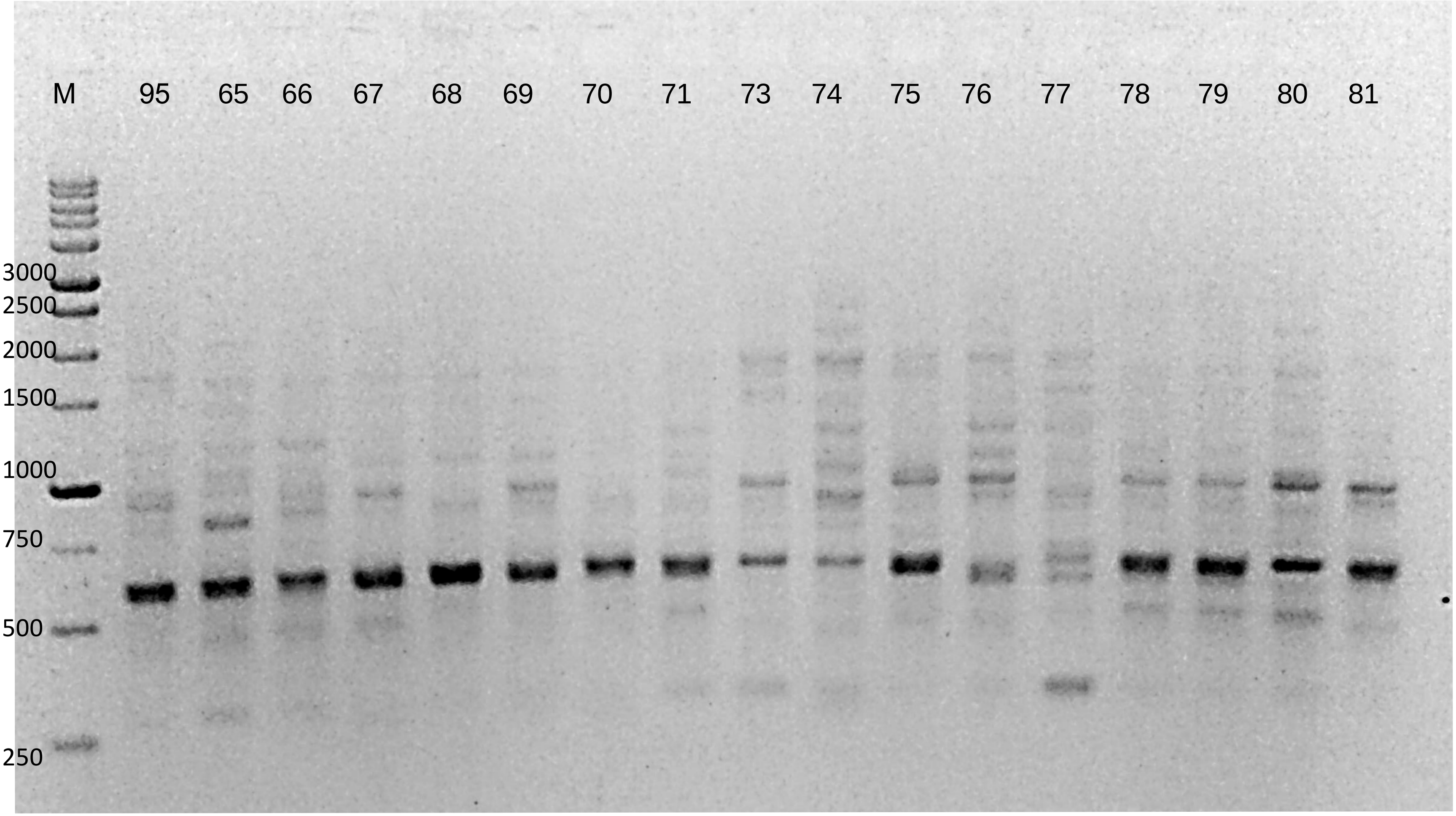
M
95
65
66
67
68
69
70
71
73
74
75
76
77
78
79
80
81
3000
2500
2000
1500
1000
750
500
250

SCoT29_82-94

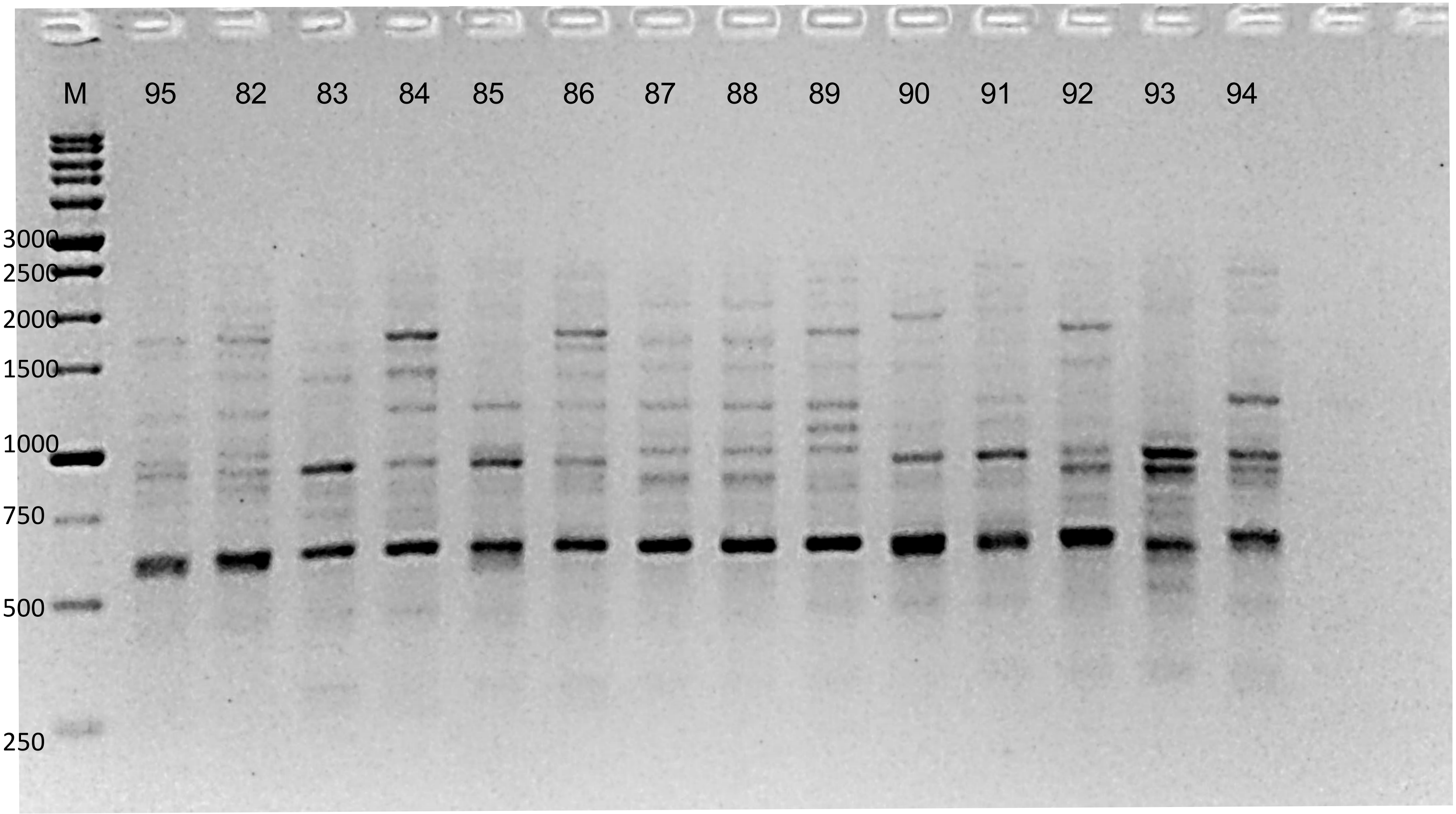
M
95
82
83
84
85
86
87
88
89
90
91
92
93
94
3000
2500
2000
1500
1000
750
500
250

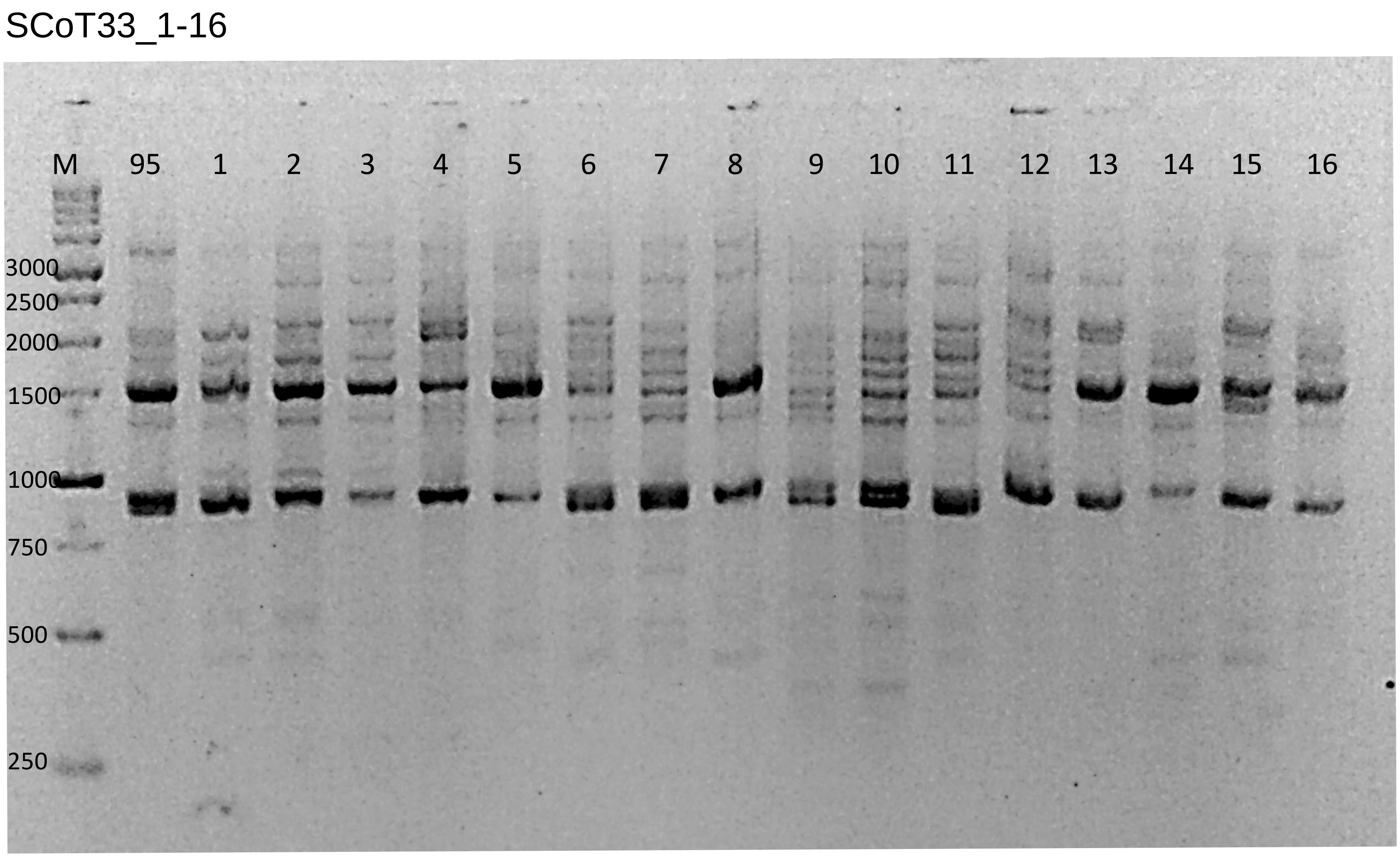
SCoT33_1-16
M
95
1
2
3
4
5
6
7
8
9
10
11
12
13
14
15
16
3000
2500
2000
1500
1000
750
500
250

SCoT33_17-32

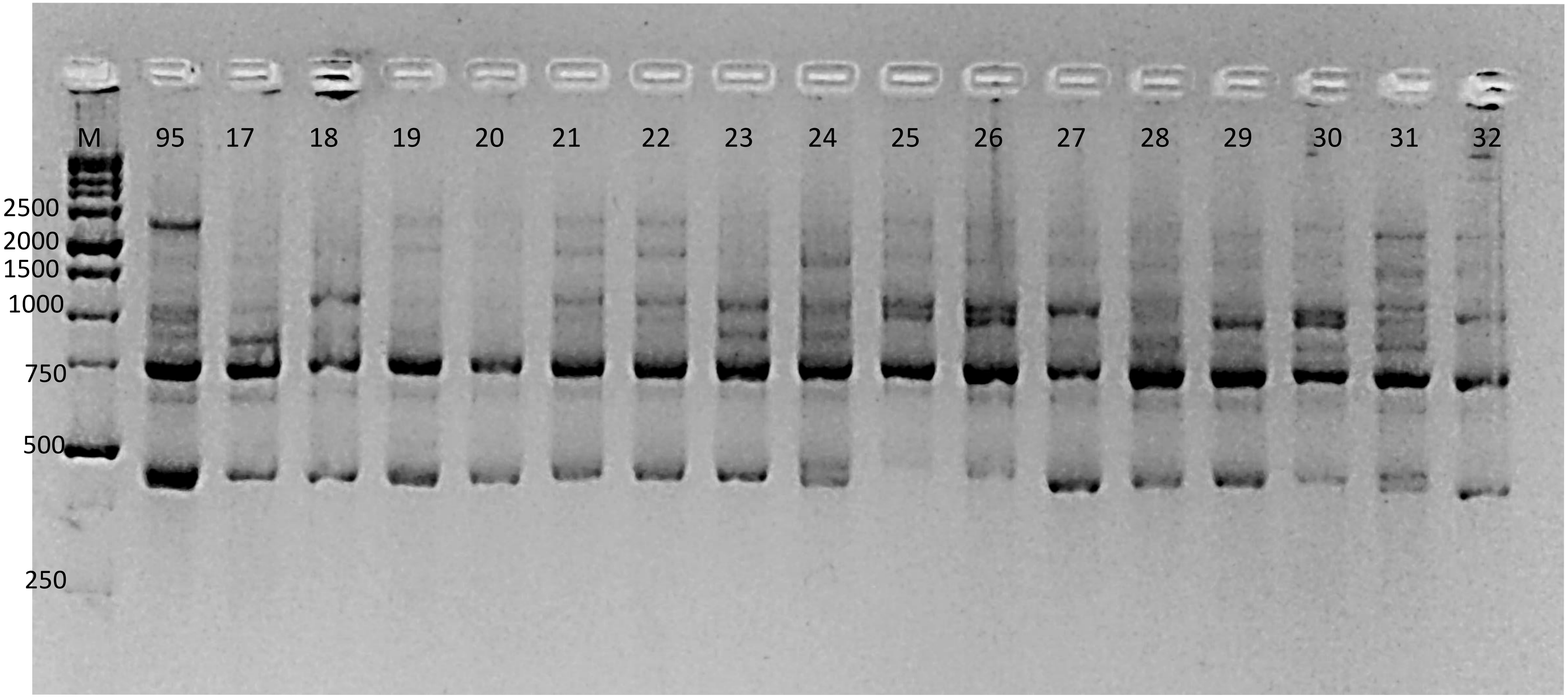
M 95 17 18 19 20 21 22 23 24 25 26 27 28 29 30 31 32
2500
2000
1500
1000
750
500
250

SCoT33_33-48

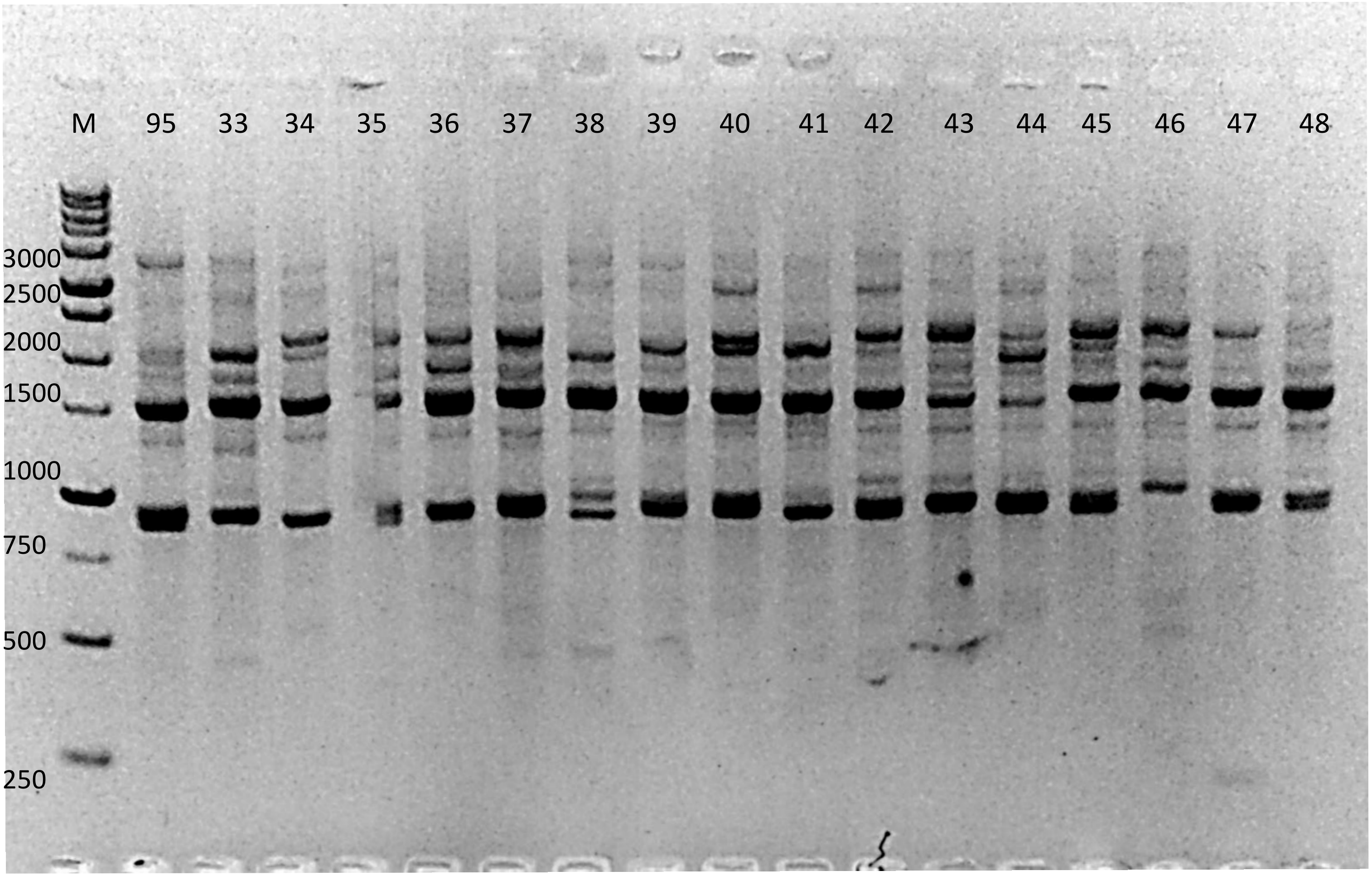
M
95
33
34
35
36
37
38
39
40
41
42
43
44
45
46
47
48
3000
2500
2000
1500
1000
750
500
250

SCoT33_49-64

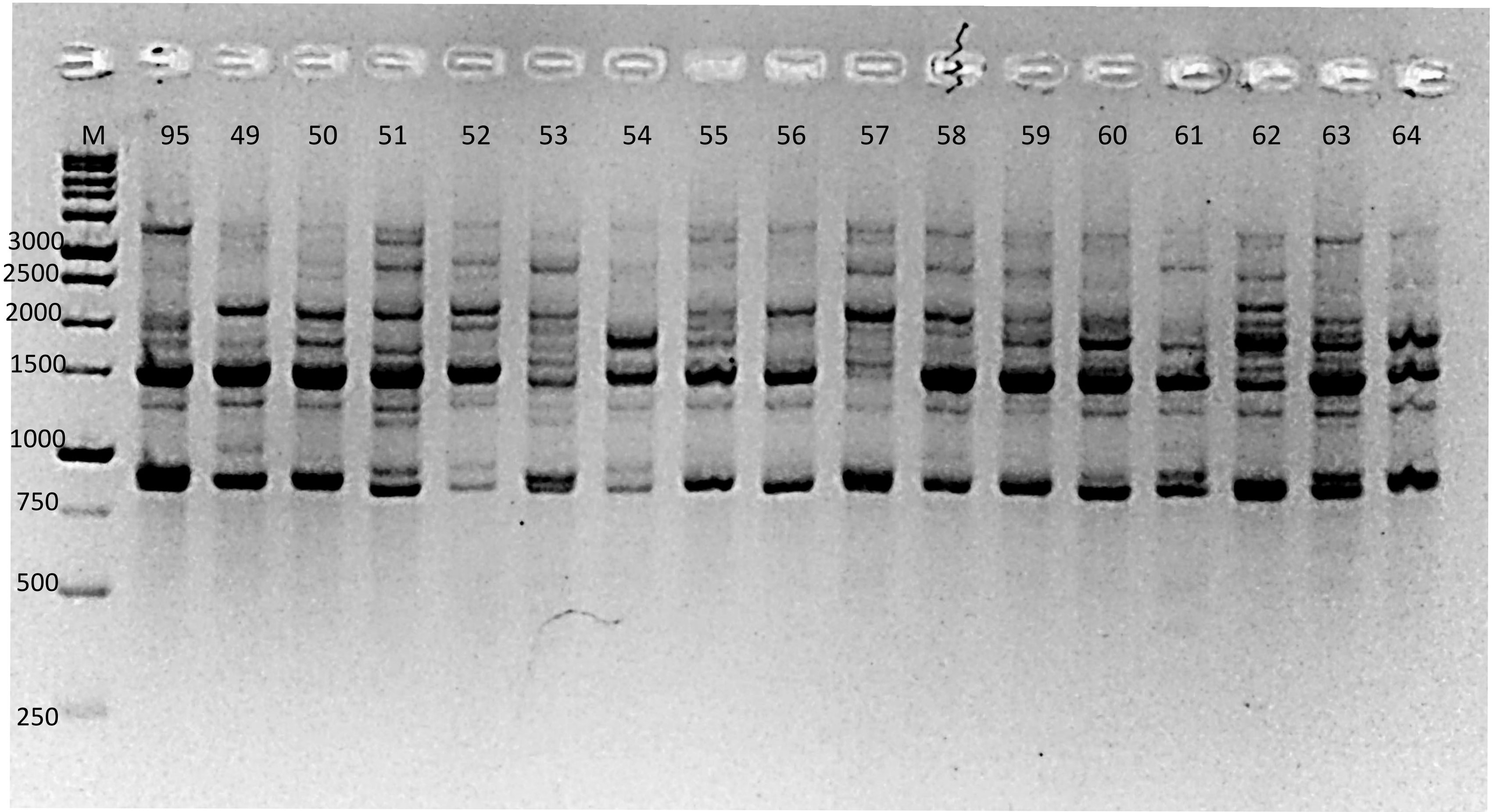
M
95
49
50
51
52
53
54
55
56
57
58
59
60
61
62
63
64
3000
2500
2000
1500
1000
750
500
250

SCoT33_65-81

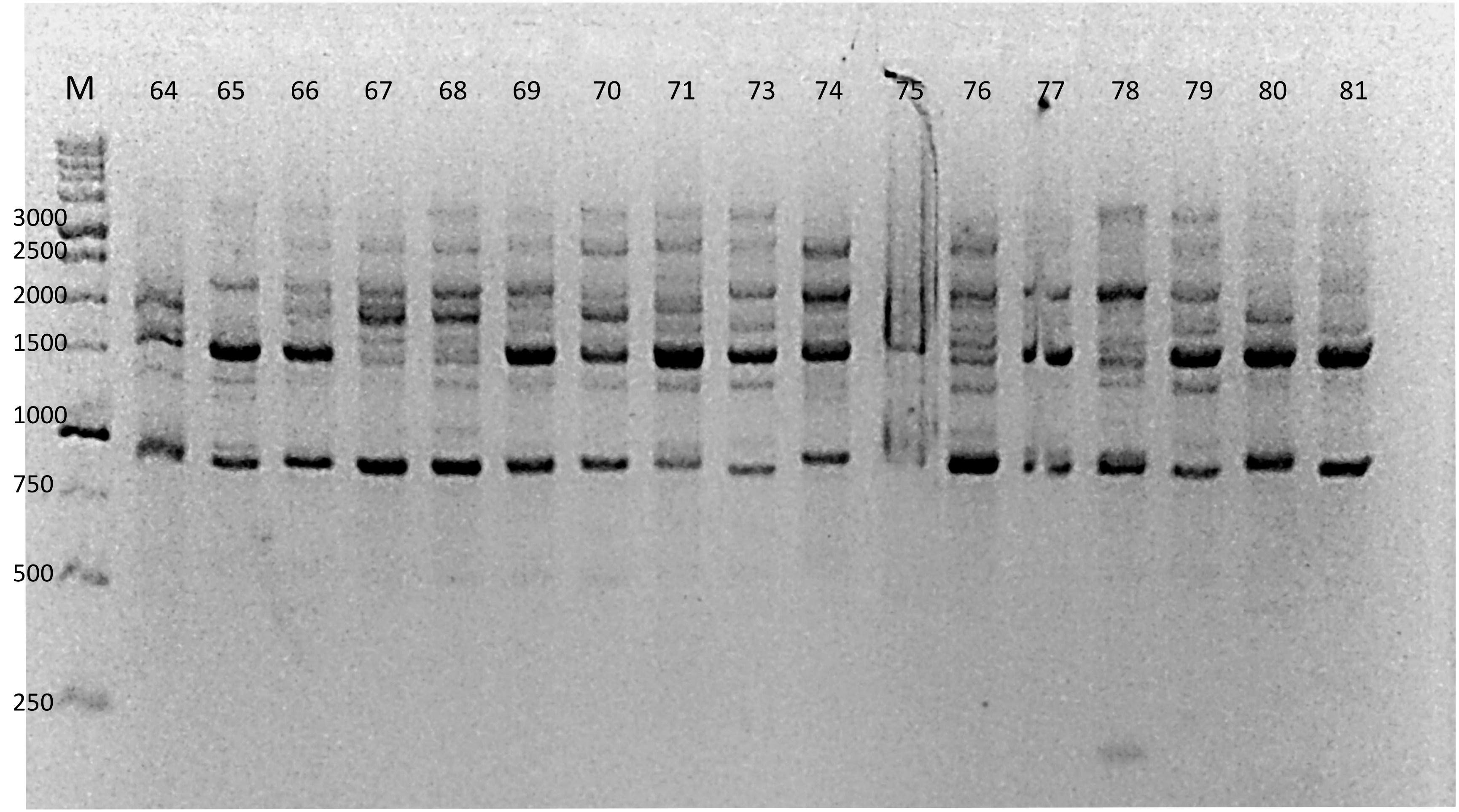
M
64
65
66
67
68
69
70
71
73
74
75
76
77
78
79
80
81
3000
2500
2000
1500
1000
750
500
250

SCoT33_82-94

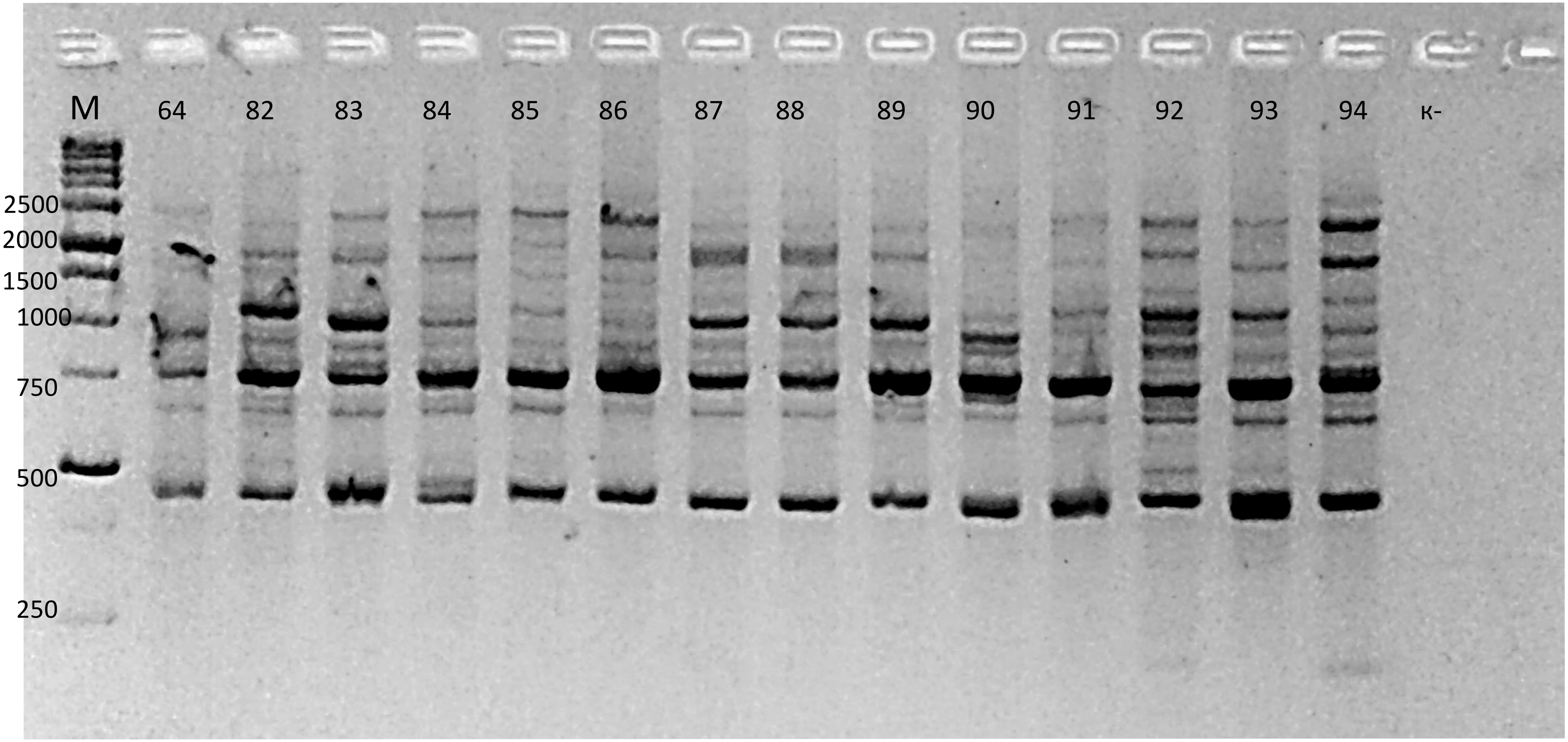

M
64
82
83
84
85
86
87
88
89
90
91
92
93
94
к-
2500
2000
1500
1000
750
500
250

SCoT31_1-16

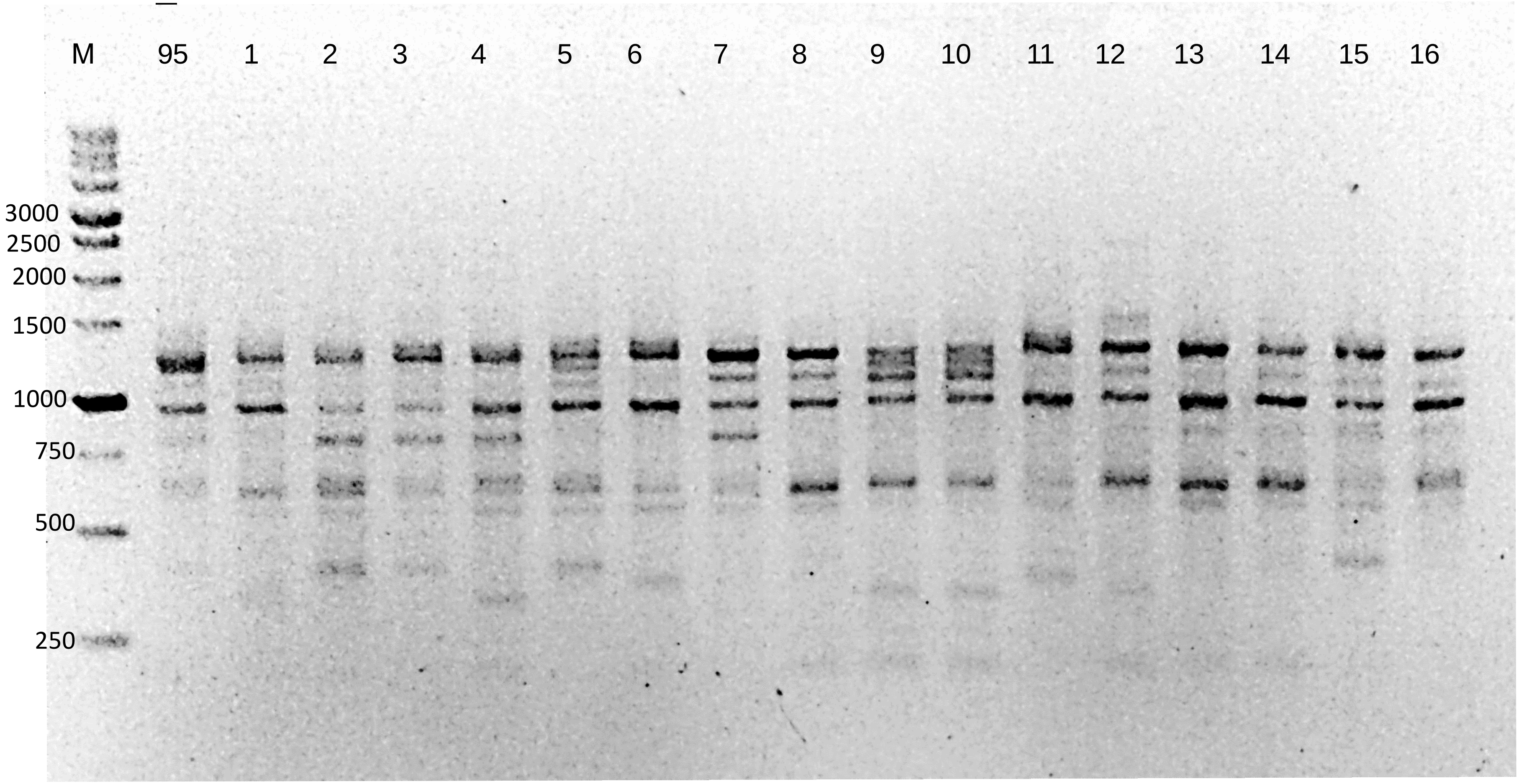
M
95
1
2
3
4
5
6
7
8
9
10
11
12
13
14
15
16
3000
2500
2000
1500
1000
750
500
250

SCoT31_17-32

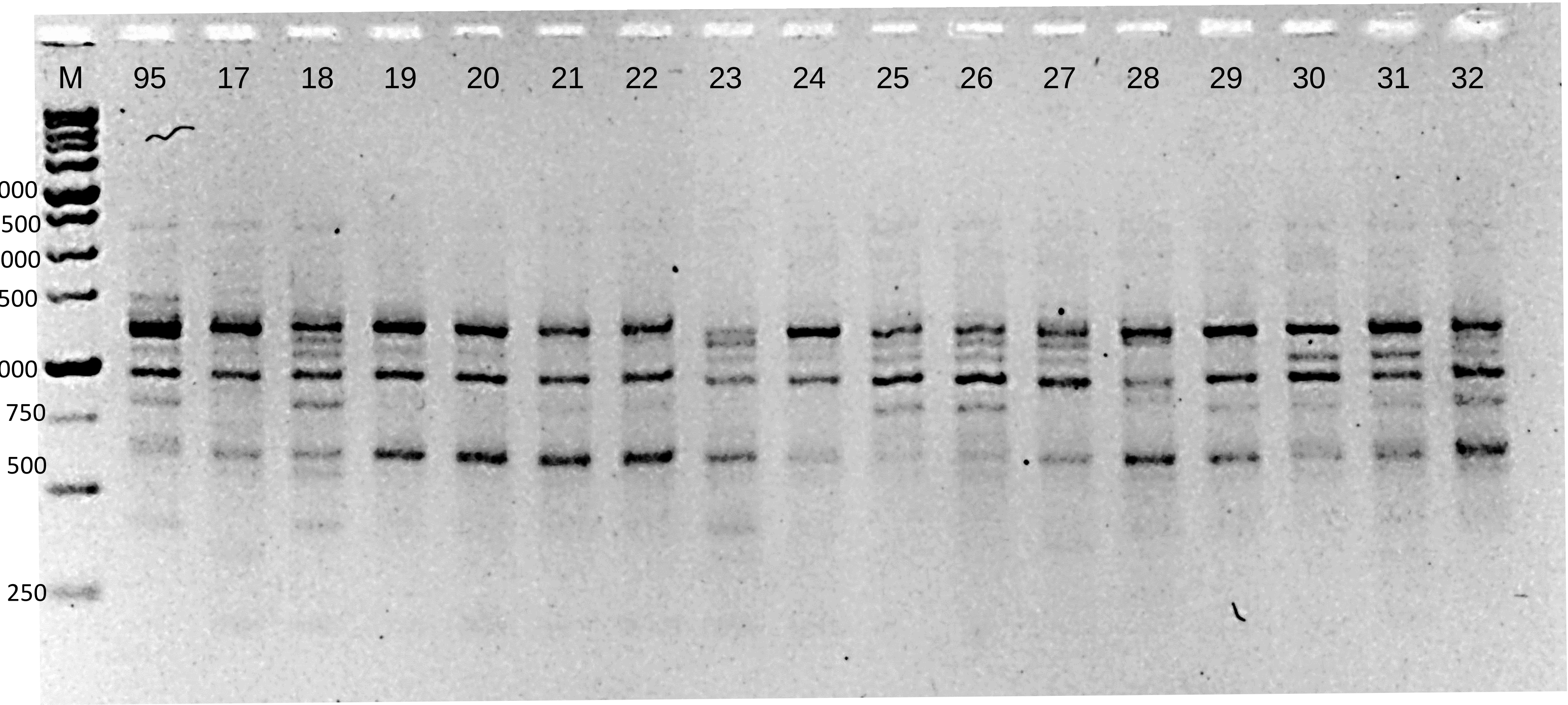
M
95
17
18
19
20
21
22
23
24
25
26
27
28
29
30
31
32
000
500
000
500
000
750
500
250

SCoT31_33-48

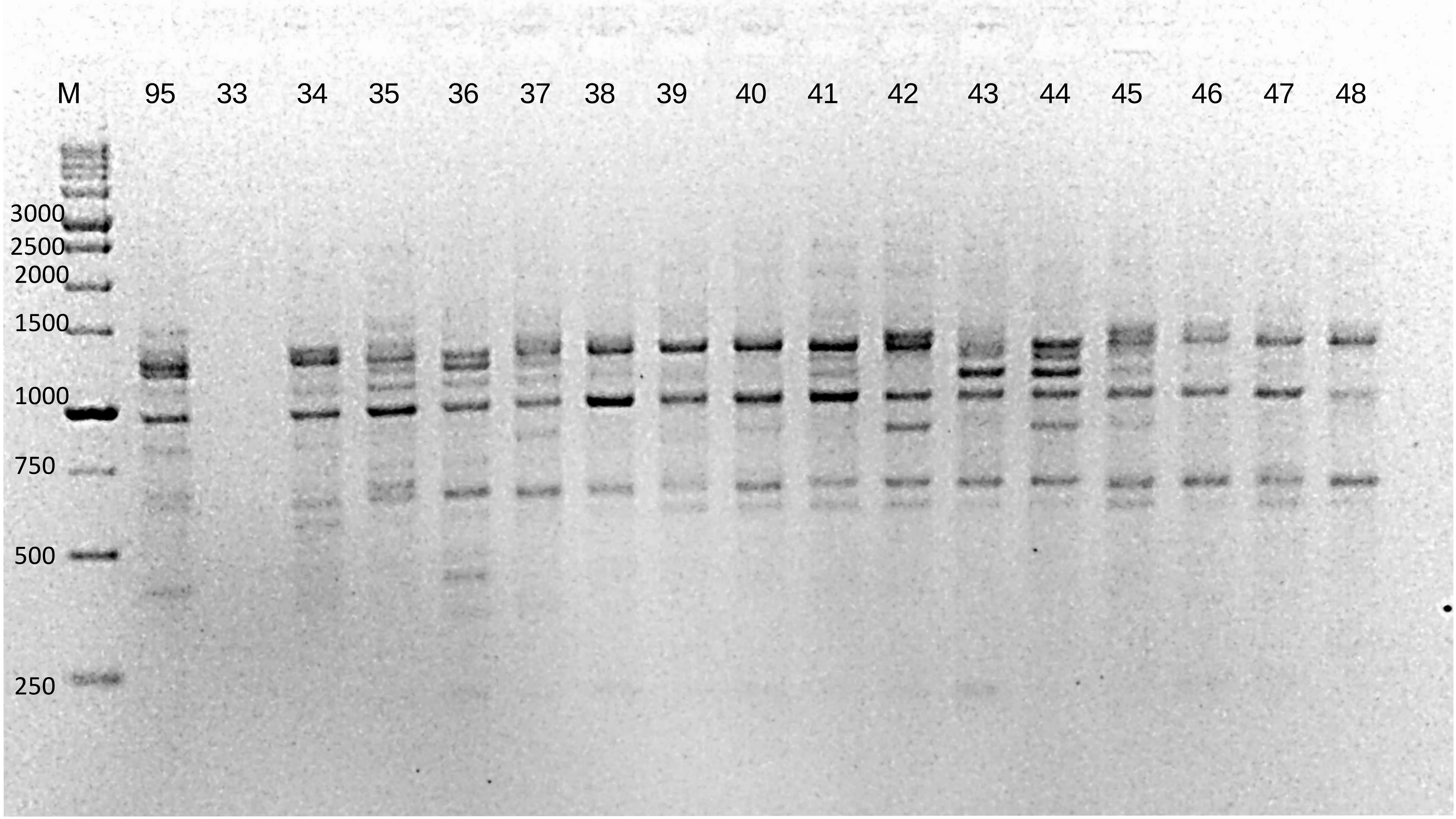
M
95
33
34
35
36
37
38
39
40
41
42
43
44
45
46
47
48
3000
2500
2000
1500
1000
750
500
250

SCoT31_49-64

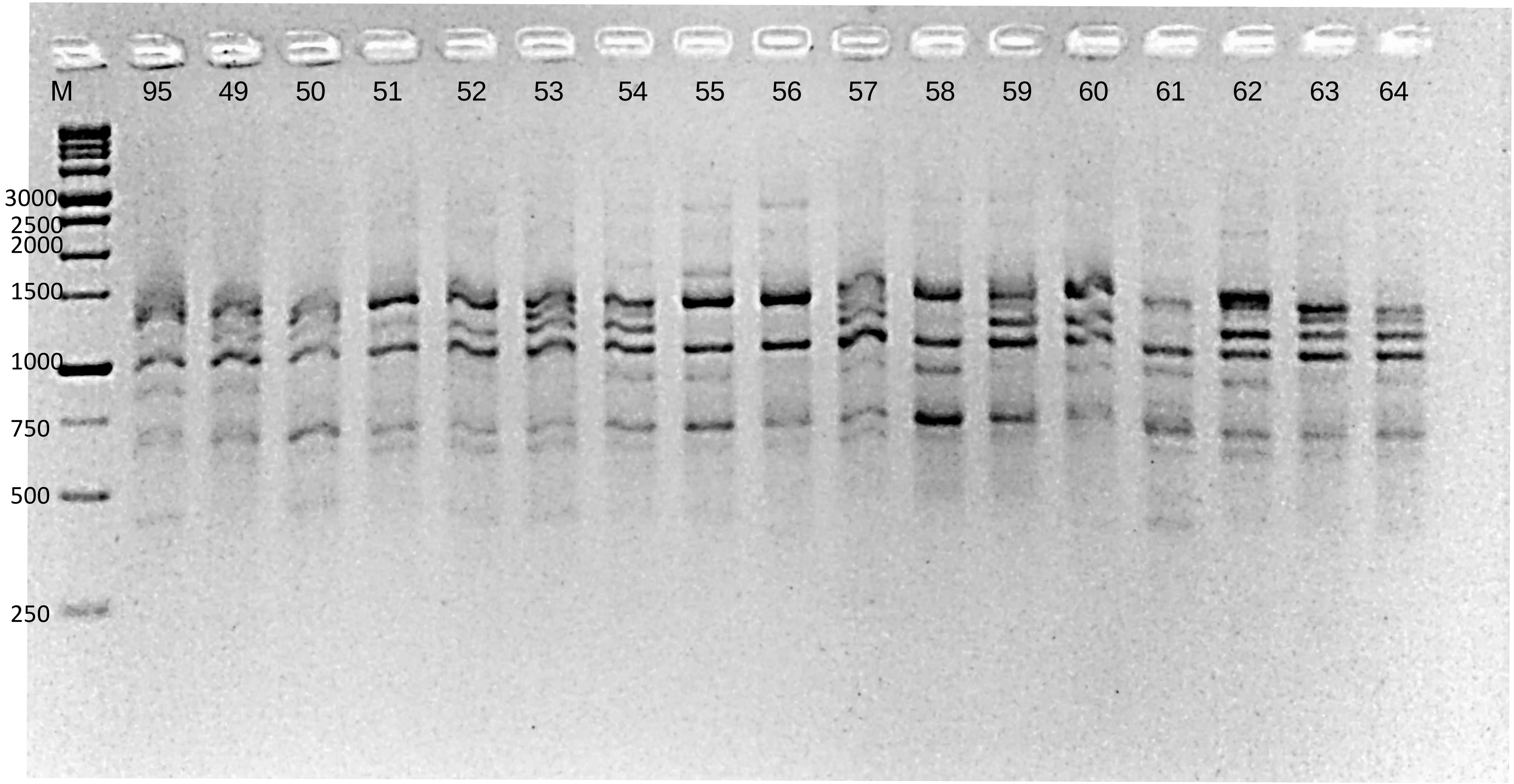
M
95
49
50
51
52
53
54
55
56
57
58
59
60
61
62
63
64
3000
2500
2000
1500
1000
750
500
250

SCoT31 65-81

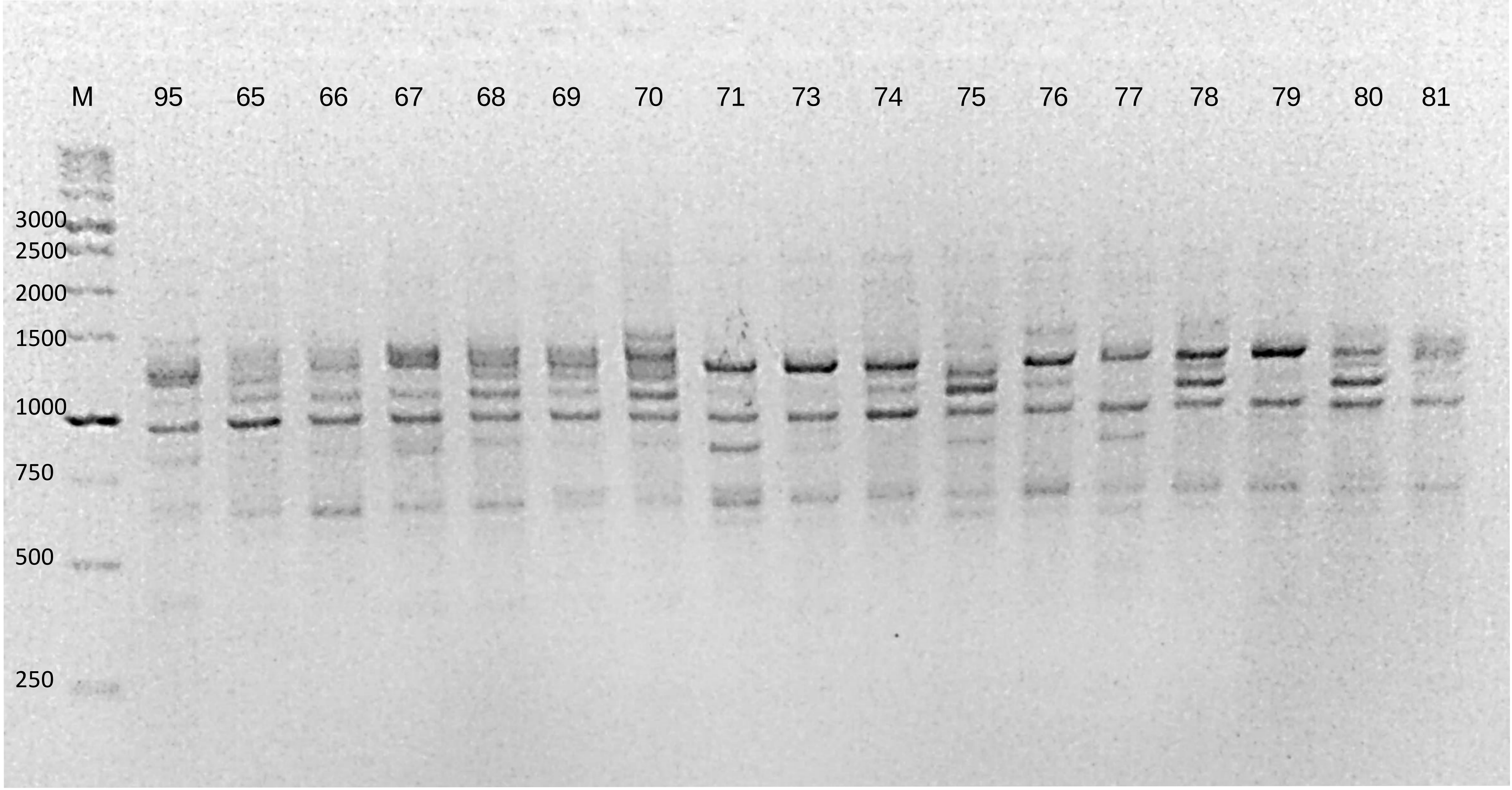
M
95
65
66
67
68
69
70
71
73
74
75
76
77
78
79
80
81
3000
2500
2000
1500
1000
750
500
250

SCoT31_82-94

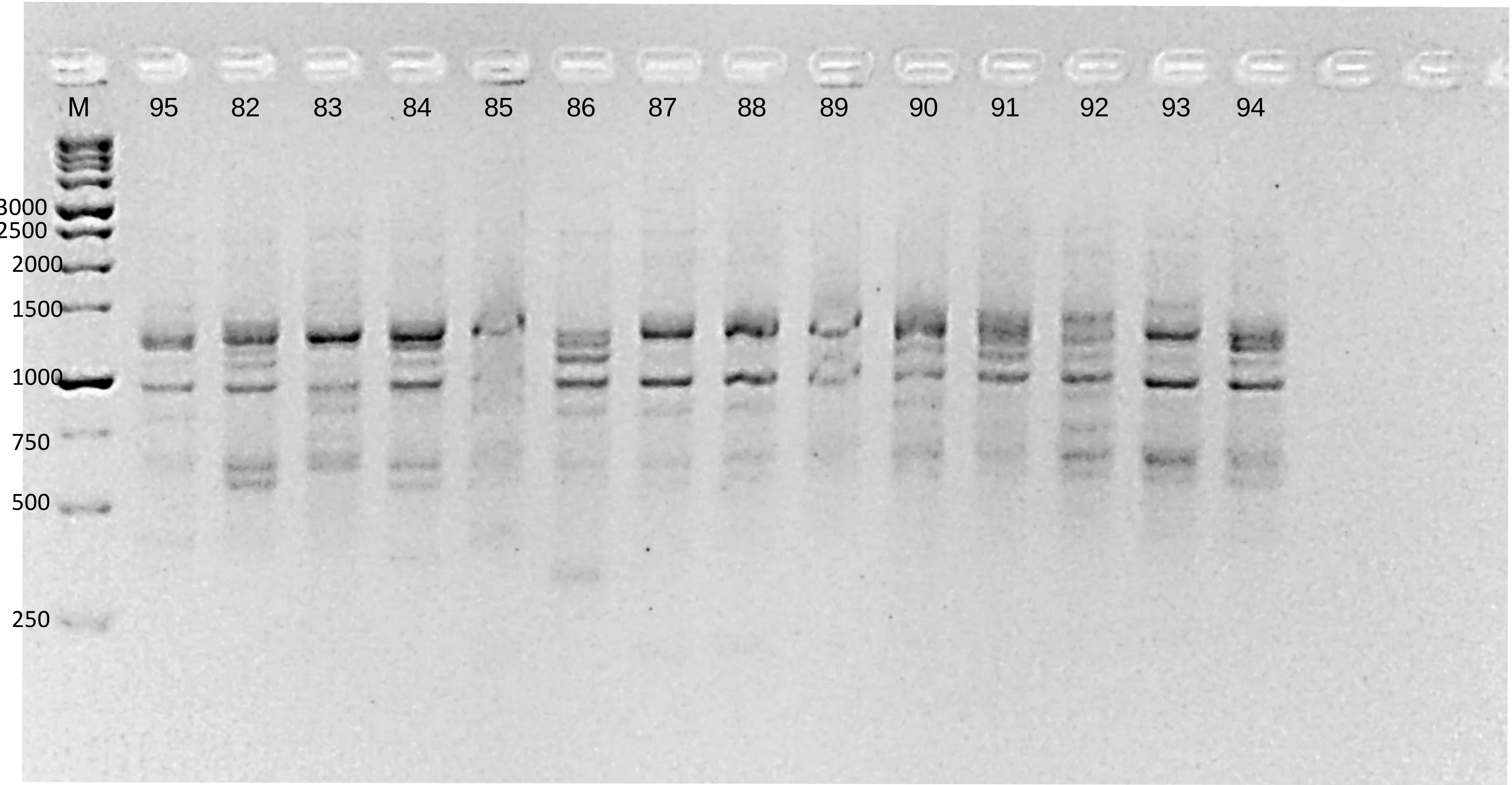
M
95
82
83
84
85
86
87
88
89
90
91
92
93
94
3000
2500
2000
1500
1000
750
500
250

SCoT34_1-16

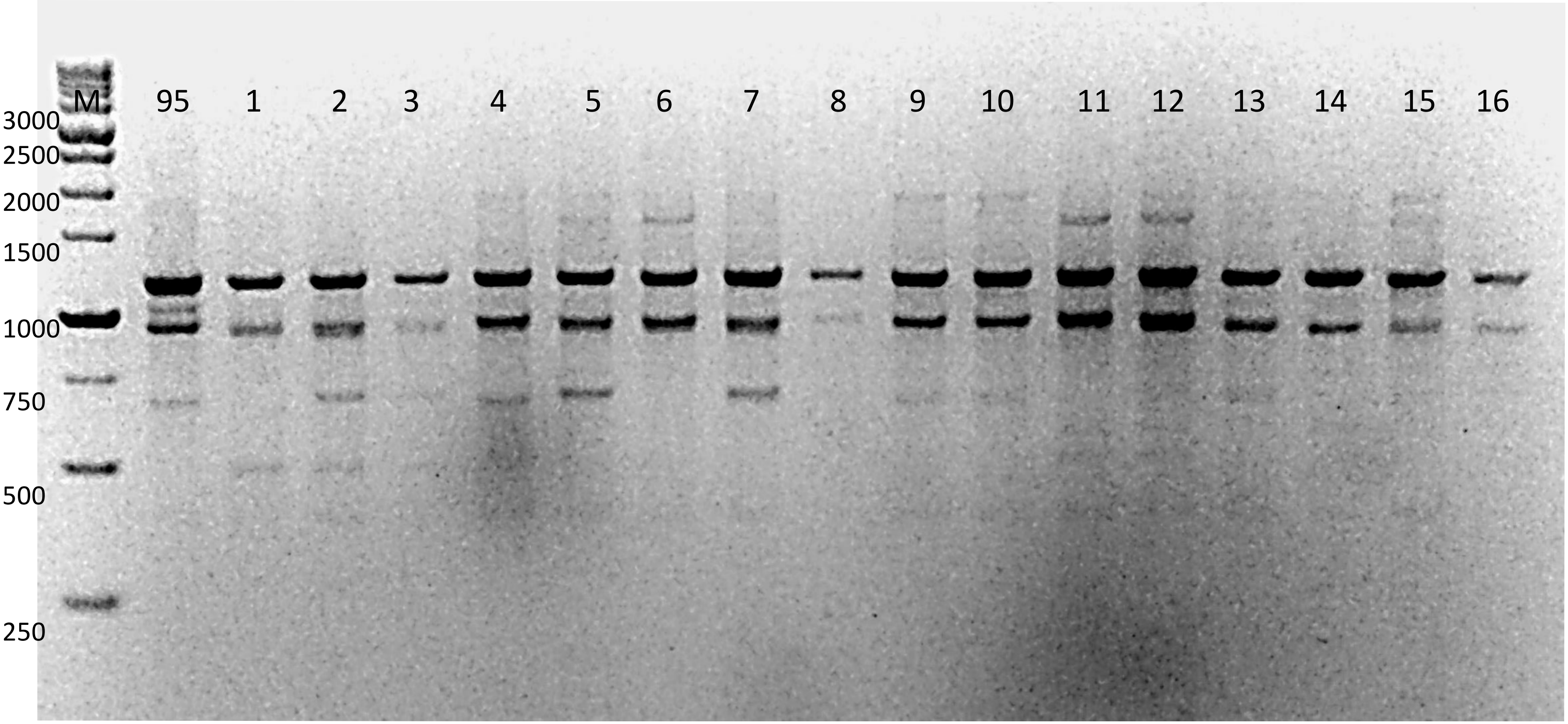
M
95
1
2
3
4
5
6
7
8
9
10
11
12
13
14
15
16
3000
2500
2000
1500
1000
750
500
250

SCoT34_17-32

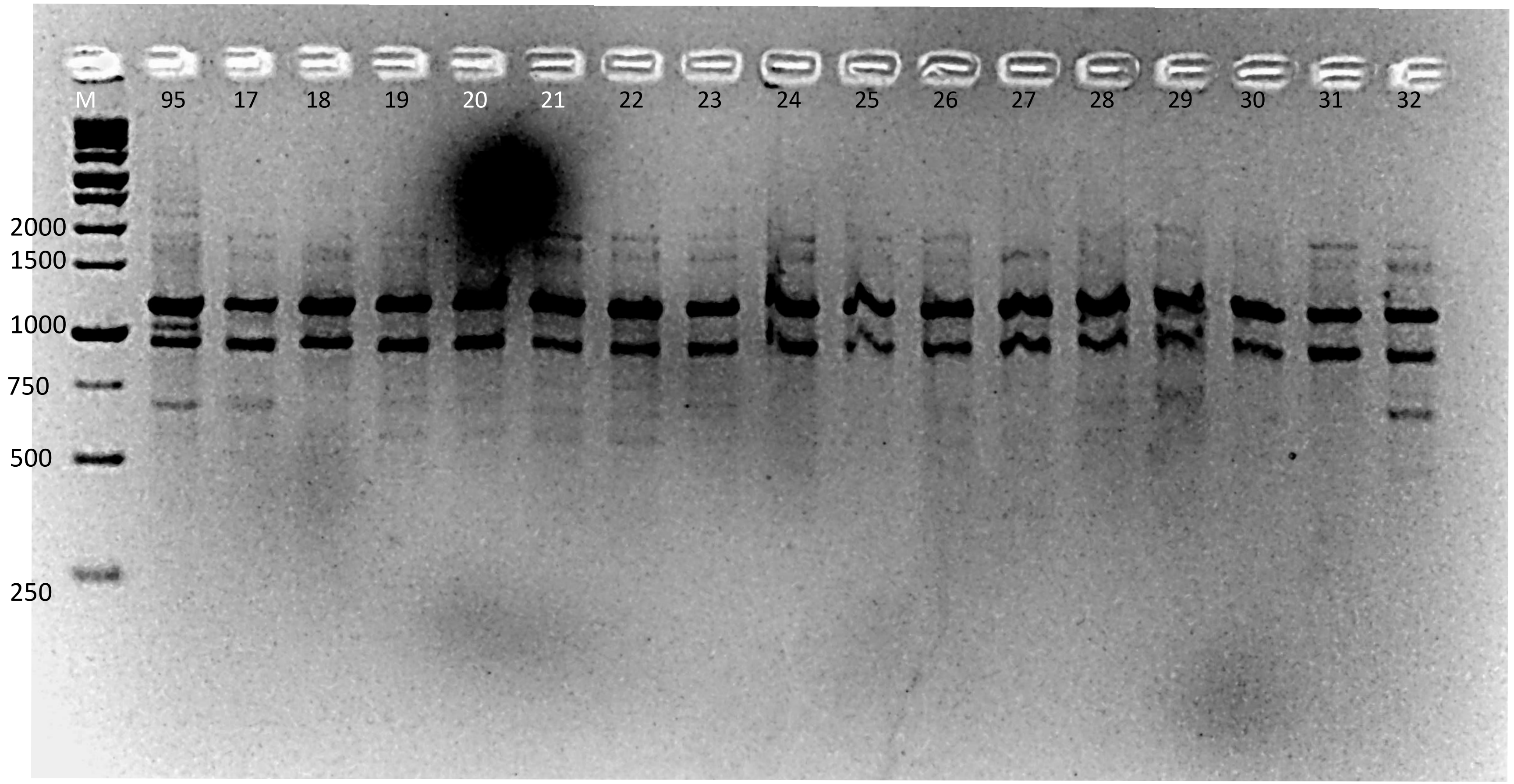
M
95
17
18
19
20
21
22
23
24
25
26
27
28
29
30
31
32
2000
1500
1000
750
500
250

SCoT34_33-48

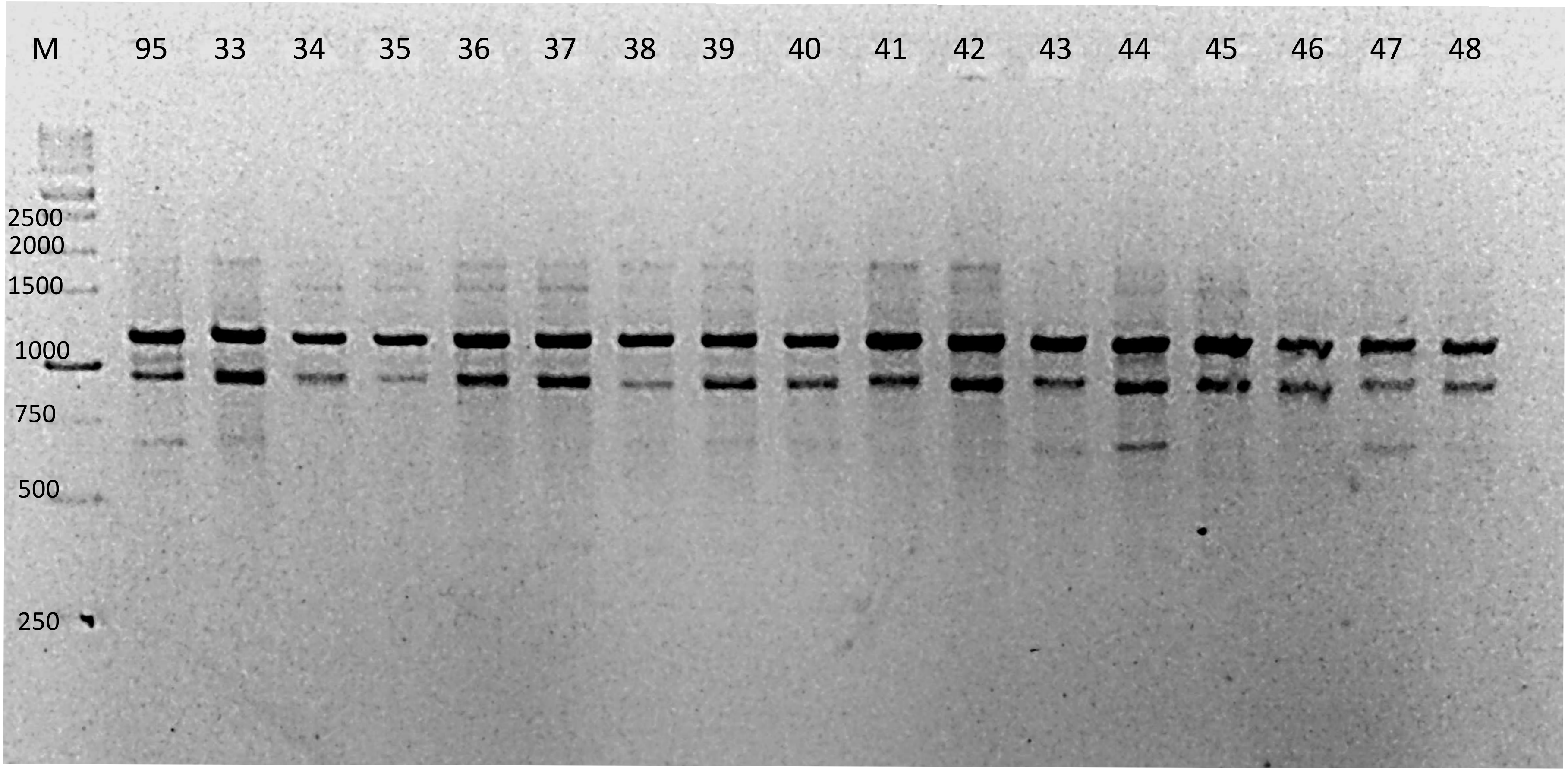

M
95
33
34
35
36
37
38
39
40
41
42
43
44
45
46
47
48
2500
2000
1500
1000
750
500
250

SCoT34_49-64

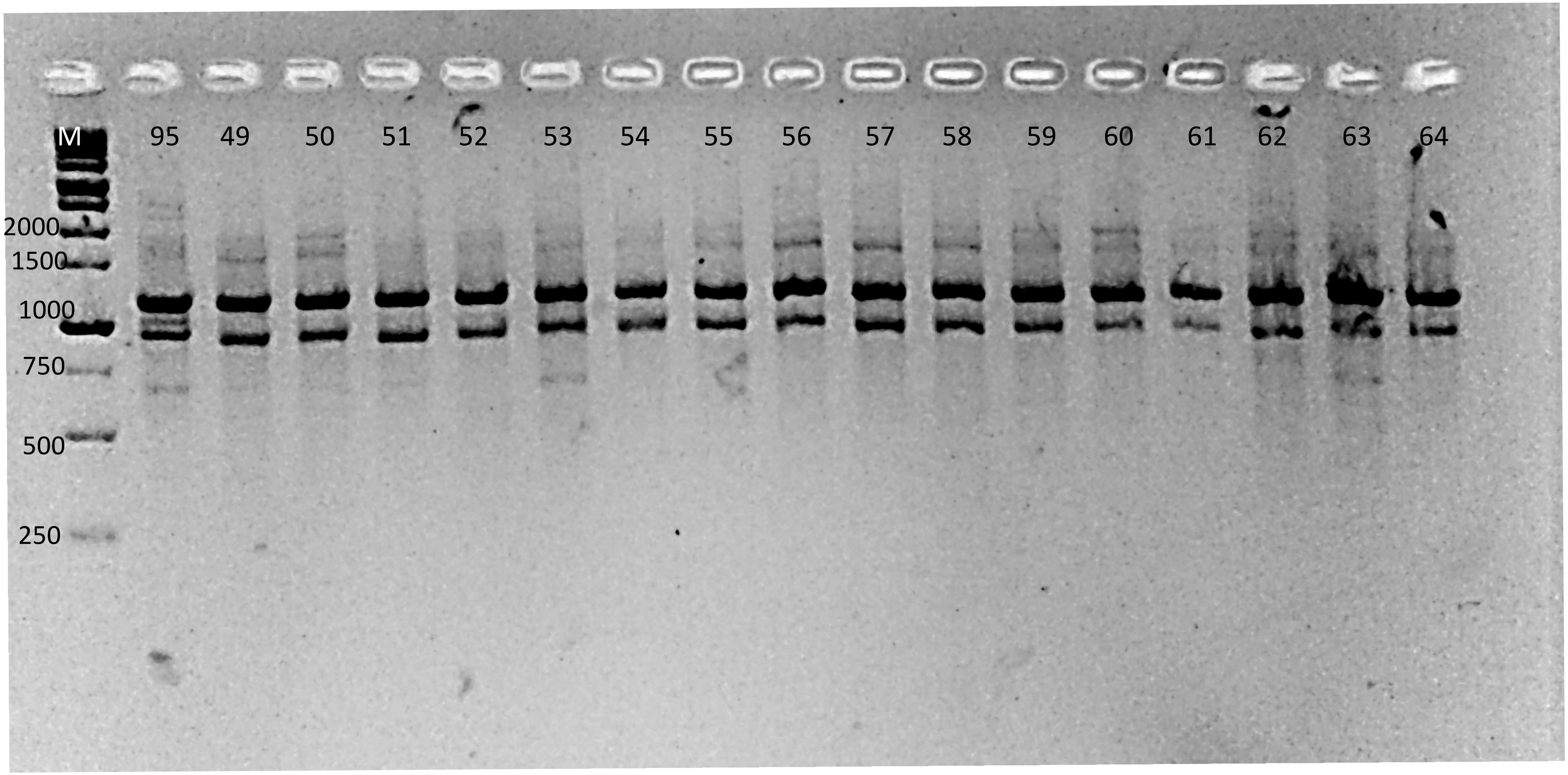
M
95
49
50
51
52
53
54
55
56
57
58
59
60
61
62
63
64
2000
1500
1000
750
500
250

SCoT34_65-81

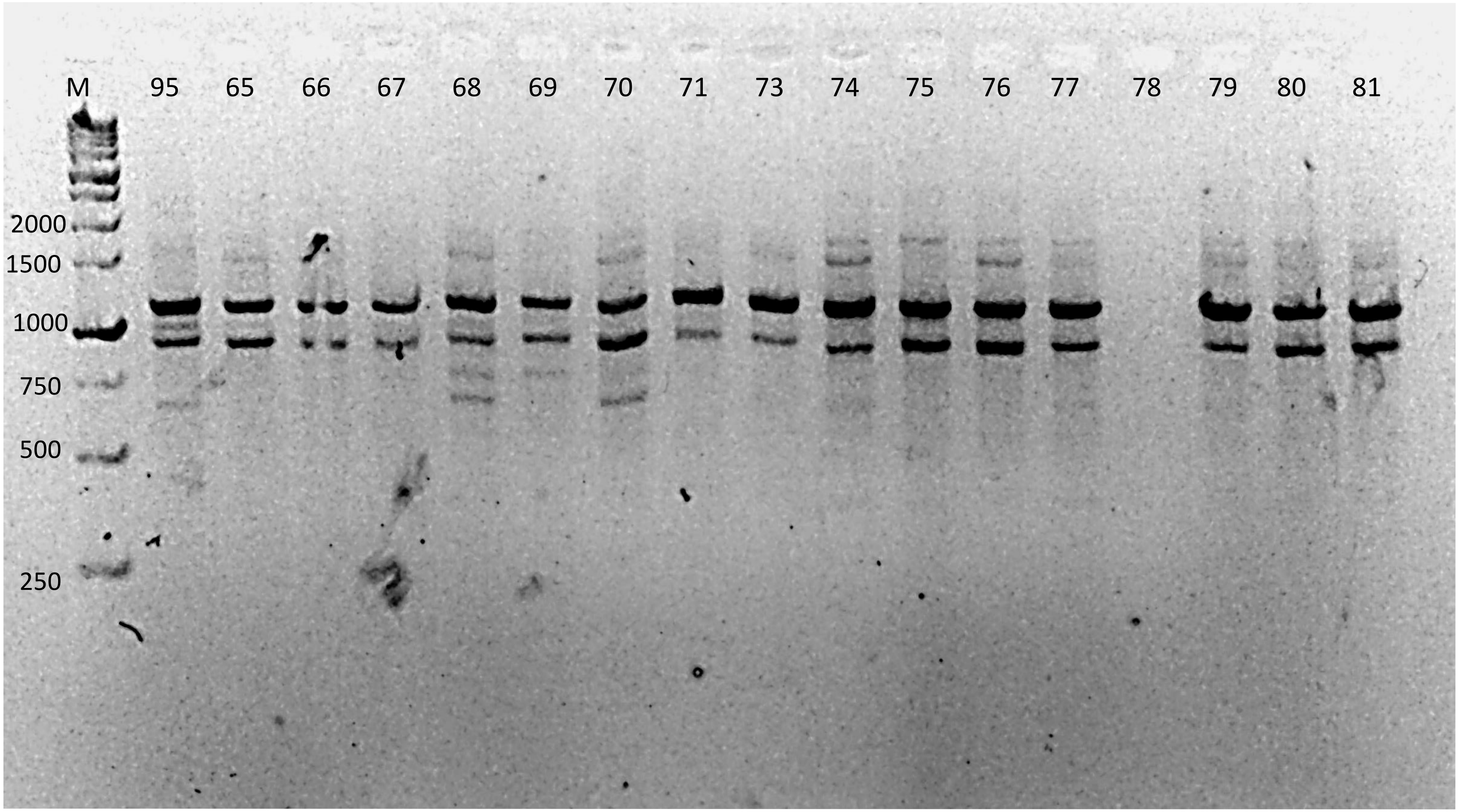
M
95
65
66
67
68
69
70
71
73
74
75
76
77
78
79
80
81
2000
1500
1000
750
500
250

SCoT34_82-94

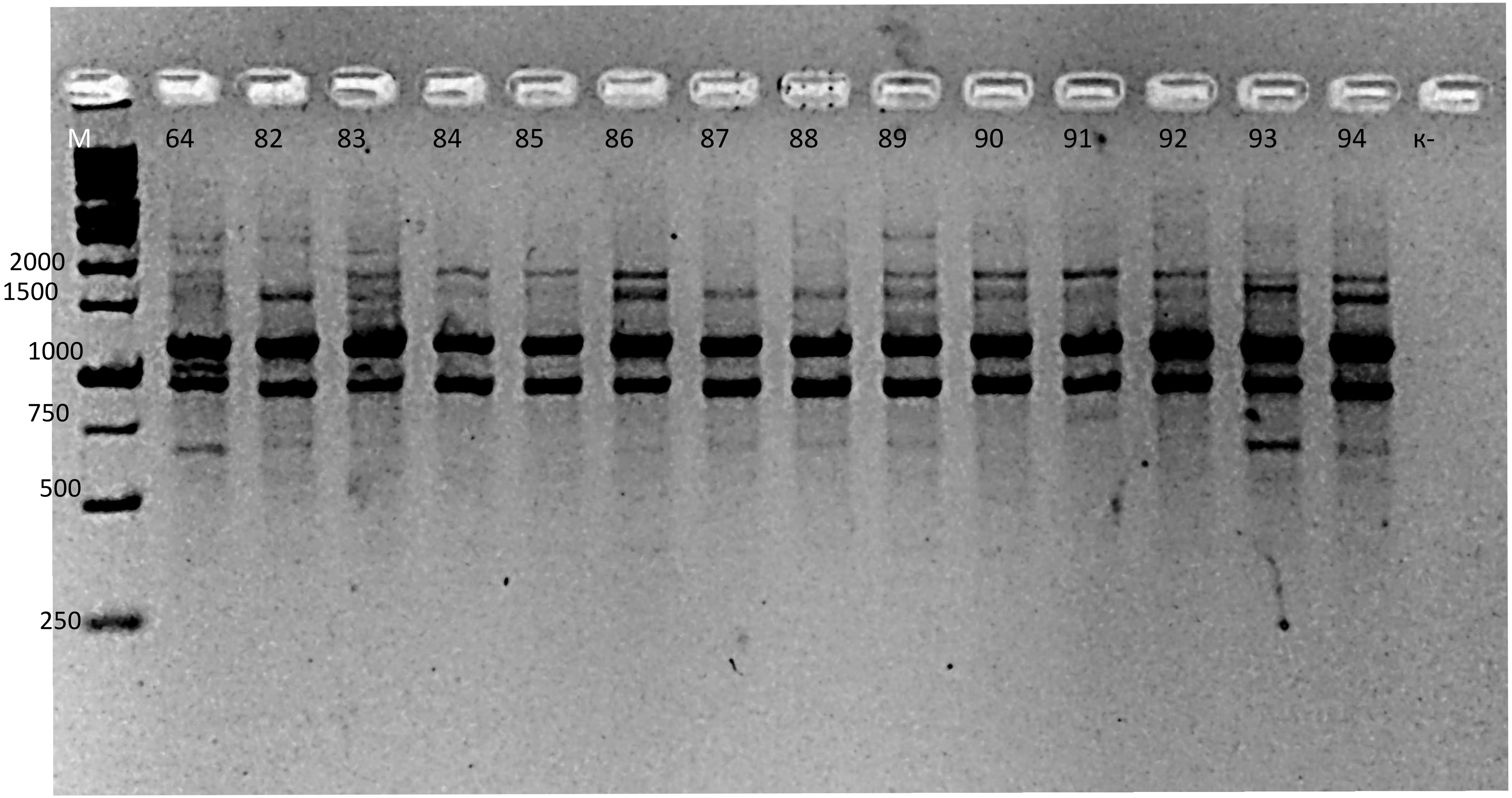
M
64
82
83
84
85
86
87
88
89
90
91
92
93
94
к-
2000
1500
1000
750
500
250

SCoT12_1-16

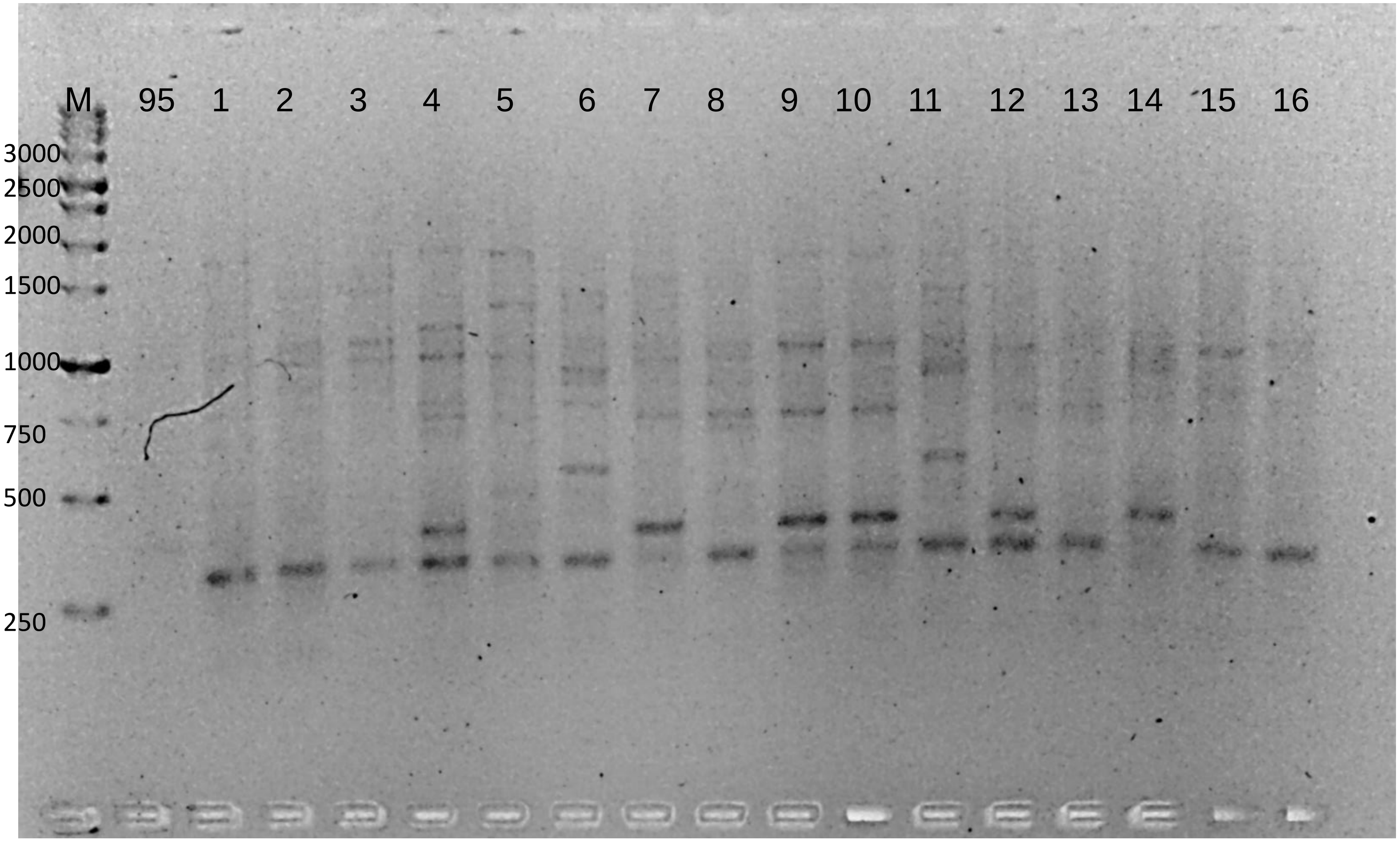
M 95 1 2 3 4 5 6 7 8 9 10 11 12 13 14 15 16
3000
2500
2000
1500
1000
750
500
250

SCoT12_17-32

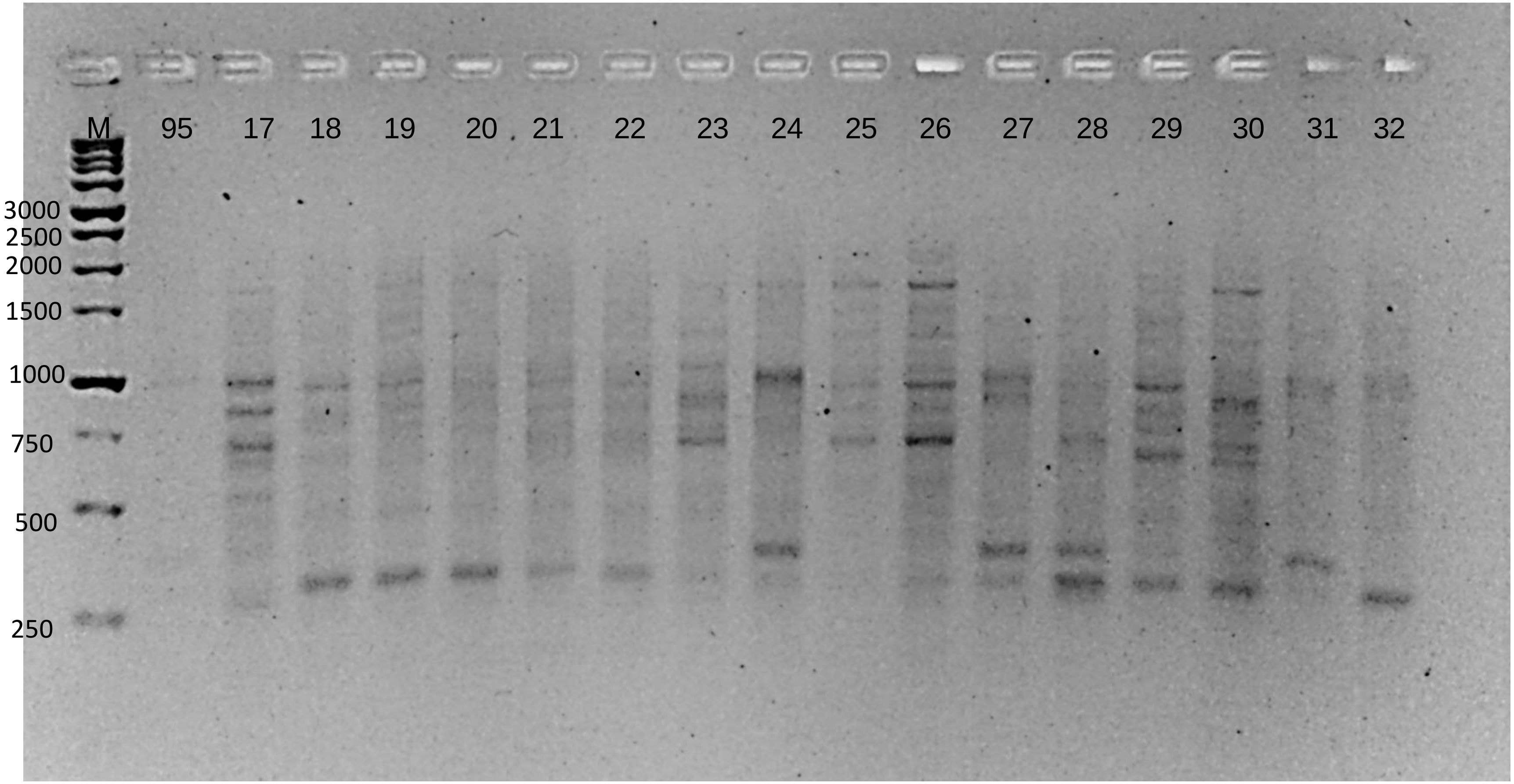
M
95
17
18
19
20
21
22
23
24
25
26
27
28
29
30
31
32
3000
2500
2000
1500
1000
750
500
250

SCoT12_33-48

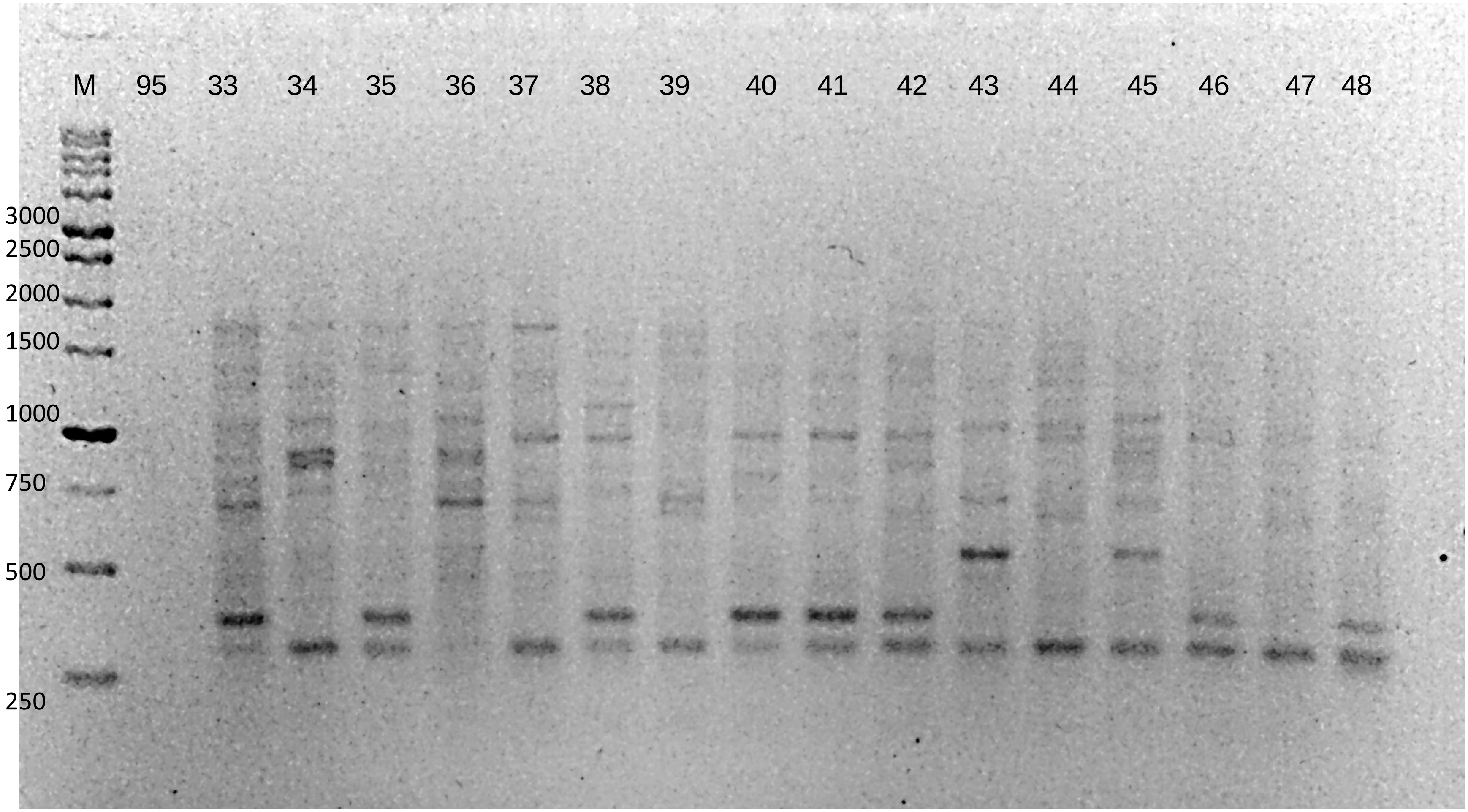
M 95 33 34 35 36 37 38 39 40 41 42 43 44 45 46 47 48
3000
2500
2000
1500
1000
750
500
250

SCoT12_49-64

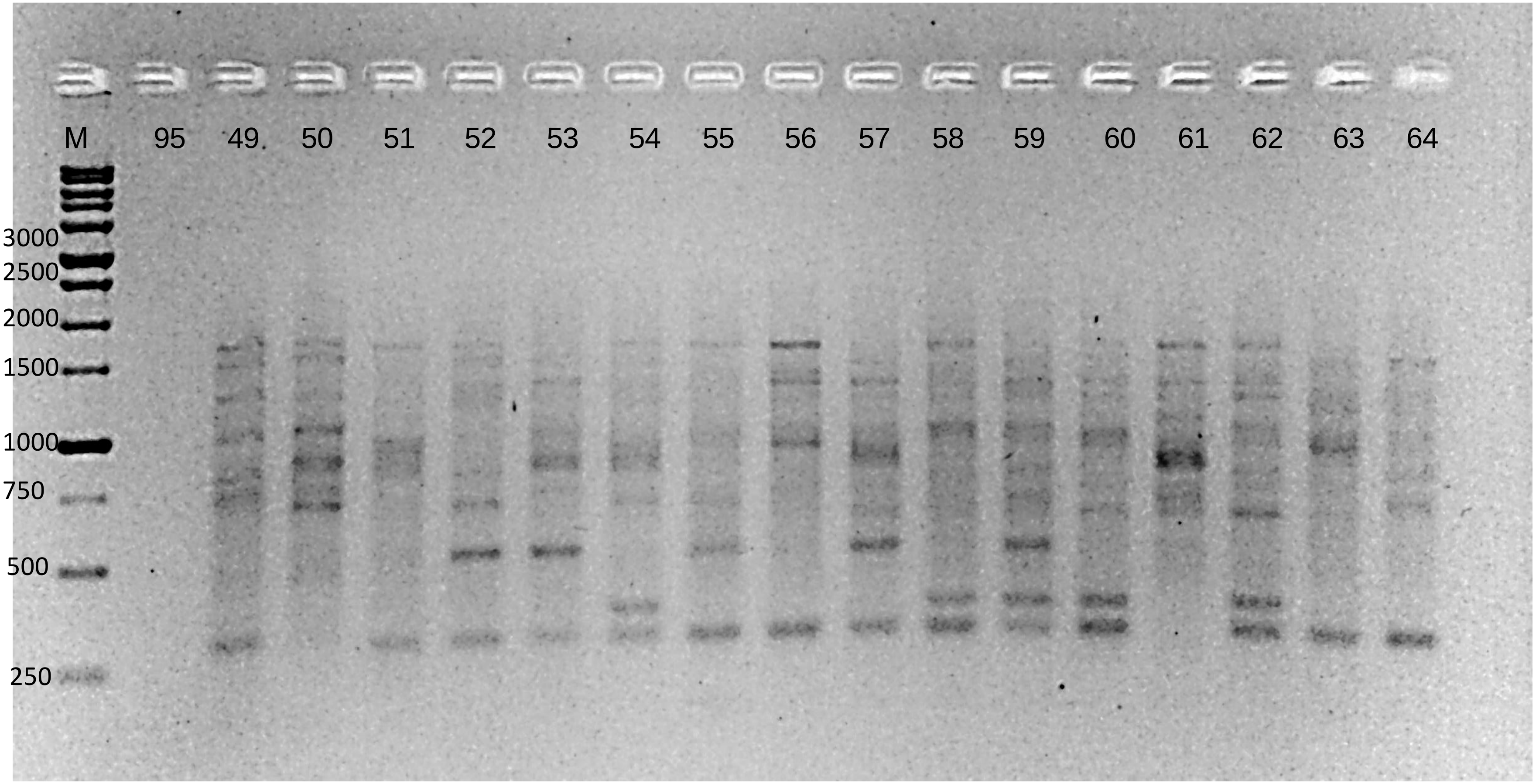
M
95
49
50
51
52
53
54
55
56
57
58
59
60
61
62
63
64
3000
2500
2000
1500
1000
750
500
250

SCoT12_65-81

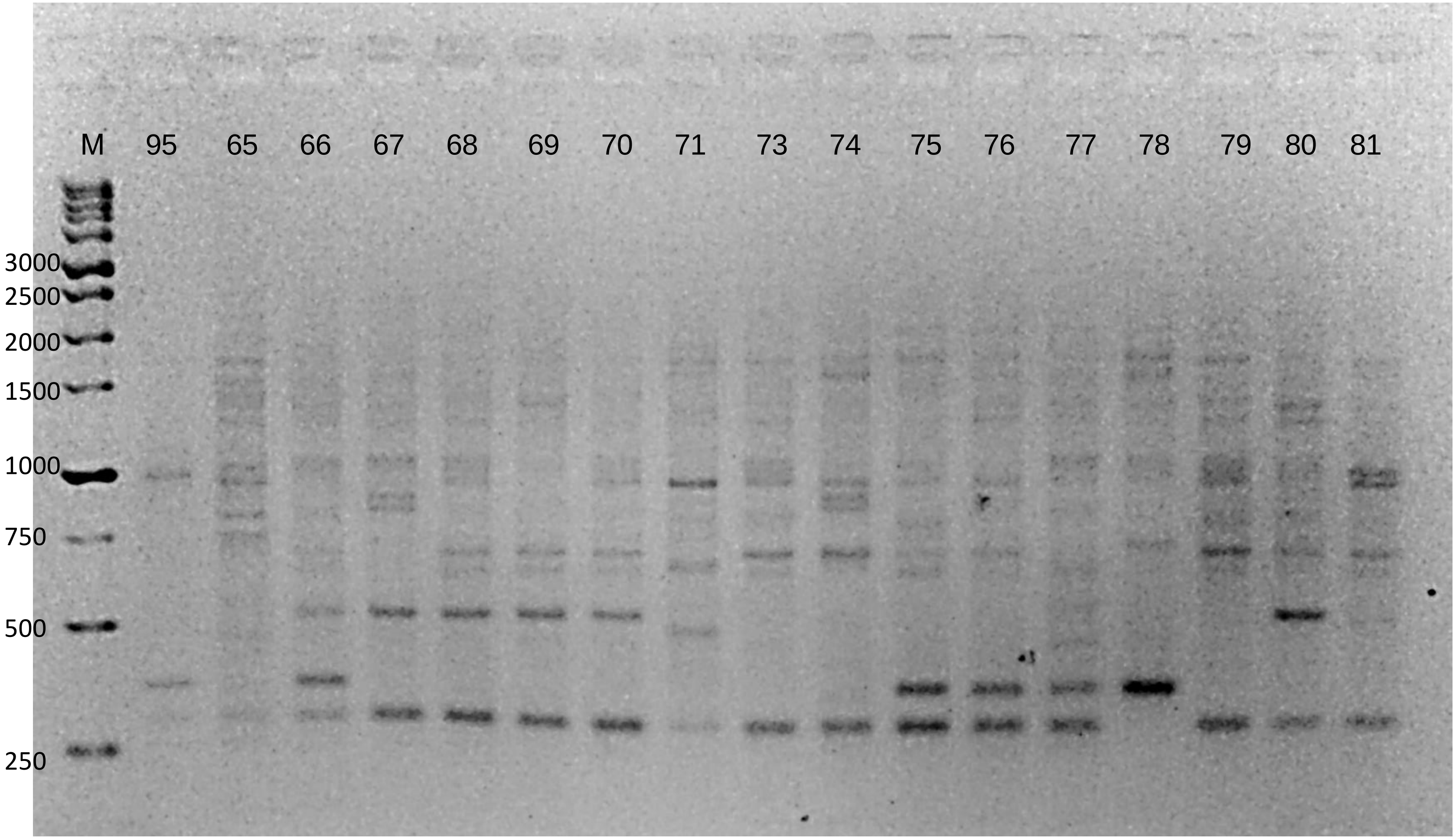
M 95 65 66 67 68 69 70 71 73 74 75 76 77 78 79 80 81
3000
2500
2000
1500
1000
750
500
250

SCoT12_82-94

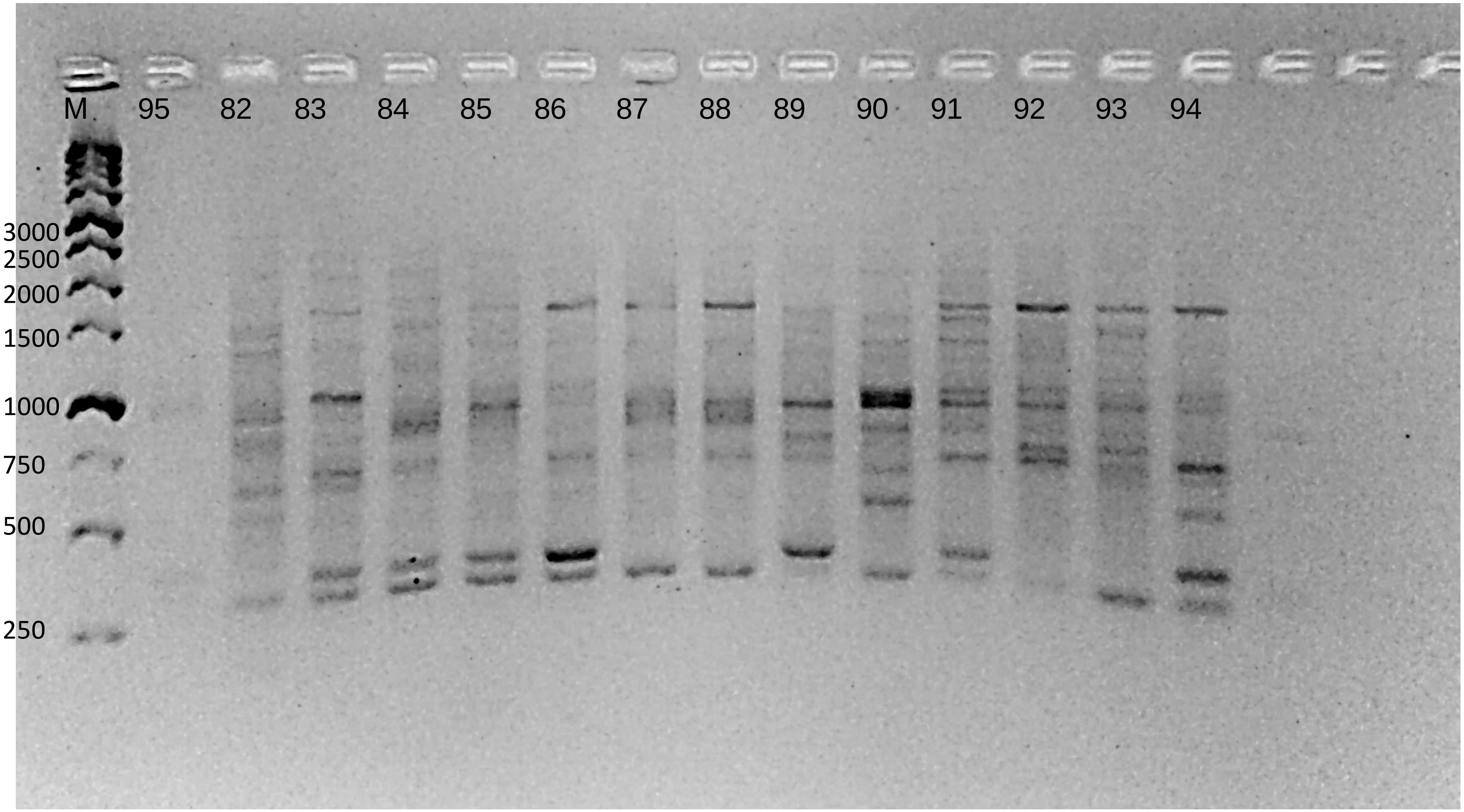
M
95
82
83
84
85
86
87
88
89
90
91
92
93
94
3000
2500
2000
1500
1000
750
500
250

ISSR873

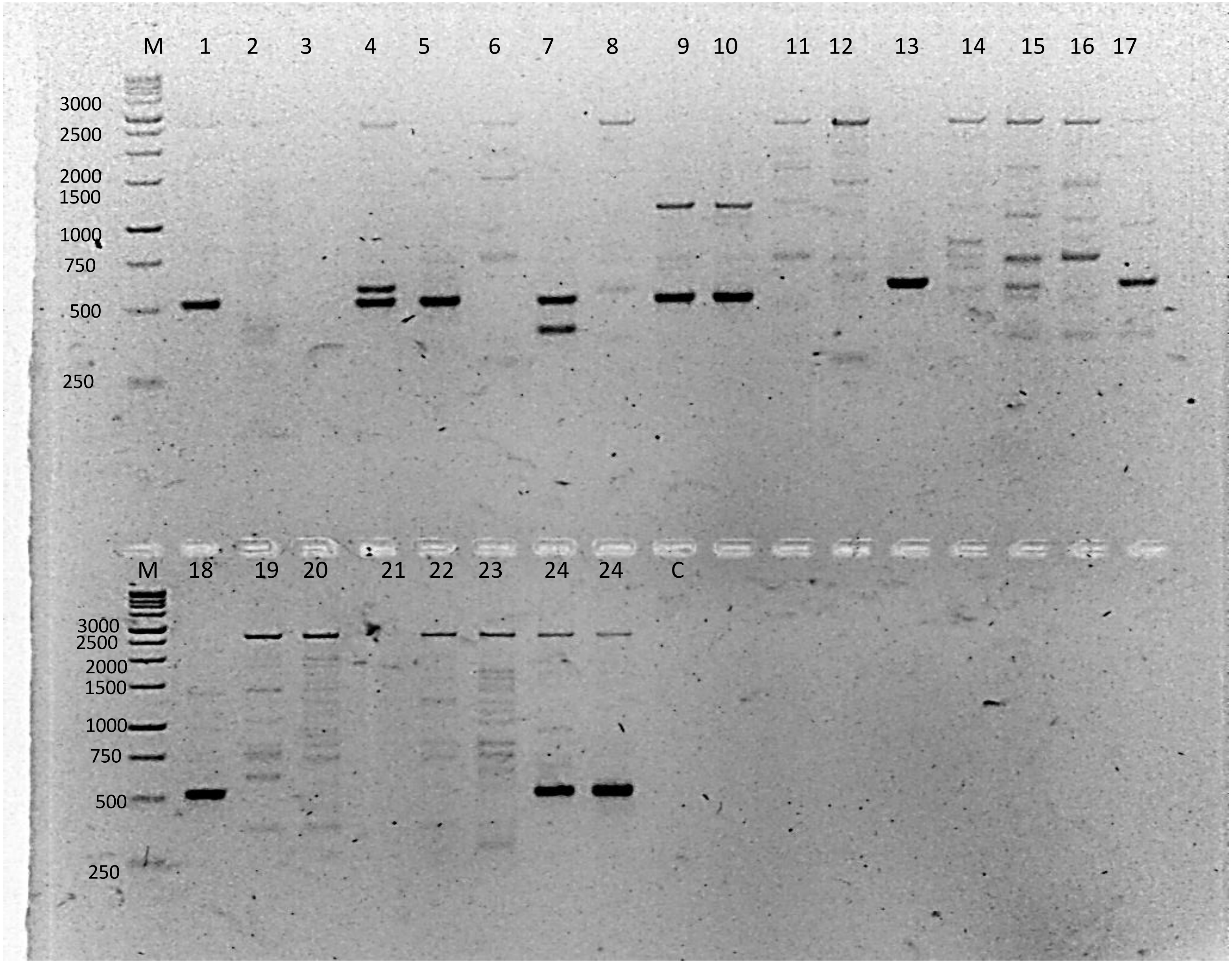
M 1 2 3 4 5 6 7 8 9 10 11 12 13 14 15 16 17
3000
2500
2000
1500
1000
750
500
250
M 18 19 20 21 22 23 24 24 C
3000
2500
2000
1500
1000
750
500
250

ISSR873

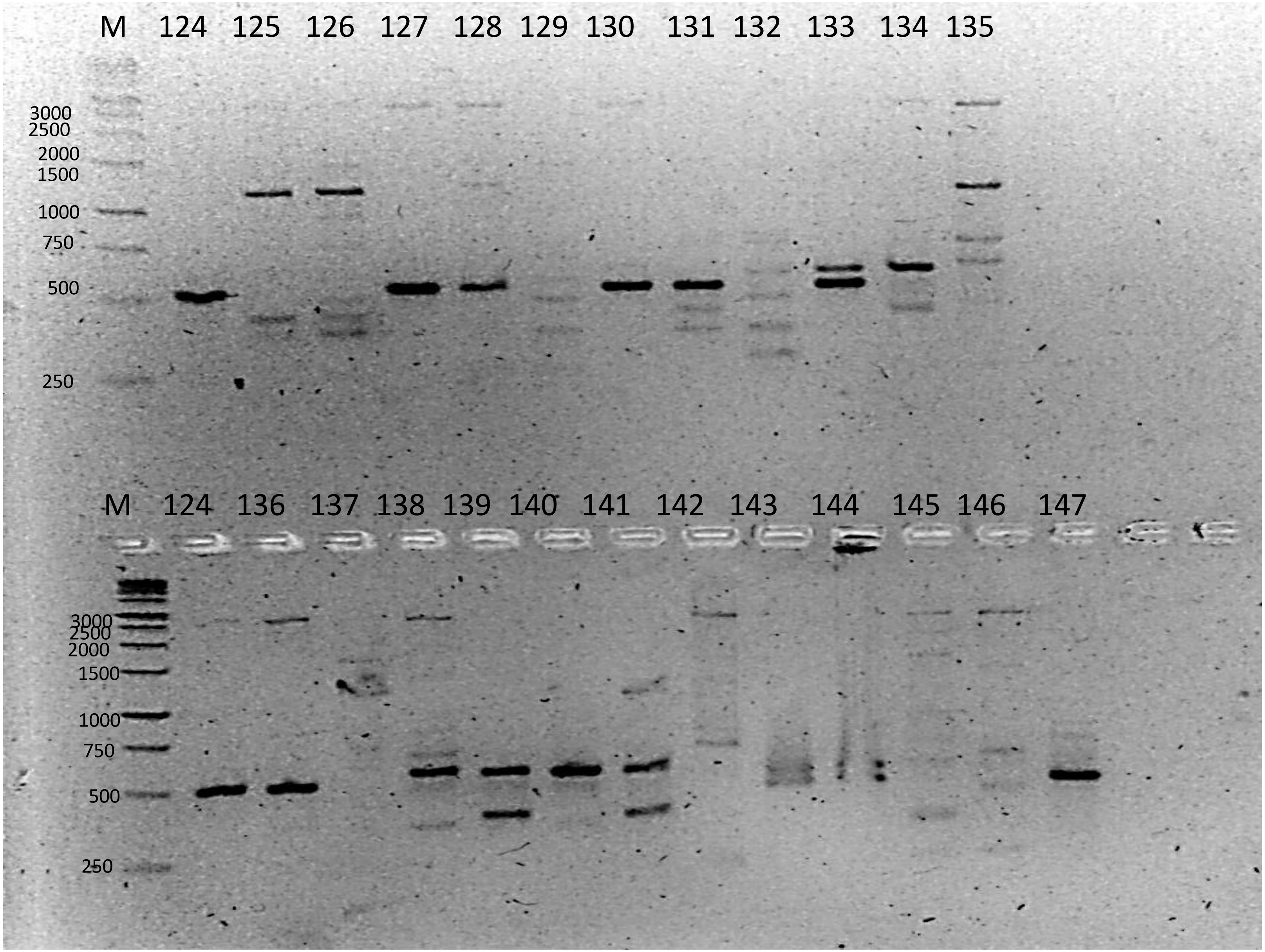

M 124 125 126 127 128 129 130 131 132 133 134 135
3000
2500
2000
1500
1000
750
500
250
M 124 136 137 138 139 140 141 142 143 144 145 146 147
3000
2500
2000
1500
1000
750
500
250

ISSR873

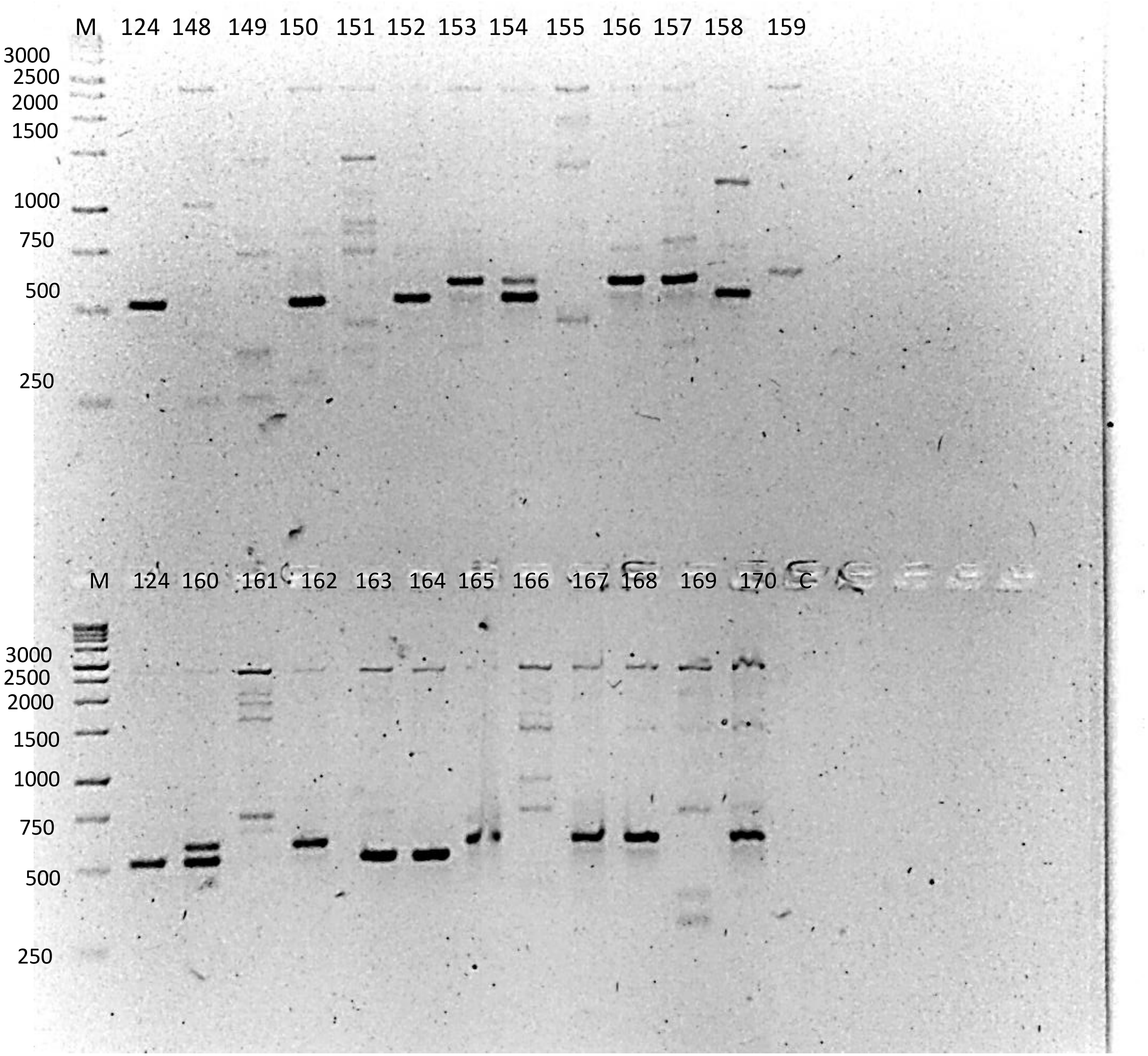

M 124 148 149 150 151 152 153 154 155 156 157 158 159
3000
2500
2000
1500
1000
750
500
250
M 124 160 161 162 163 164 165 166 167 168 169 170 C
3000
2500
2000
1500
1000
750
500
250

ISSR873

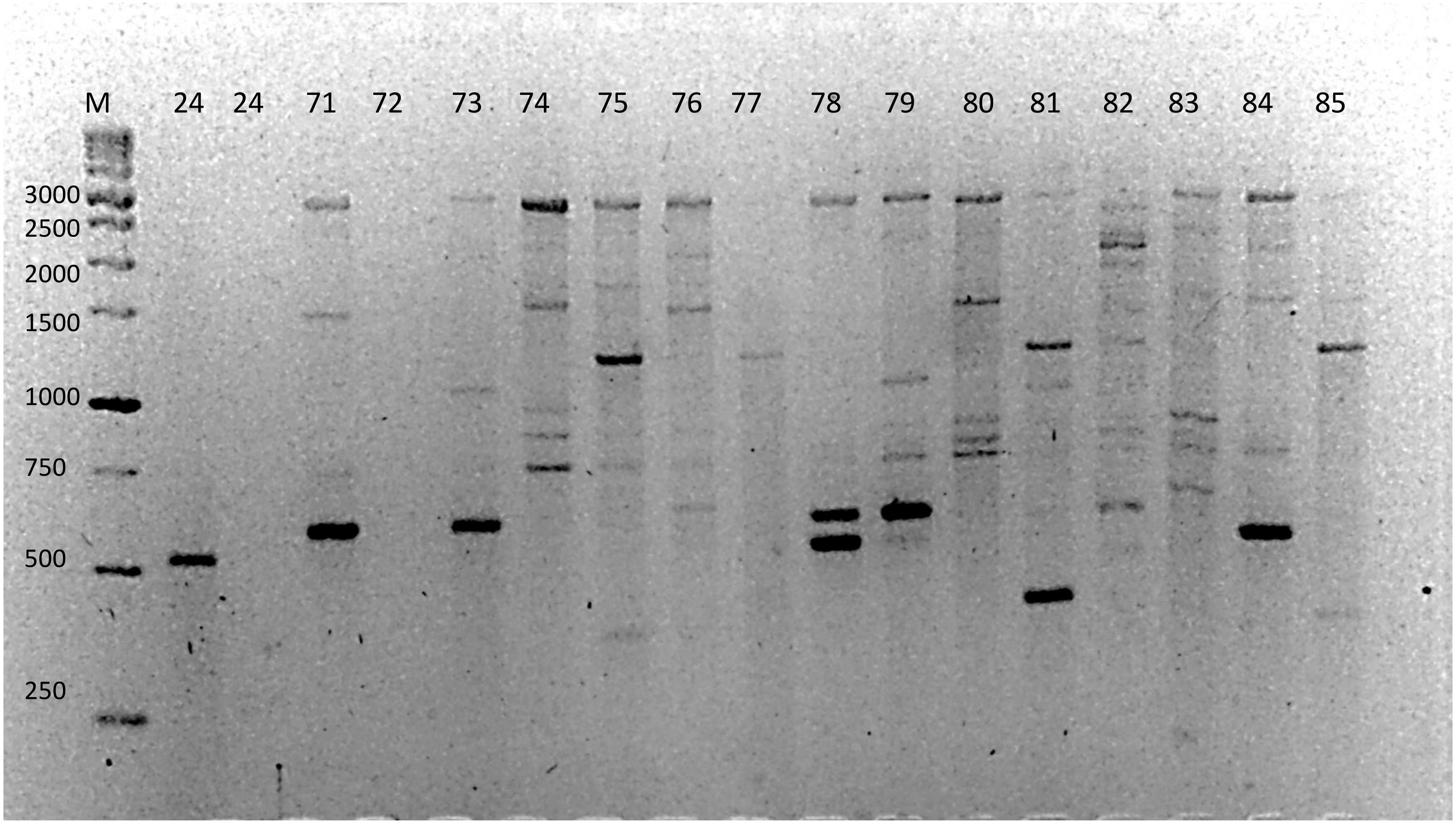
M
24
24
71
72
73
74
75
76
77
78
79
80
81
82
83
84
85
3000
2500
2000
1500
1000
750
500
250

ISSR873

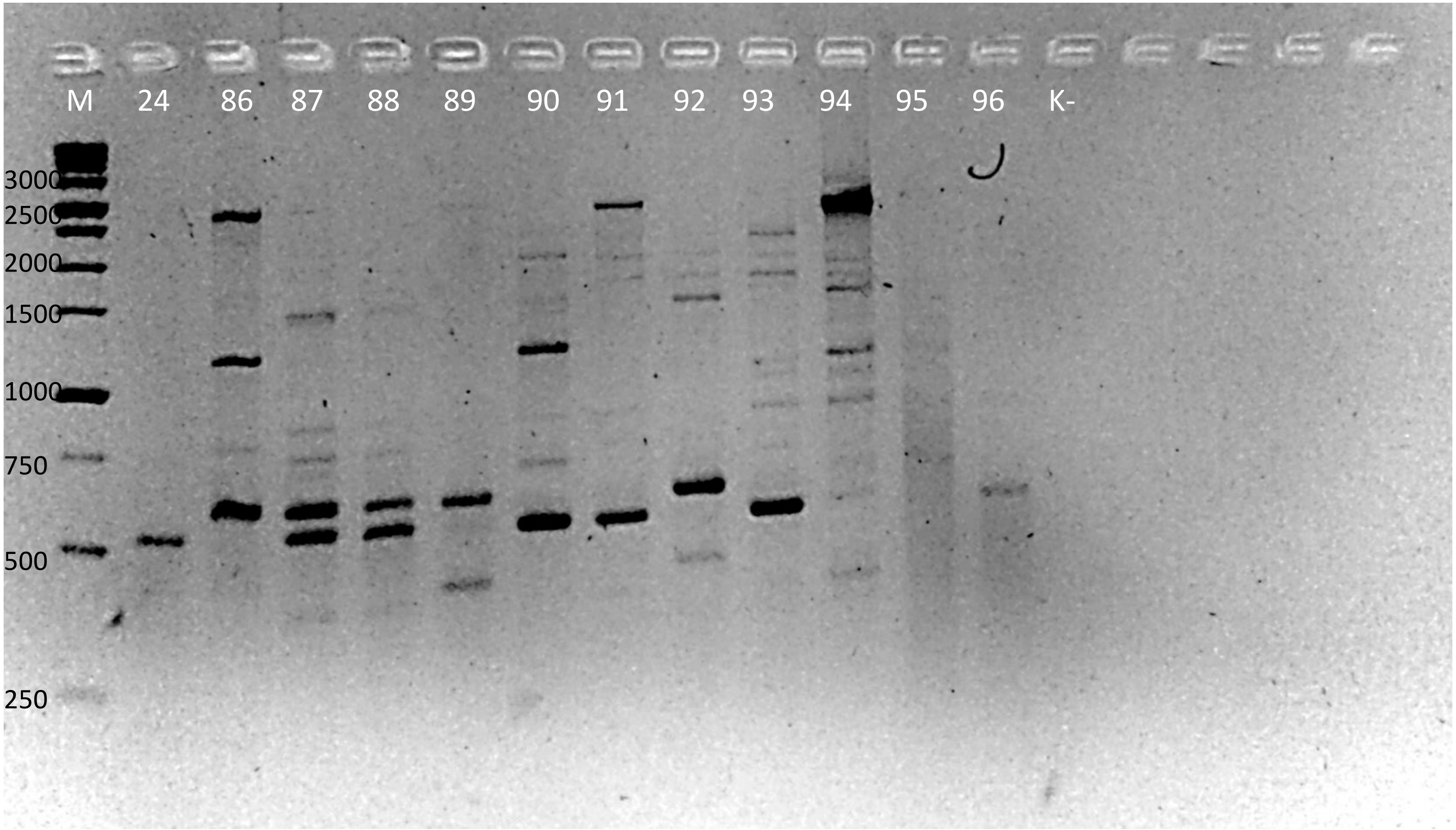

M
24
86
87
88
89
90
91
92
93
94
95
96
K-
3000
2500
2000
1500
1000
750
500
250

ISSR15

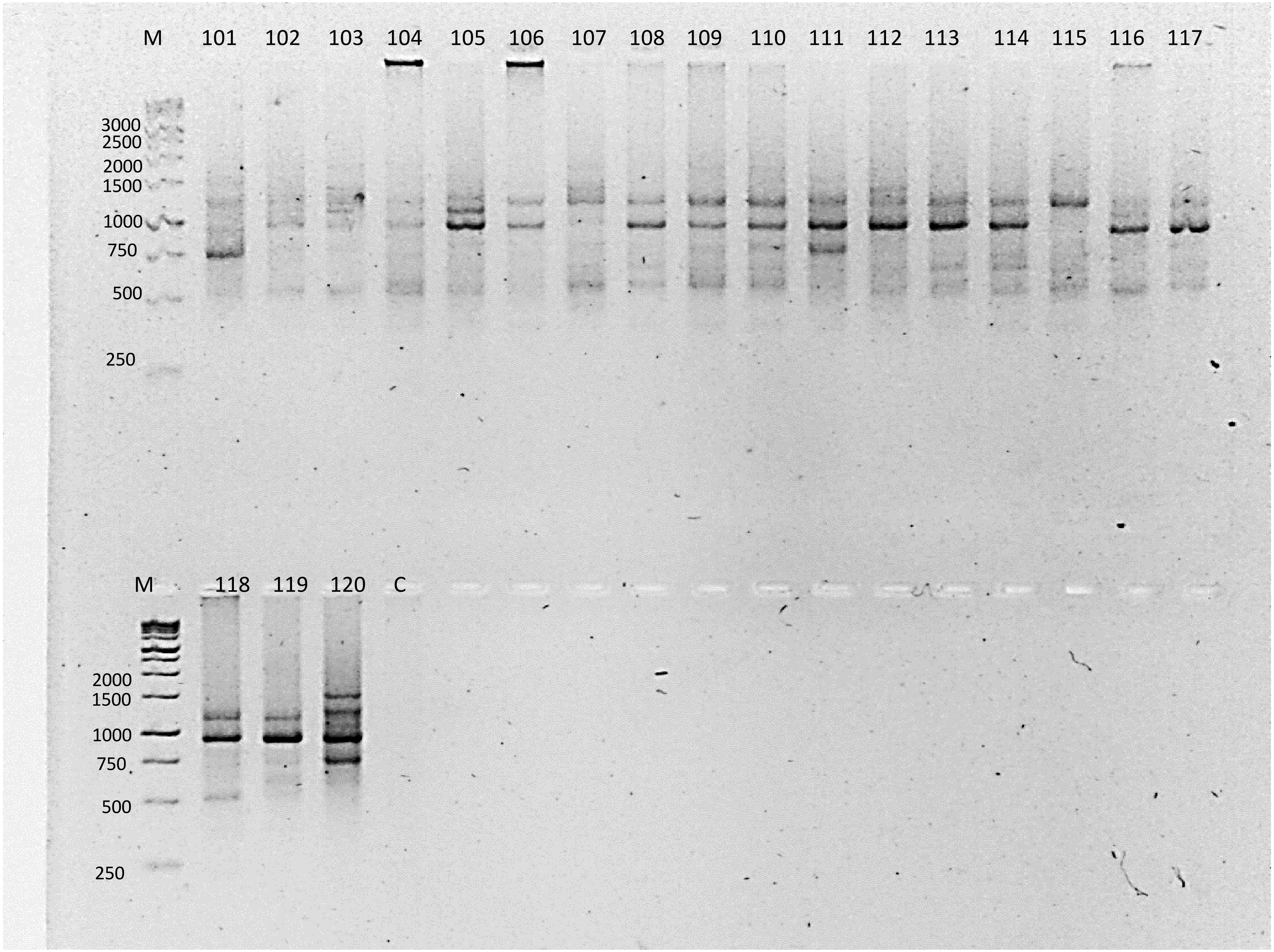
M
101
102
103
104
105
106
107
108
109
110
111
112
113
114
115
116
117
3000
2500
2000
1500
1000
750
500
250
M
118
119
120
C
2000
1500
1000
750
500
250

ISSR15

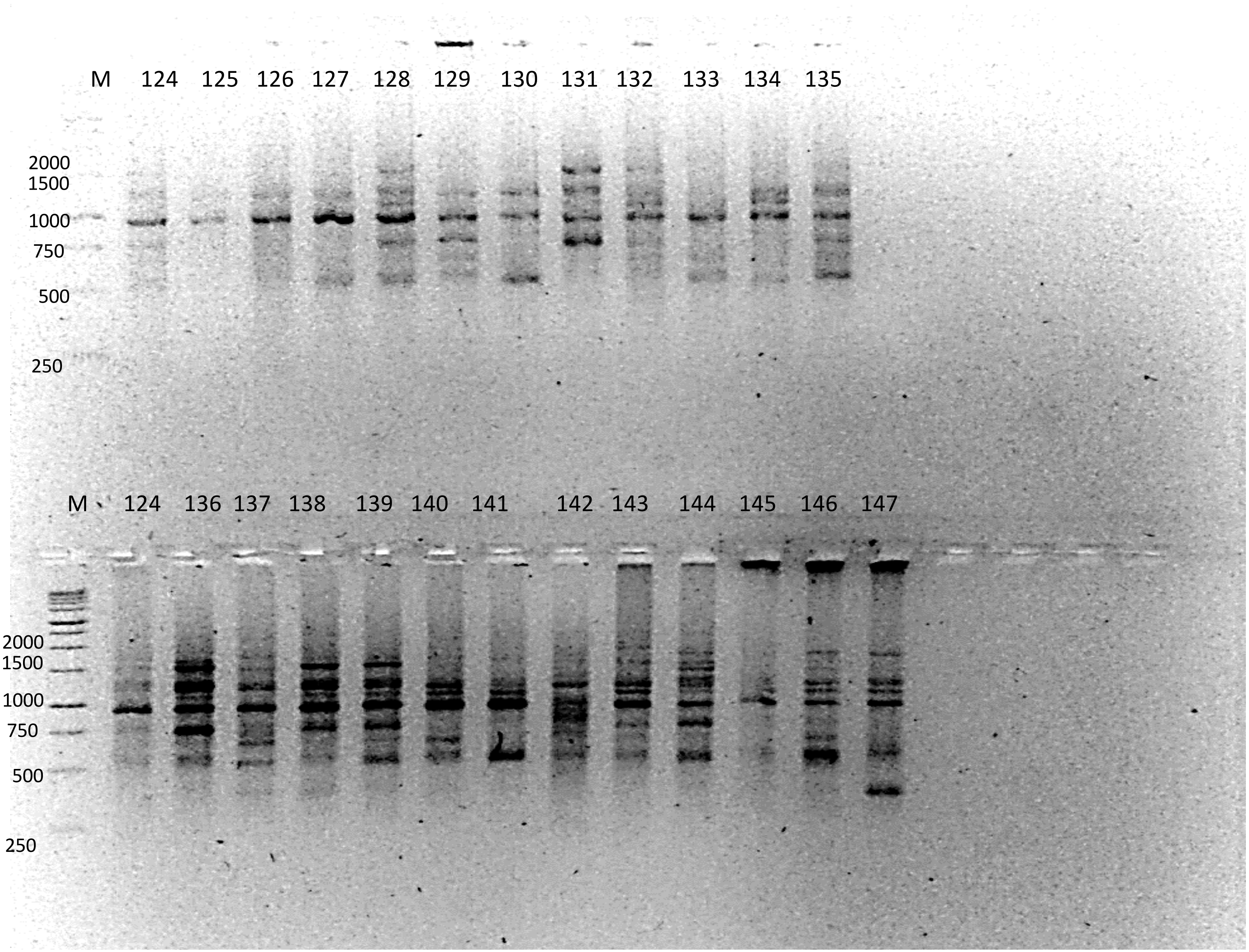
M 124 125 126 127 128 129 130 131 132 133 134 135
2000
1500
1000
750
500
250
M 124 136 137 138 139 140 141 142 143 144 145 146 147
2000
1500
1000
750
500
250

ISSR15

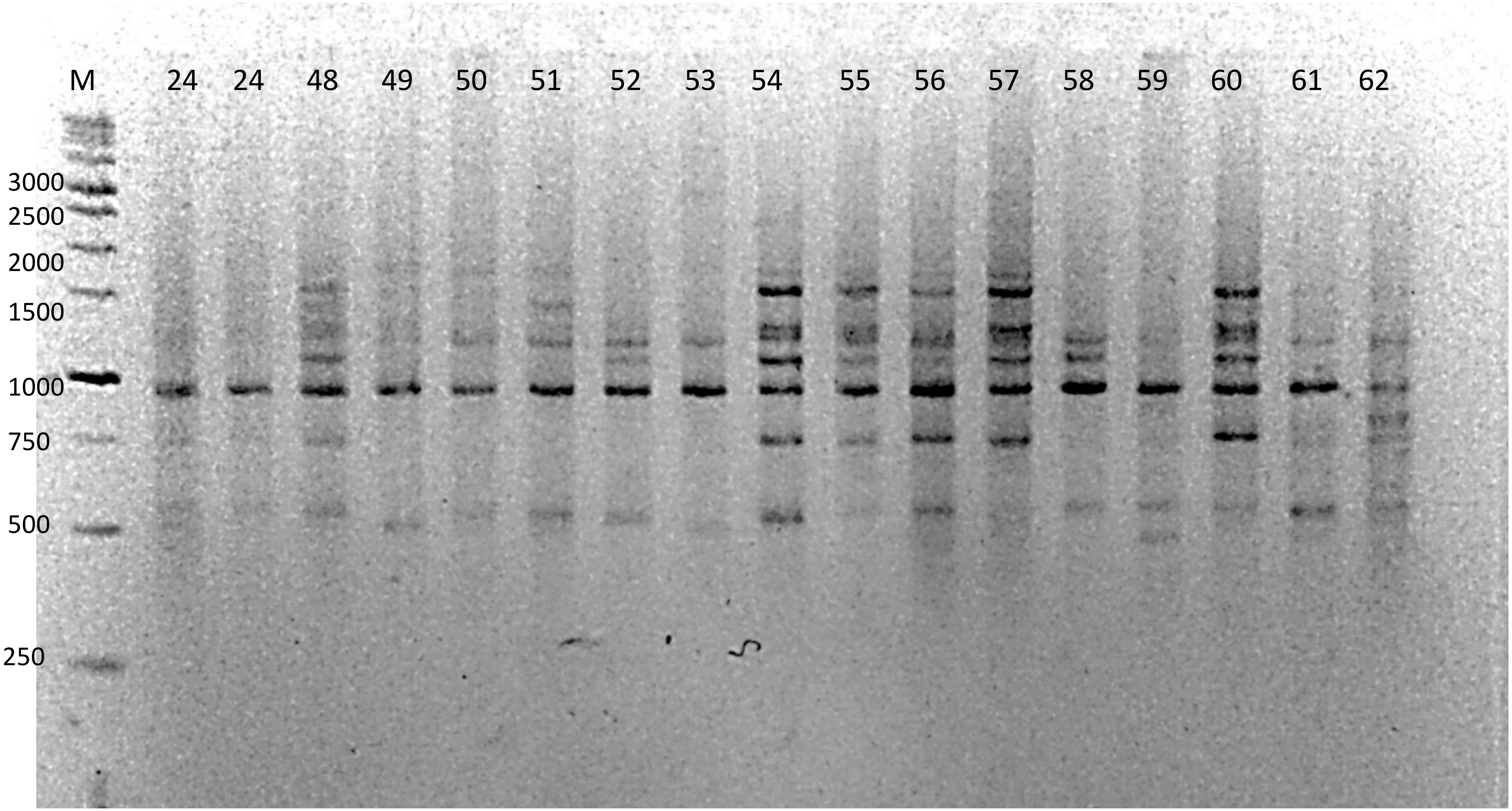

M
24
24
48
49
50
51
52
53
54
55
56
57
58
59
60
61
62
3000
2500
2000
1500
1000
750
500
250

ISSR15

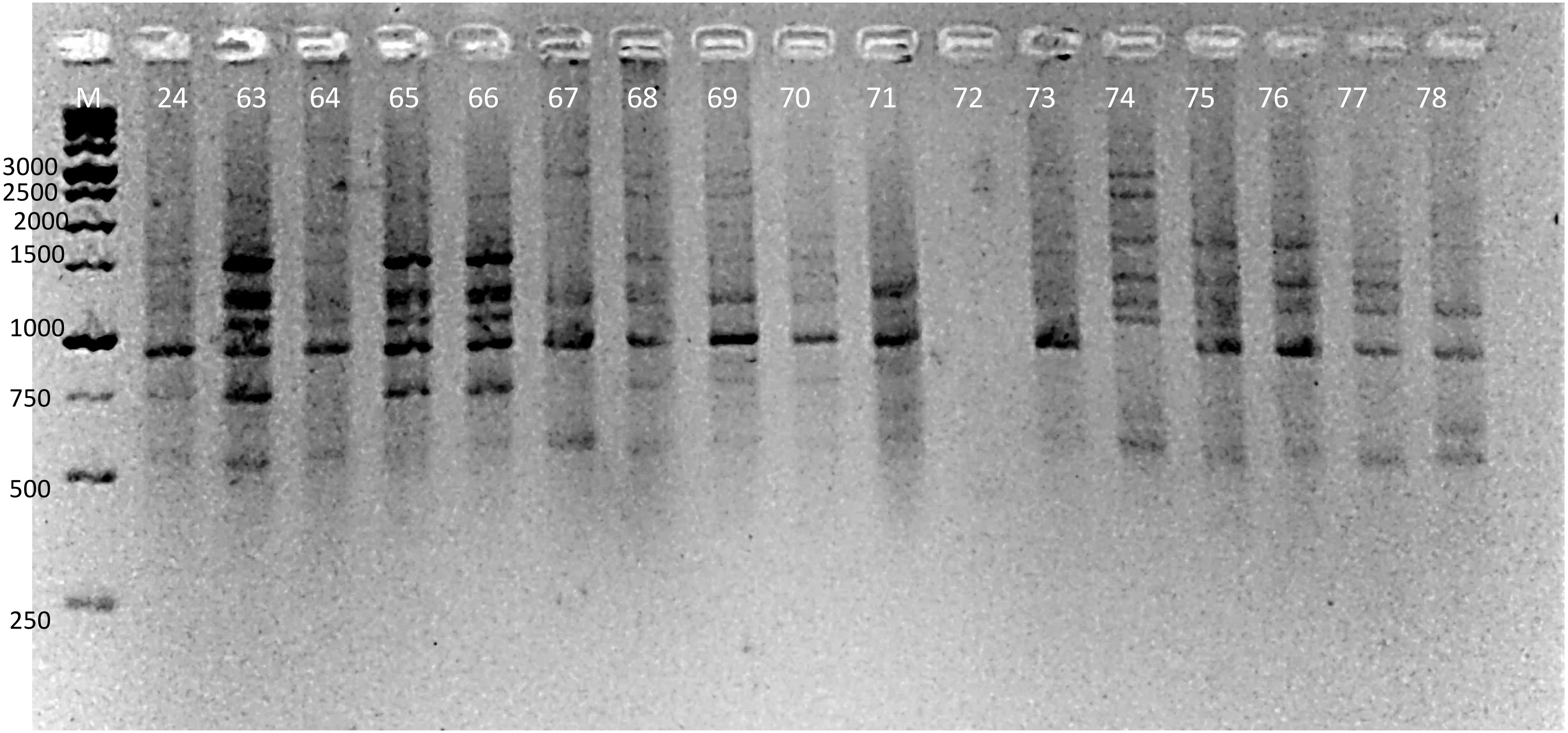
M
24
63
64
65
66
67
68
69
70
71
72
73
74
75
76
77
78
3000
2500
2000
1500
1000
750
500
250

ISSR15

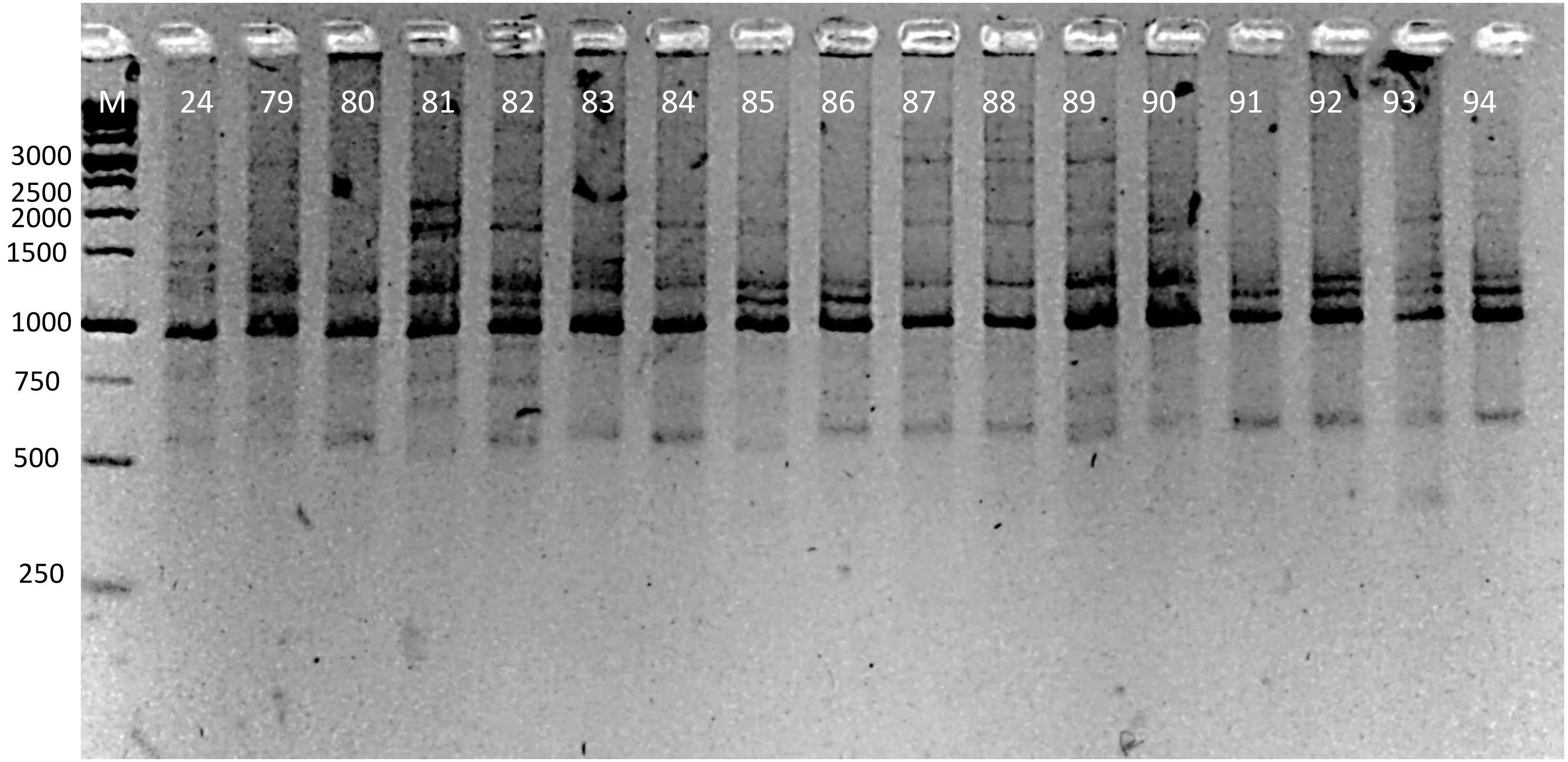
M
24
79
80
81
82
83
84
85
86
87
88
89
90
91
92
93
94
3000
2500
2000
1500
1000
750
500
250

ISSR880

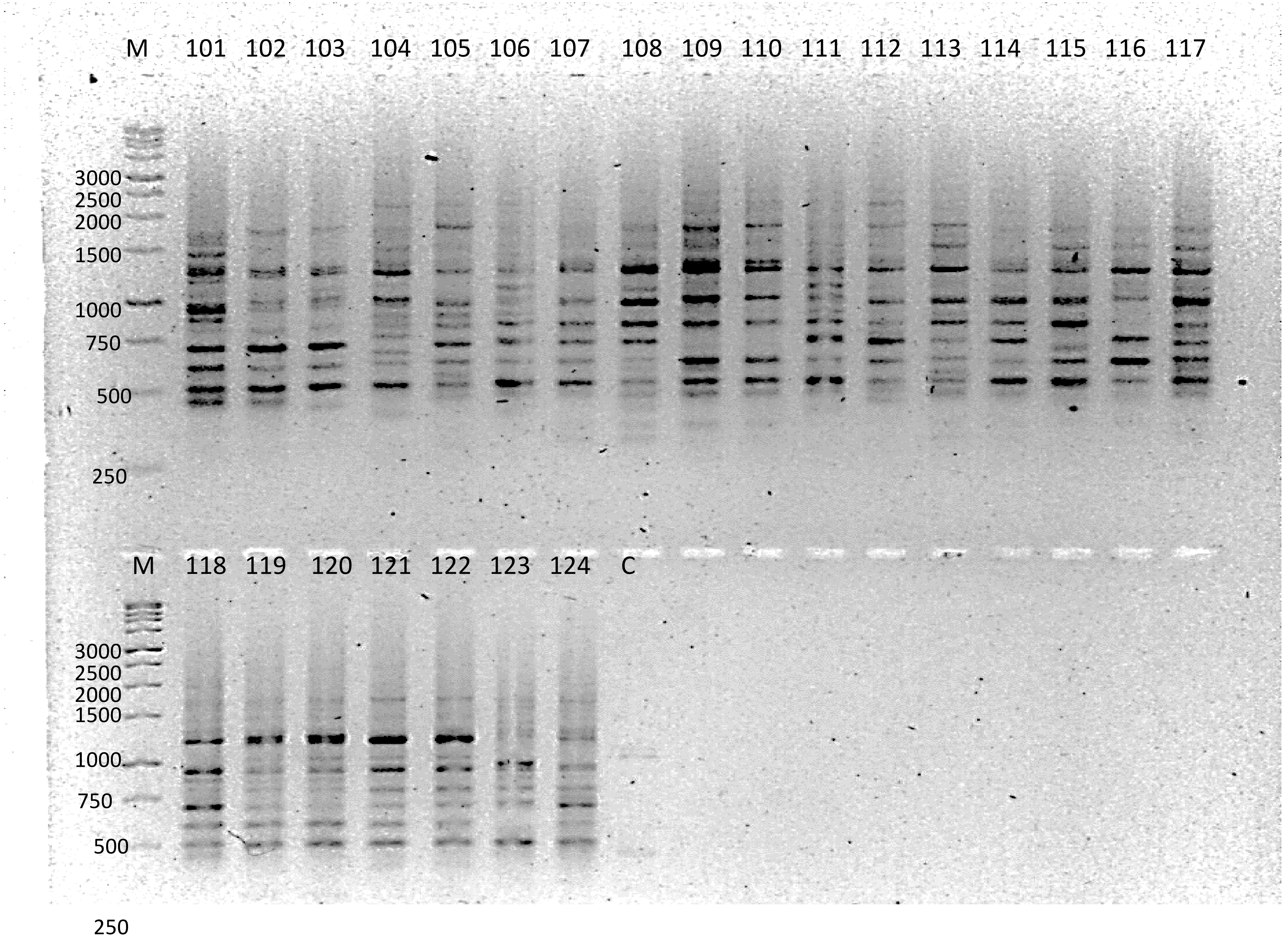
M 101 102 103 104 105 106 107 108 109 110 111 112 113 114 115 116 117
3000
2500
2000
1500
1000
750
500
250
M 118 119 120 121 122 123 124 C
3000
2500
2000
1500
1000
750
500
250

ISSR880

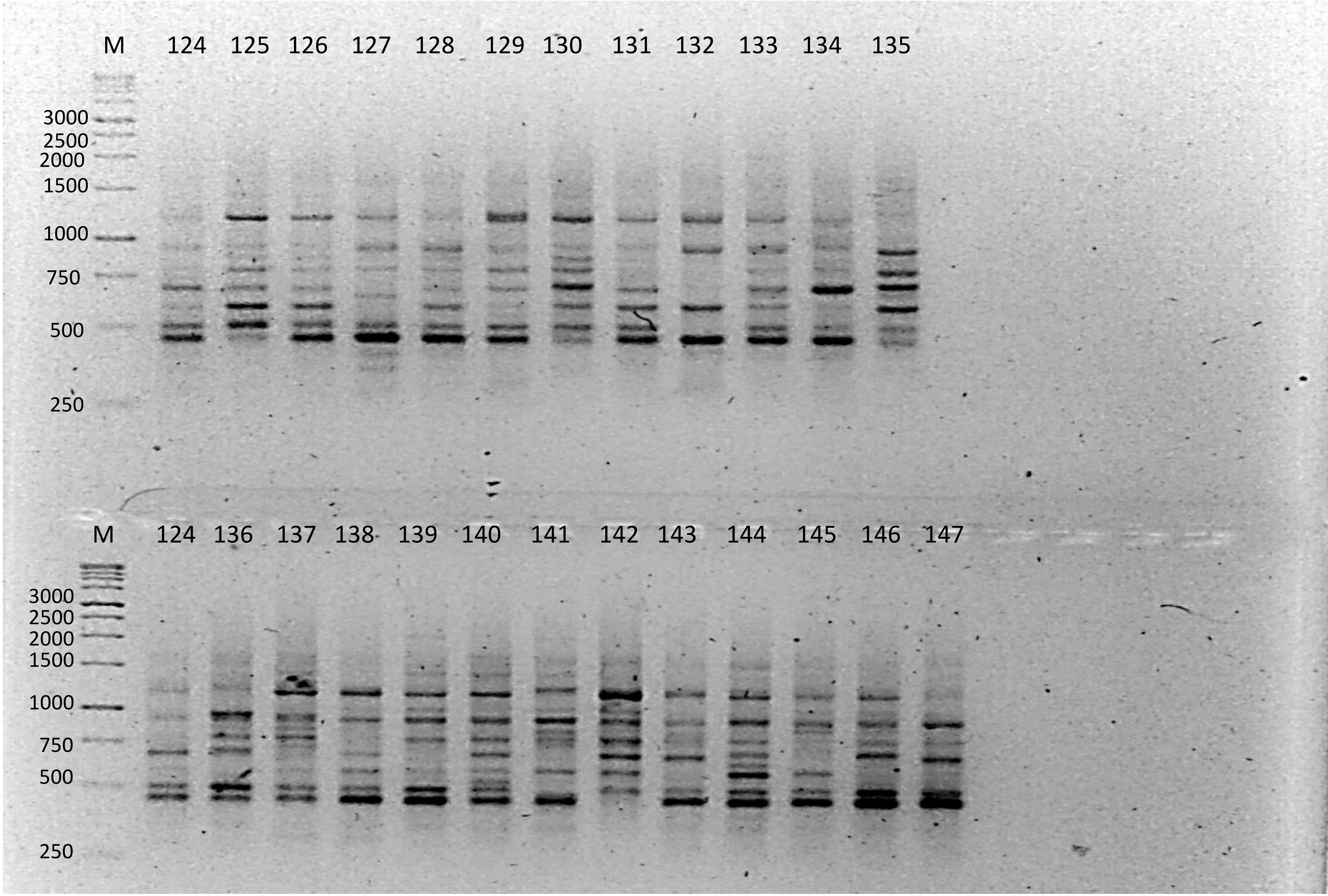
M 124 125 126 127 128 129 130 131 132 133 134 135
3000
2500
2000
1500
1000
750
500
250
M 124 136 137 138 139 140 141 142 143 144 145 146 147
3000
2500
2000
1500
1000
750
500
250

ISSR810

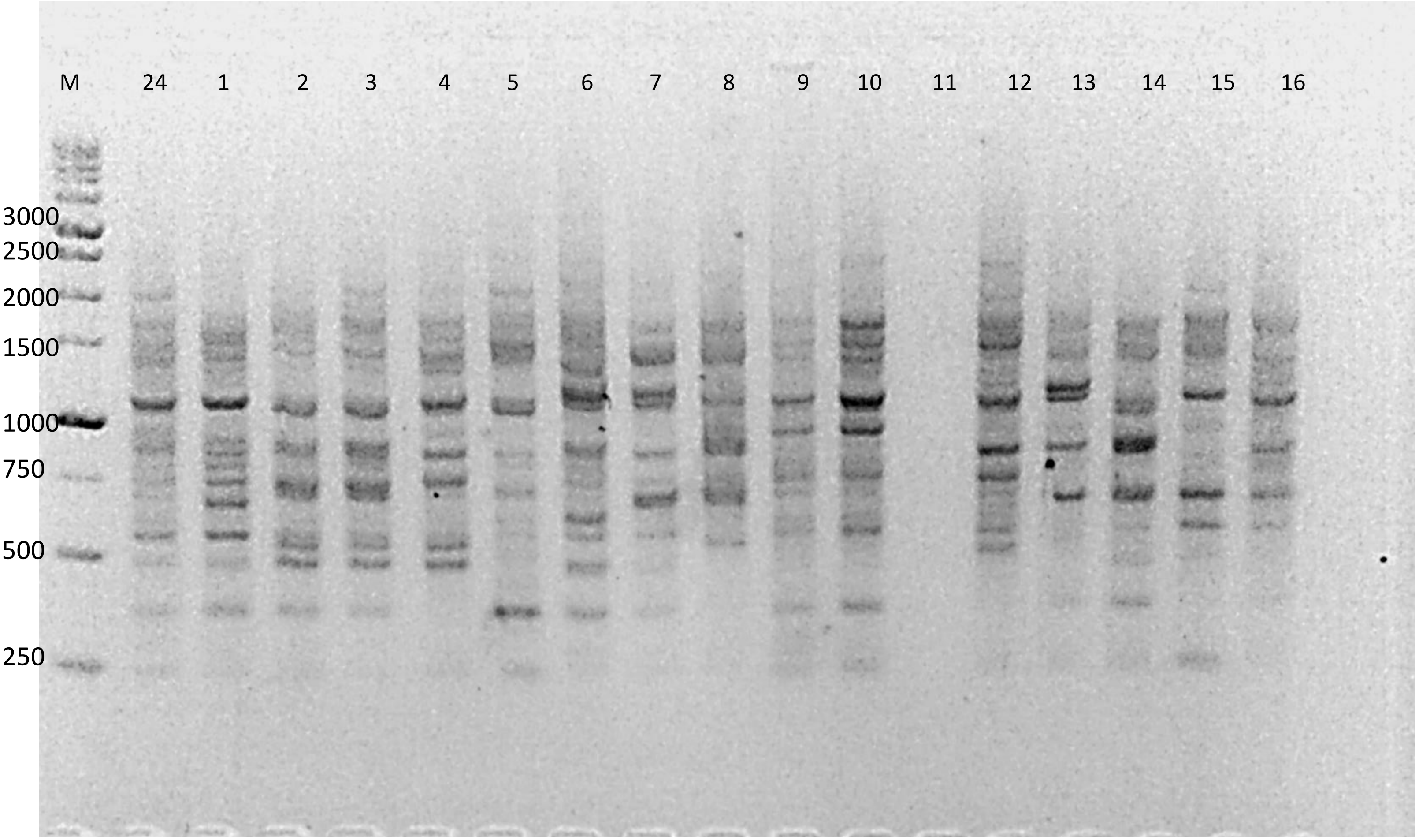
M 24 1 2 3 4 5 6 7 8 9 10 11 12 13 14 15 16
3000
2500
2000
1500
1000
750
500
250

ISSR810

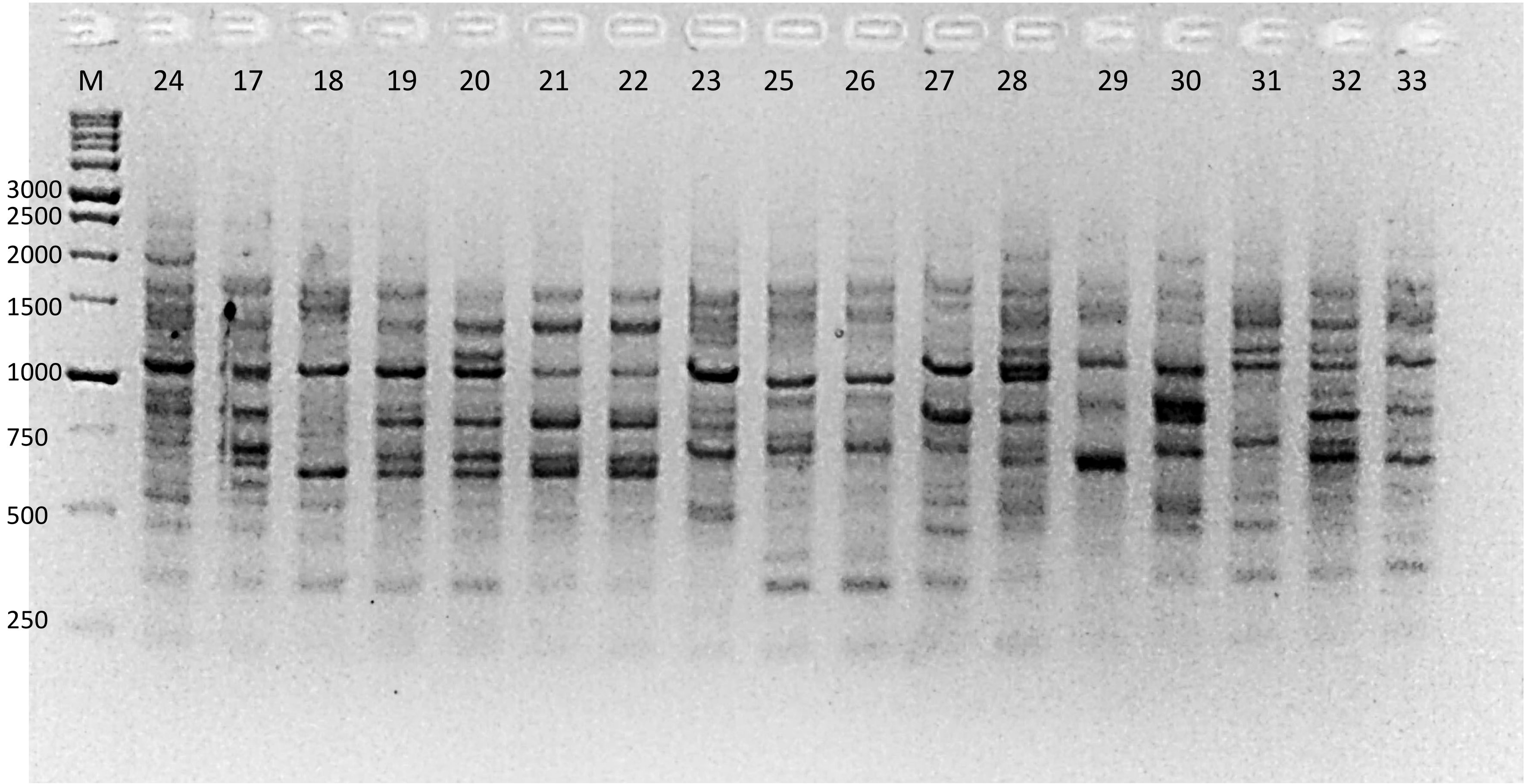
M 24 17 18 19 20 21 22 23 25 26 27 28 29 30 31 32 33
3000
2500
2000
1500
1000
750
500
250

ISSR810

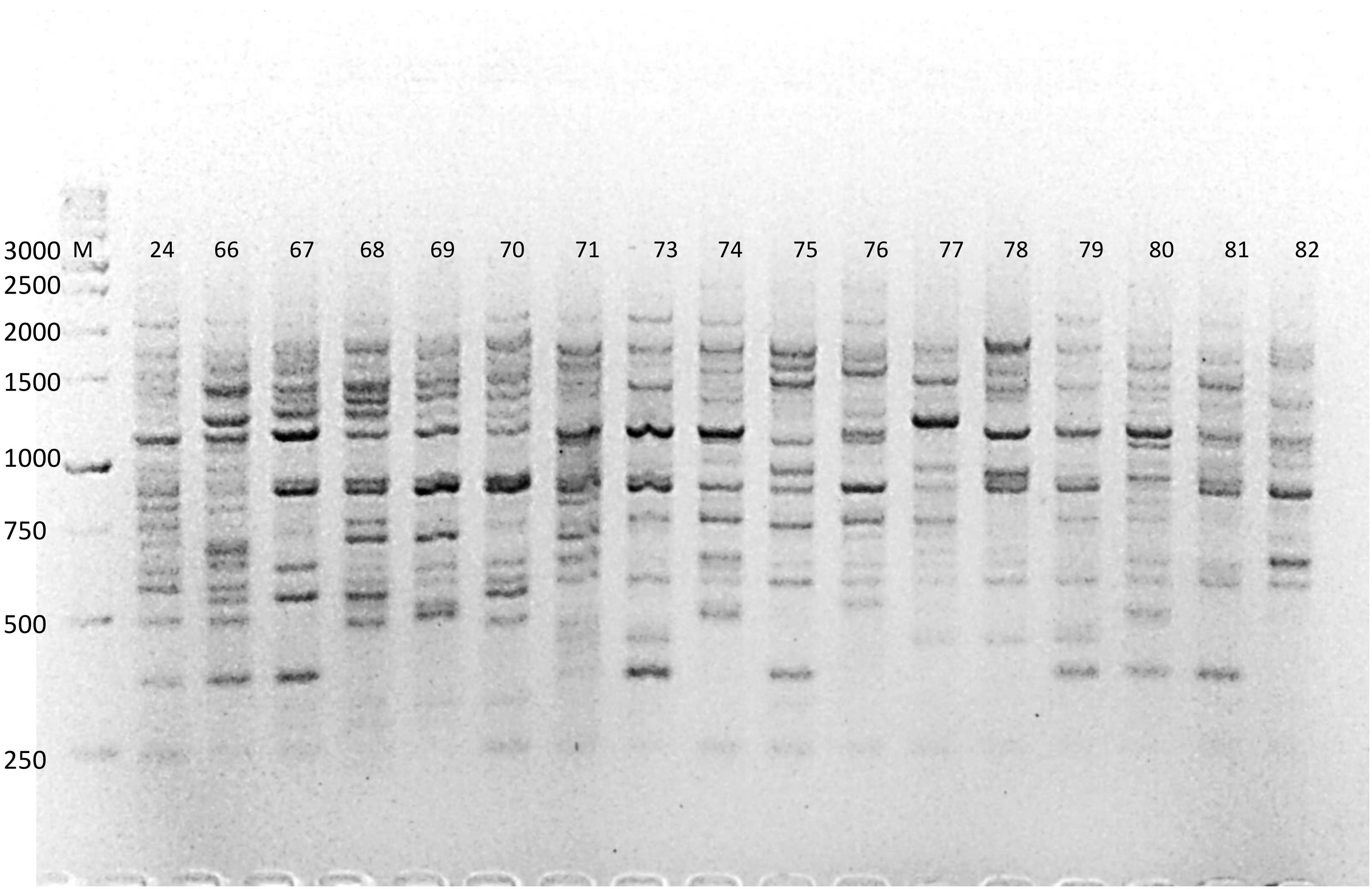

3000
2500
2000
1500
1000
750
500
250
M 24 66 67 68 69 70 71 73 74 75 76 77 78 79 80 81 82

ISSR814.1

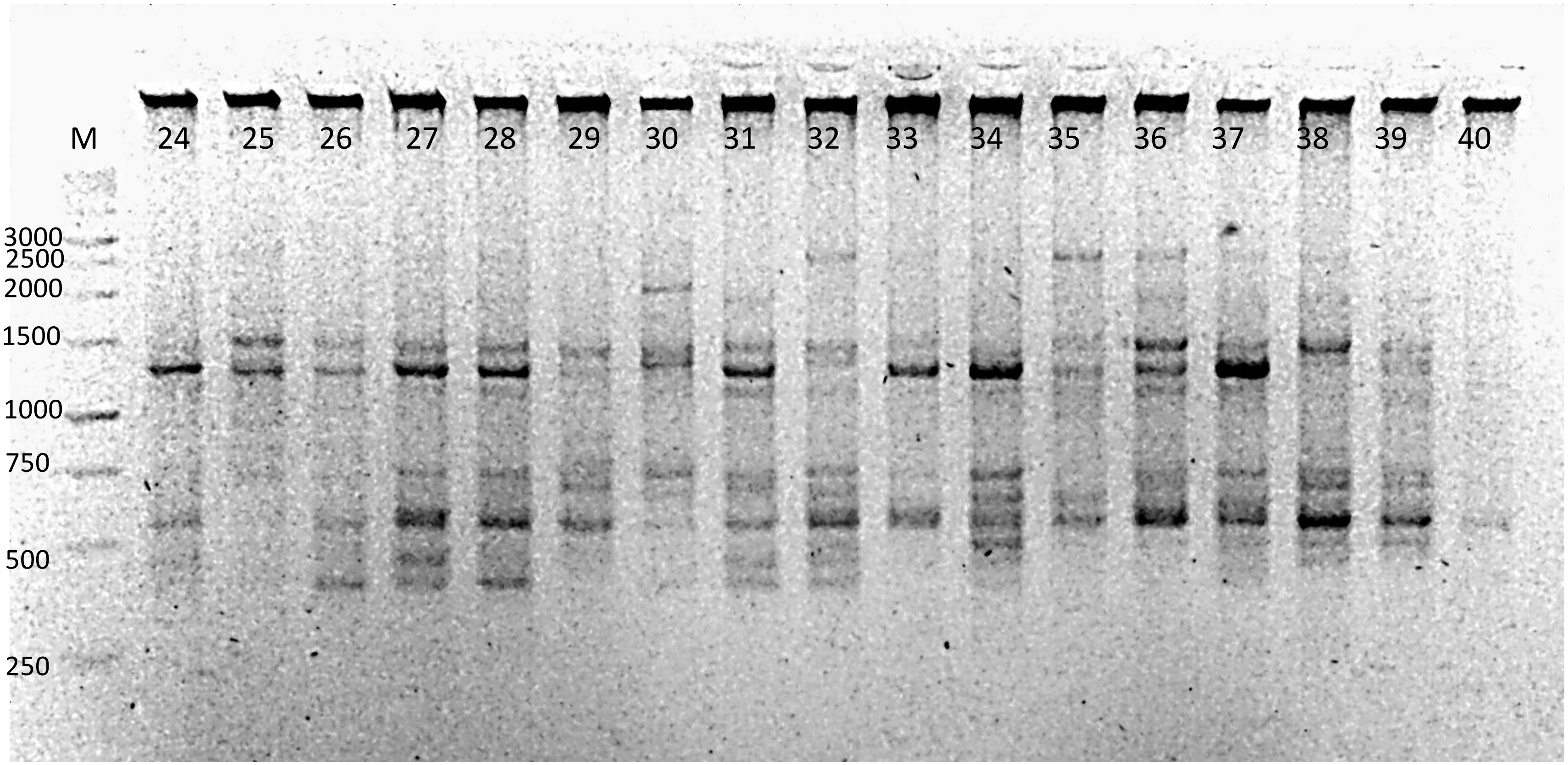
M
24
25
26
27
28
29
30
31
32
33
34
35
36
37
38
39
40
3000
2500
2000
1500
1000
750
500
250

ISSR814.1

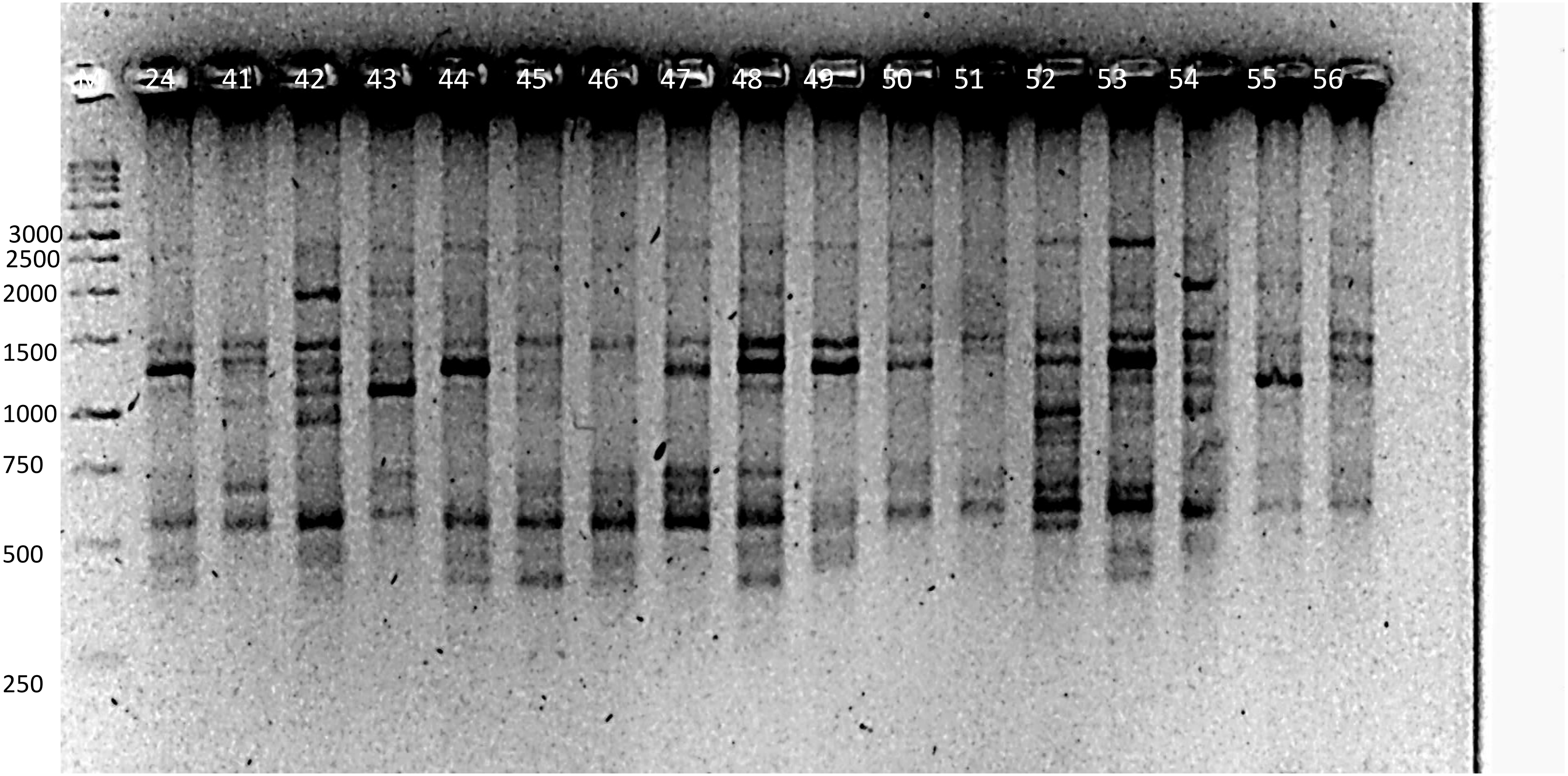
M
24
41
42
43
44
45
46
47
48
49
50
51
52
53
54
55
56
3000
2500
2000
1500
1000
750
500
250

ISSR814.1

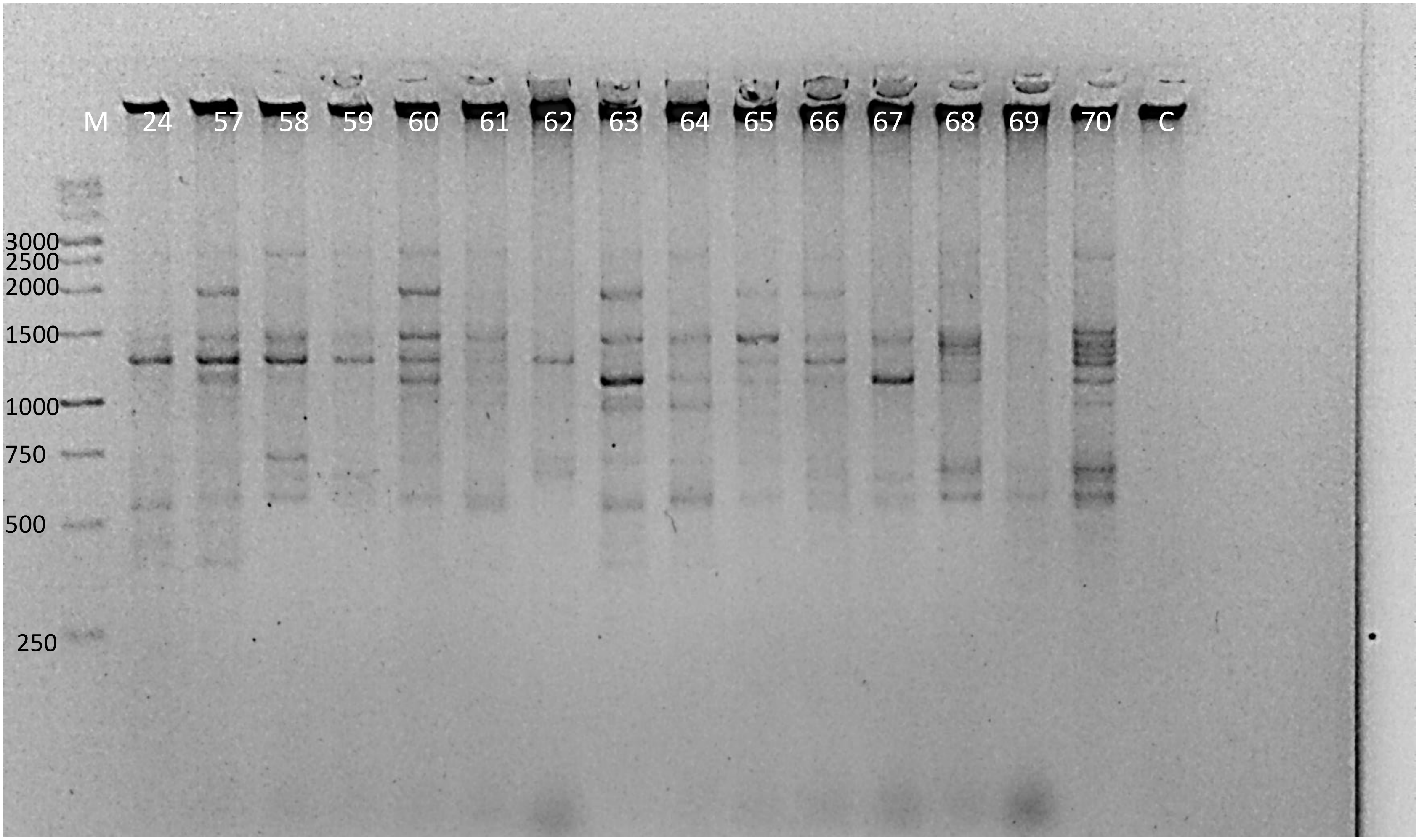
M
24
57
58
59
60
61
62
63
64
65
66
67
68
69
70
C
3000
2500
2000
1500
1000
750
500
250

ISSR814.1

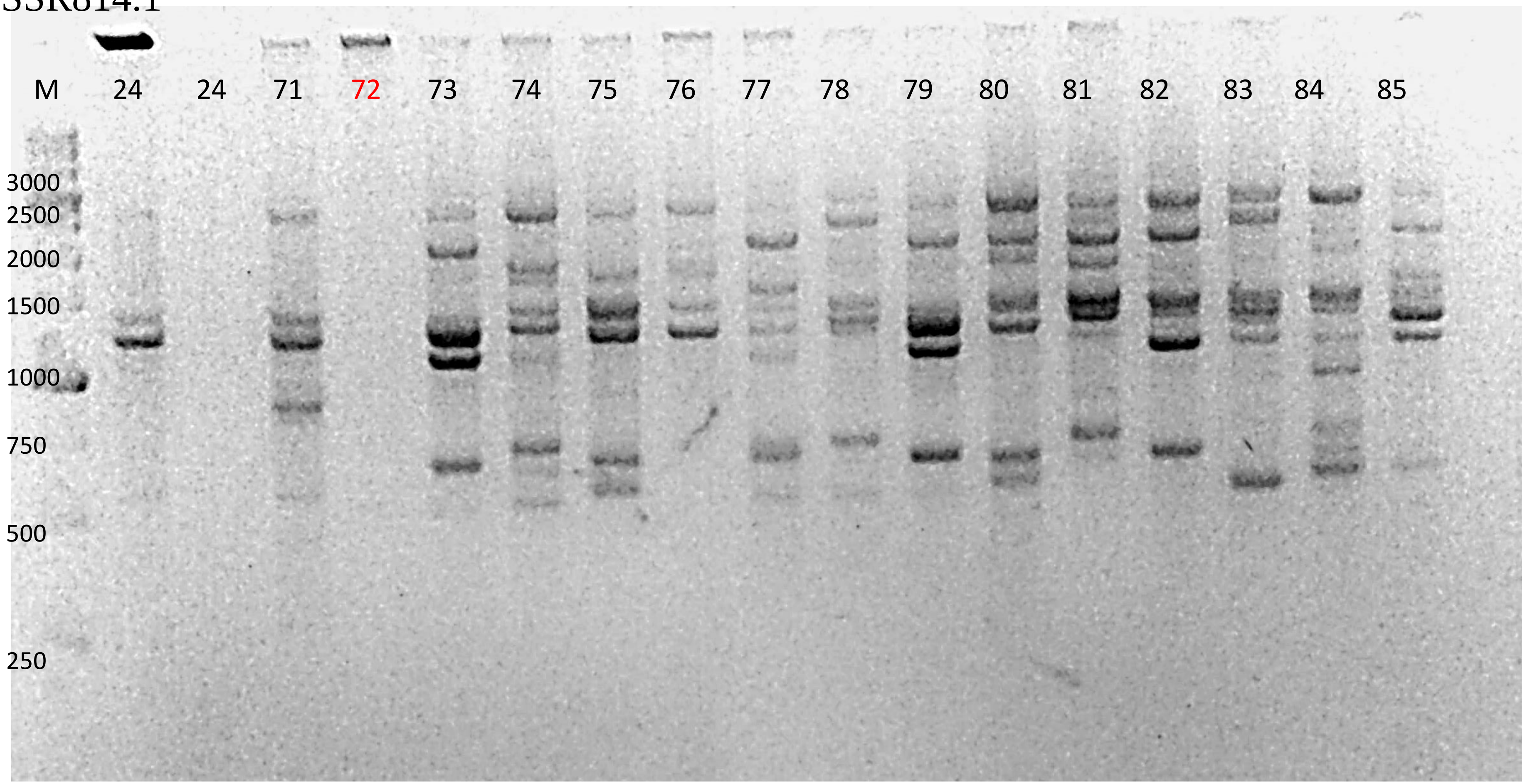
M
24
24
71
72
73
74
75
76
77
78
79
80
81
82
83
84
85
3000
2500
2000
1500
1000
750
500
250

ISSR814.1

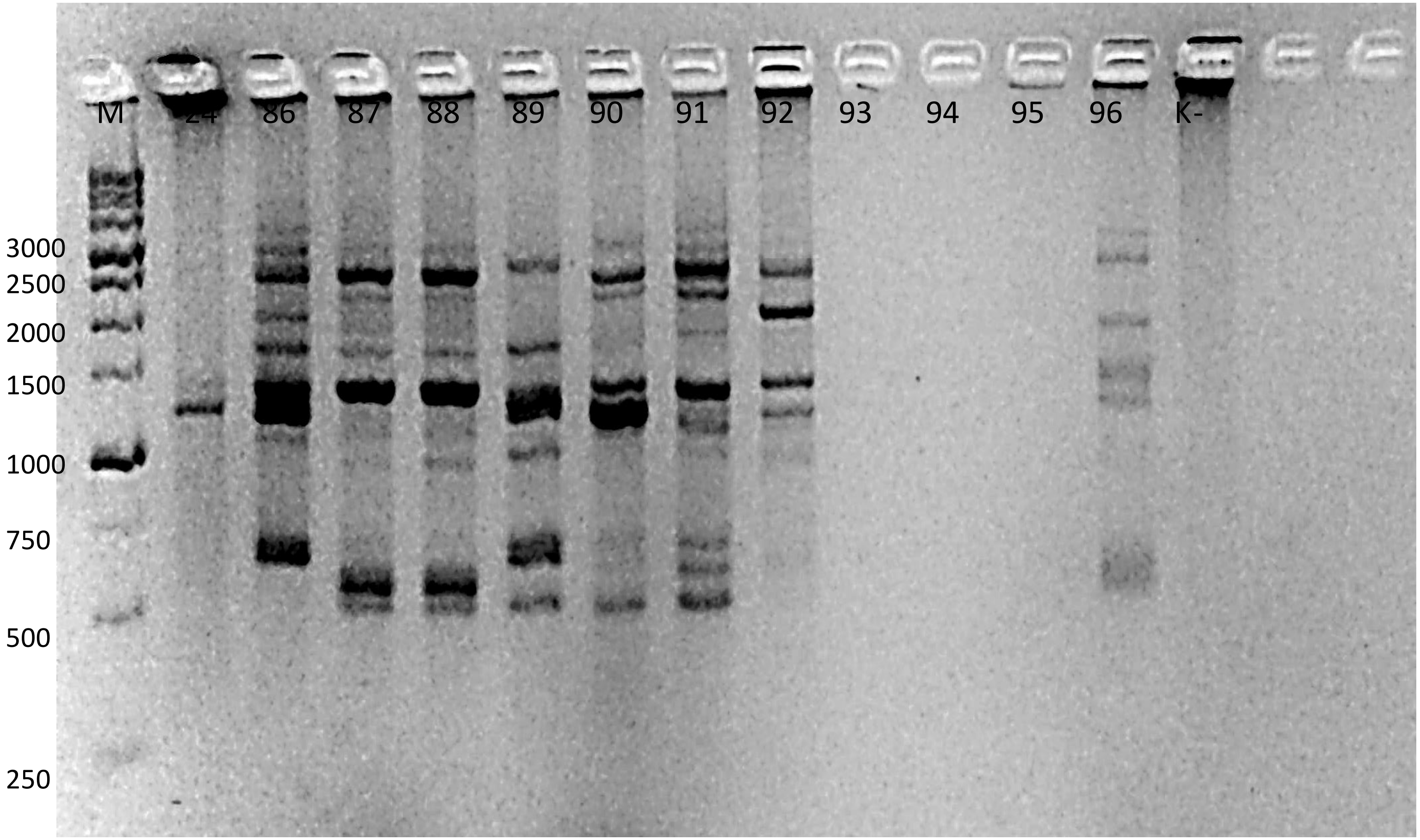
M
24
86
87
88
89
90
91
92
93
94
95
96
K-
3000
2500
2000
1500
1000
750
500
250

SSR gi298295865

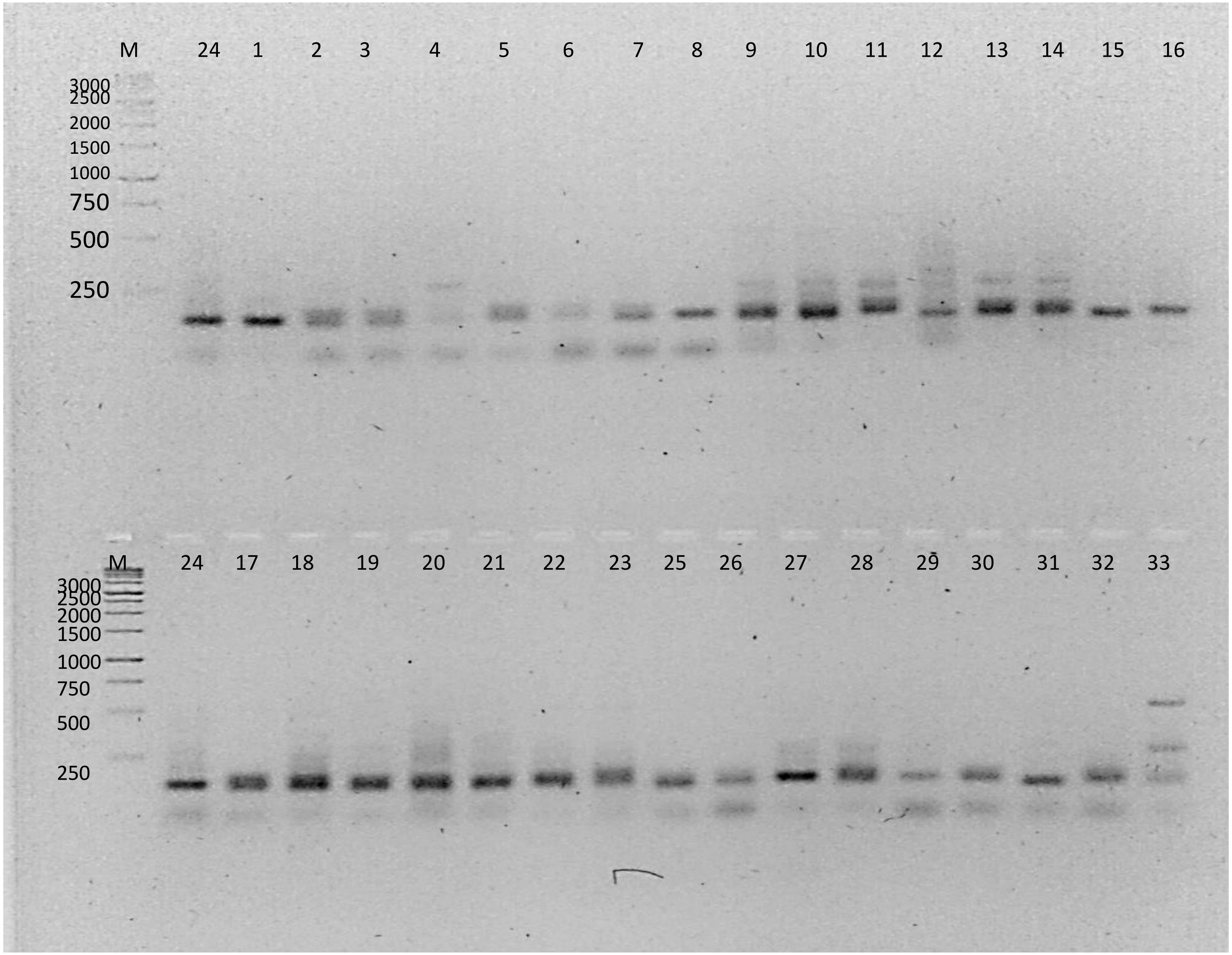

SSR gi298295865

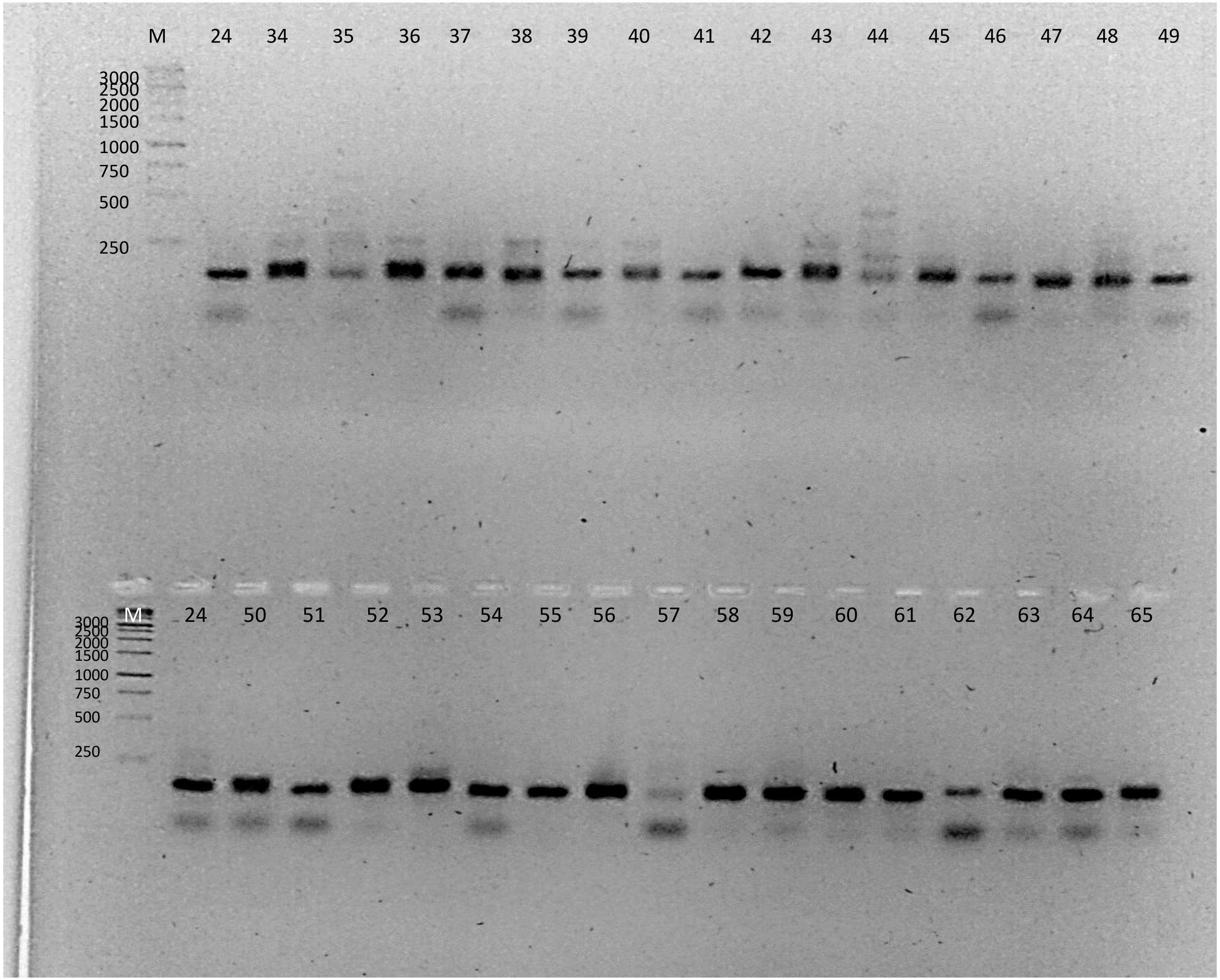

SSR gi298295865

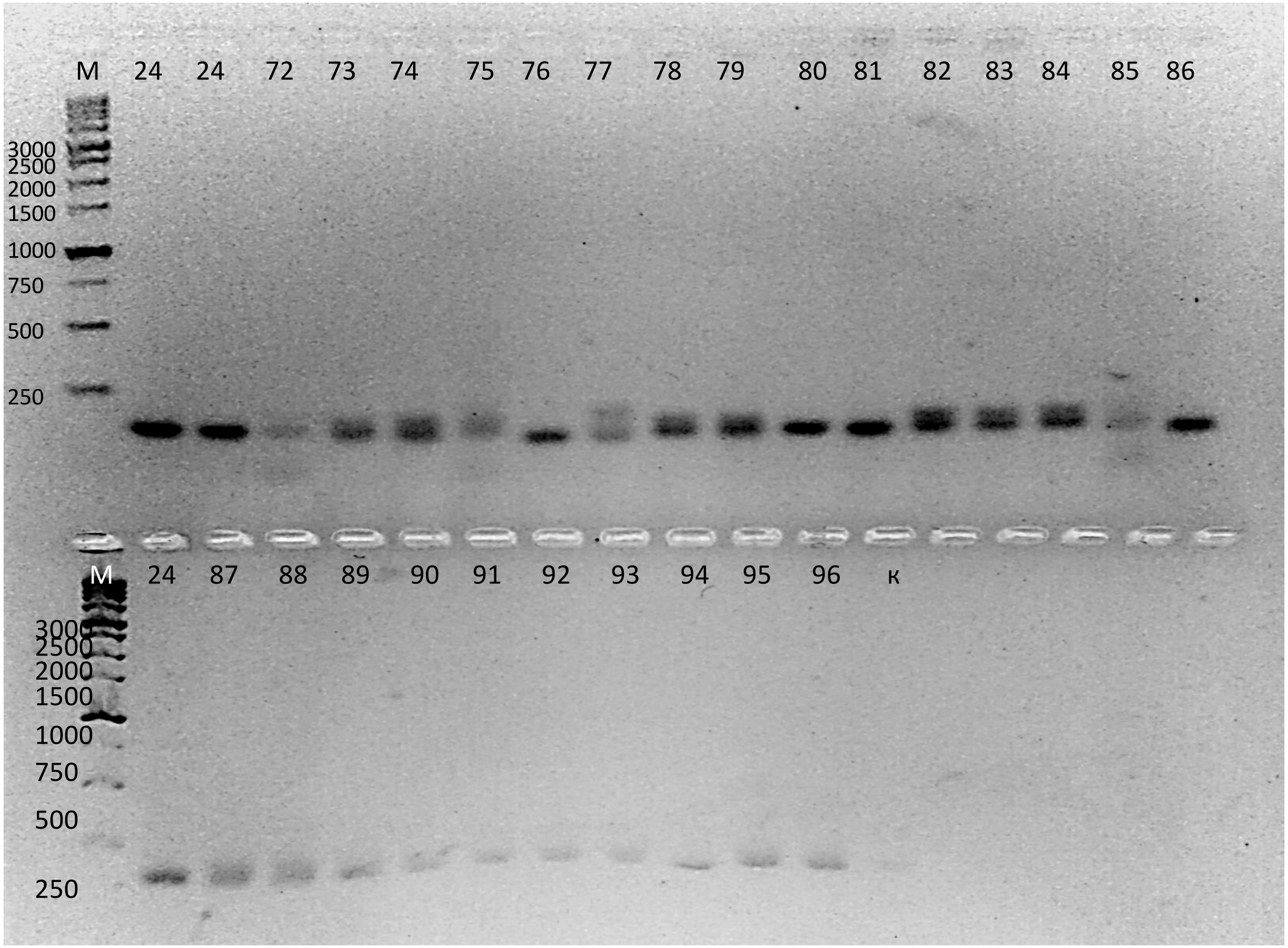

SSR gi298297301

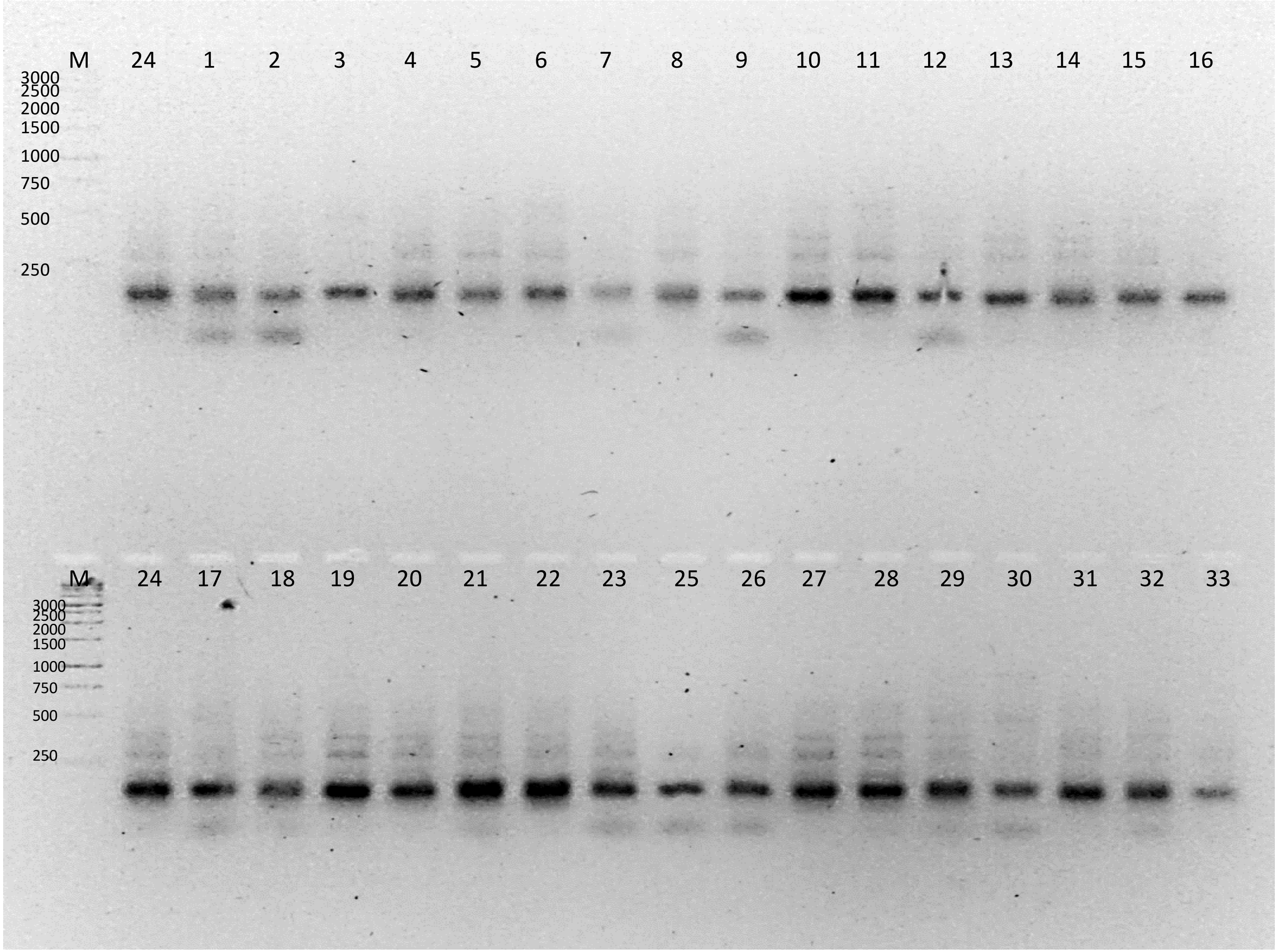

SSR gi298297301

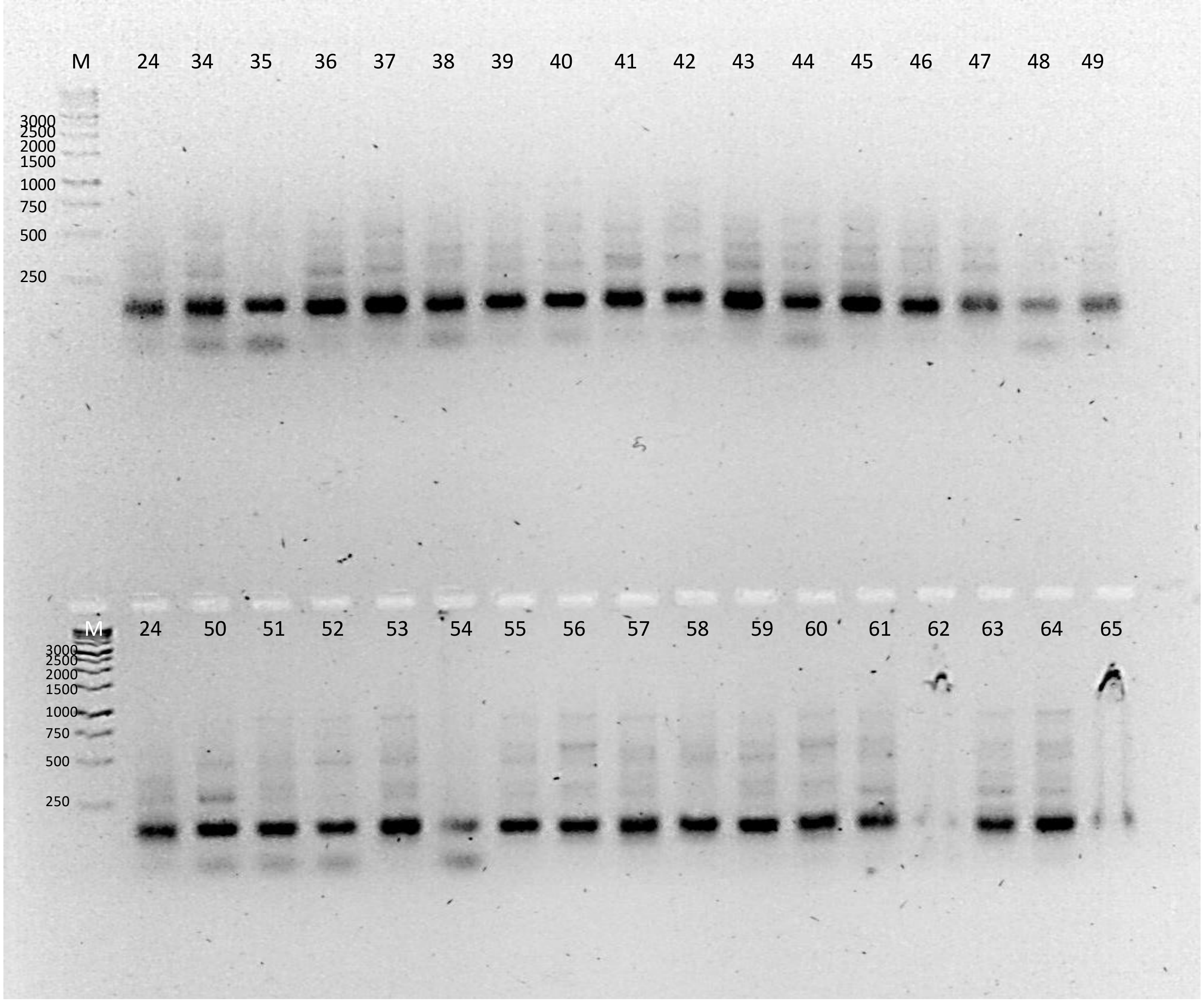

SSR gi298297301

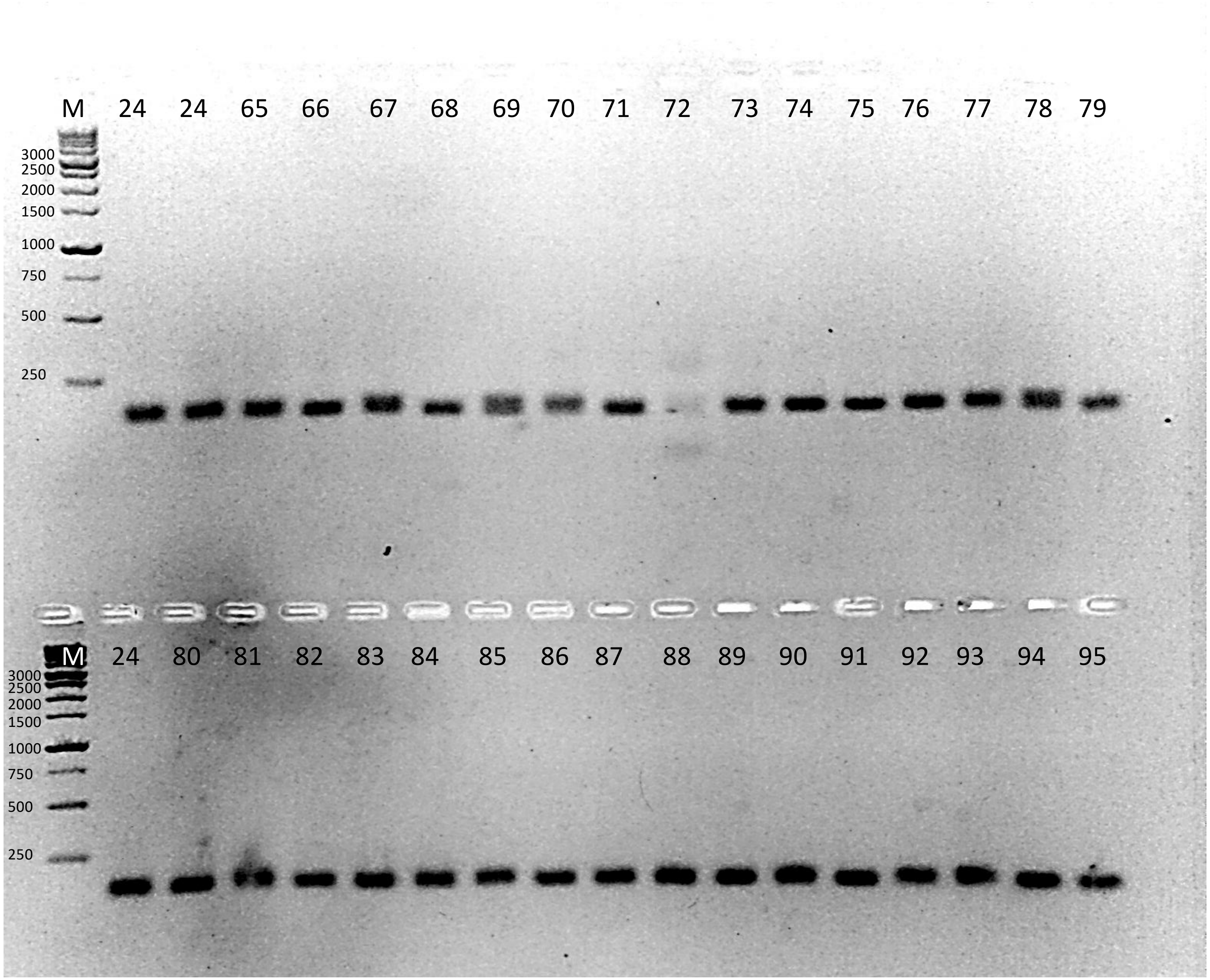

SSR320

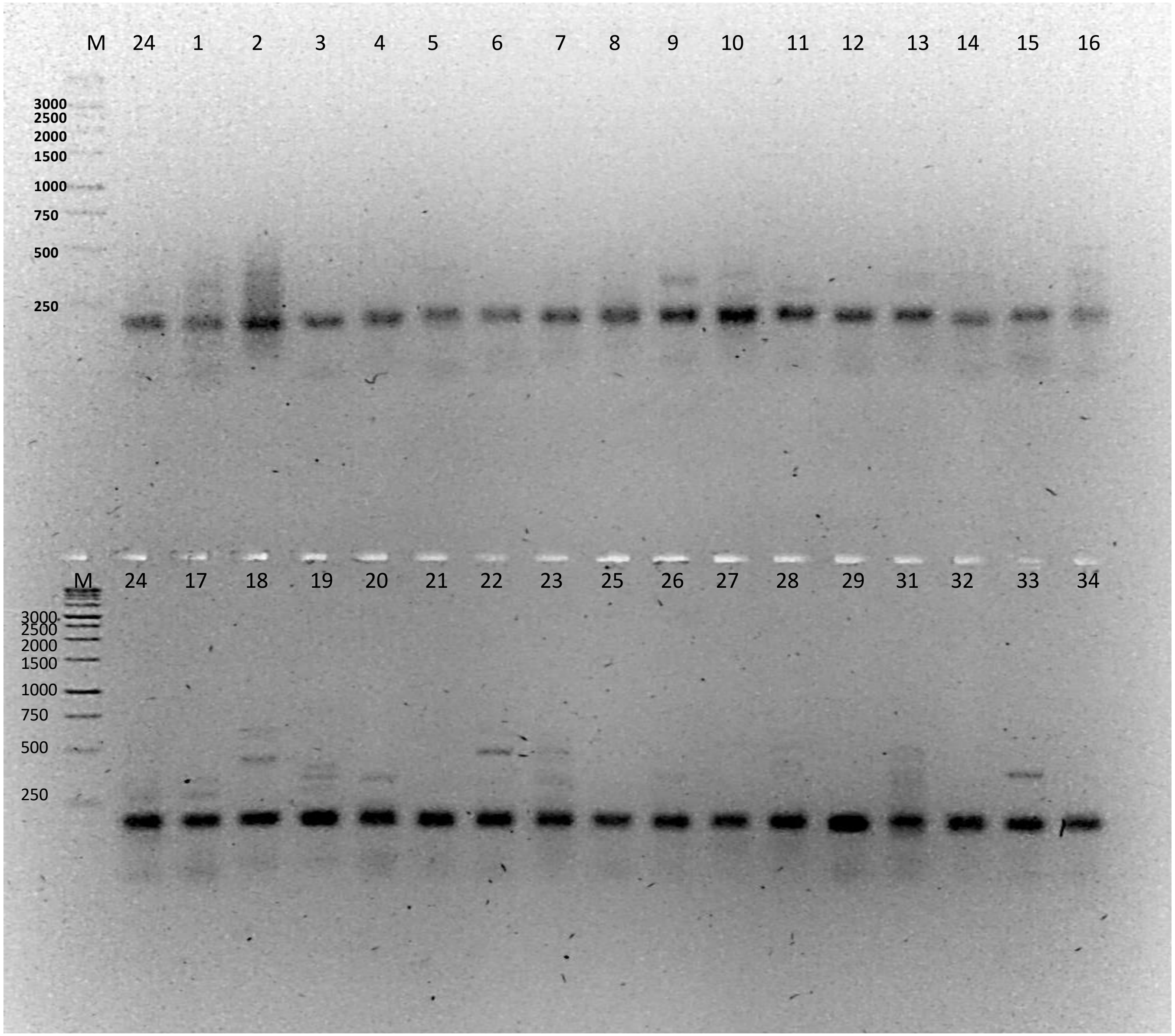
M 24 1 2 3 4 5 6 7 8 9 10 11 12 13 14 15 16
3000
2500
2000
1500
1000
750
500
250
M 24 17 18 19 20 21 22 23 25 26 27 28 29 31 32 33 34
3000
2500
2000
1500
1000
750
500
250

SSR320

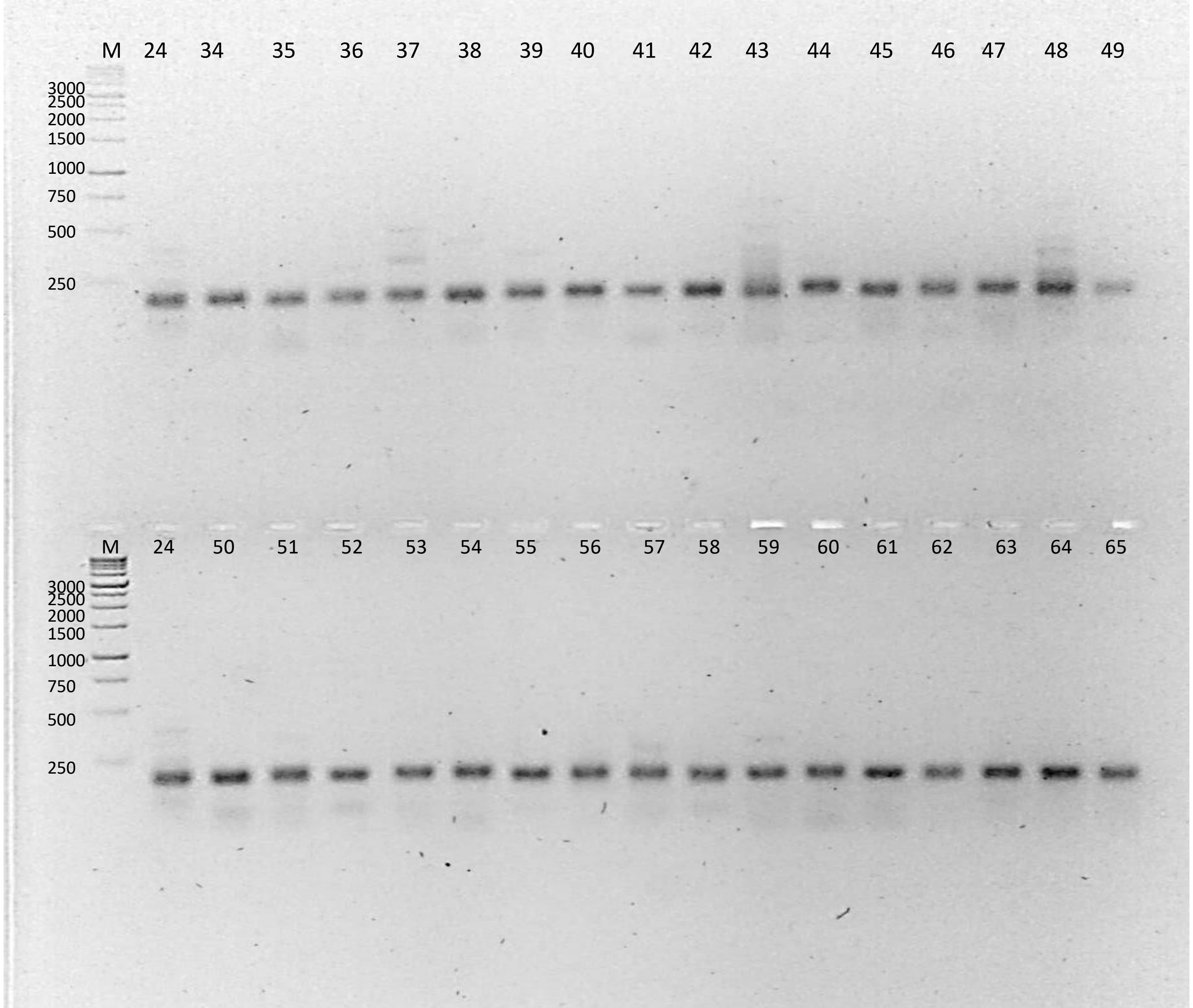
M 24 34 35 36 37 38 39 40 41 42 43 44 45 46 47 48 49
3000
2500
2000
1500
1000
750
500
250
M 24 50 51 52 53 54 55 56 57 58 59 60 61 62 63 64 65
3000
2500
2000
1500
1000
750
500
250

SSRgi298296818

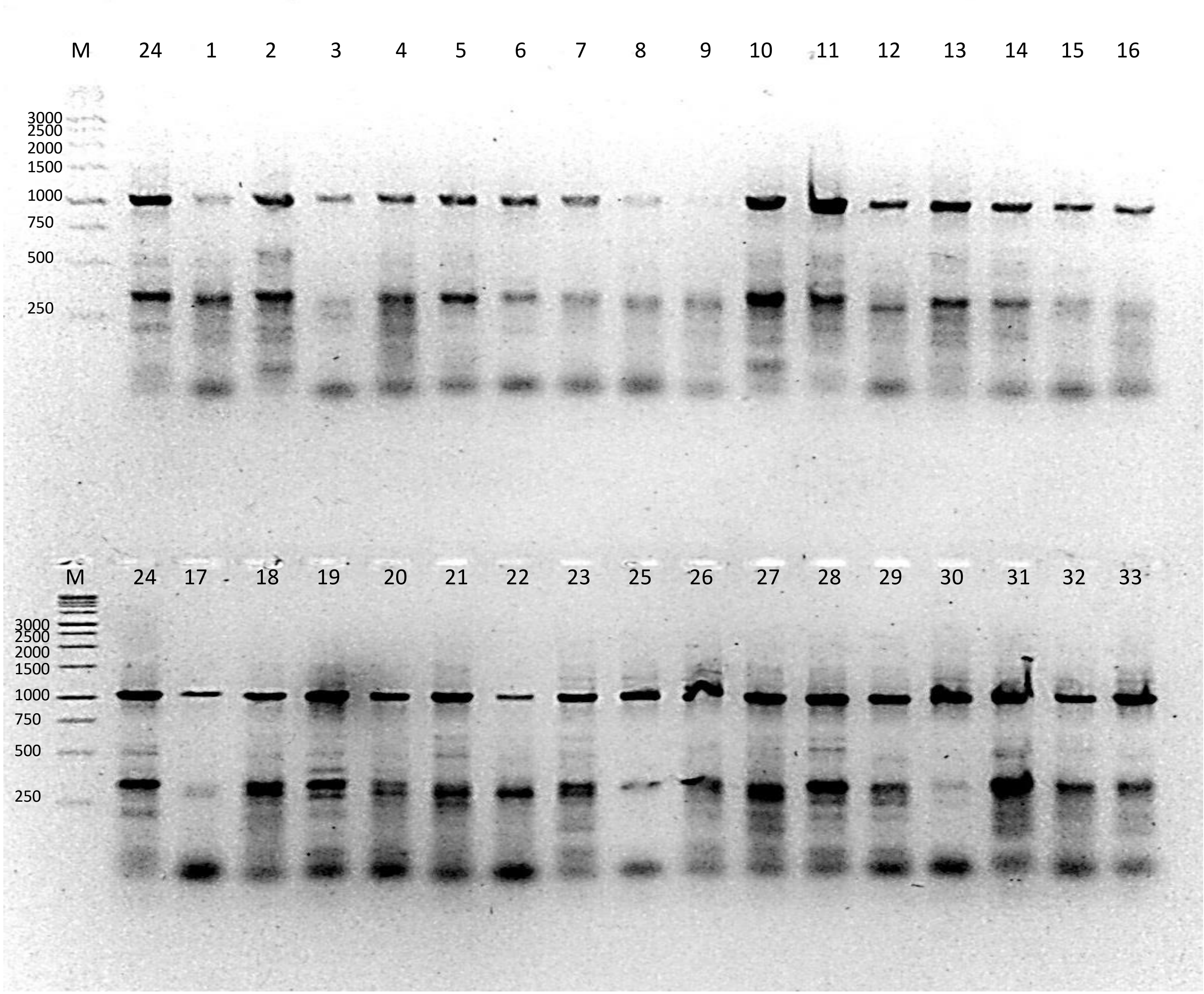

SSRgi298296818

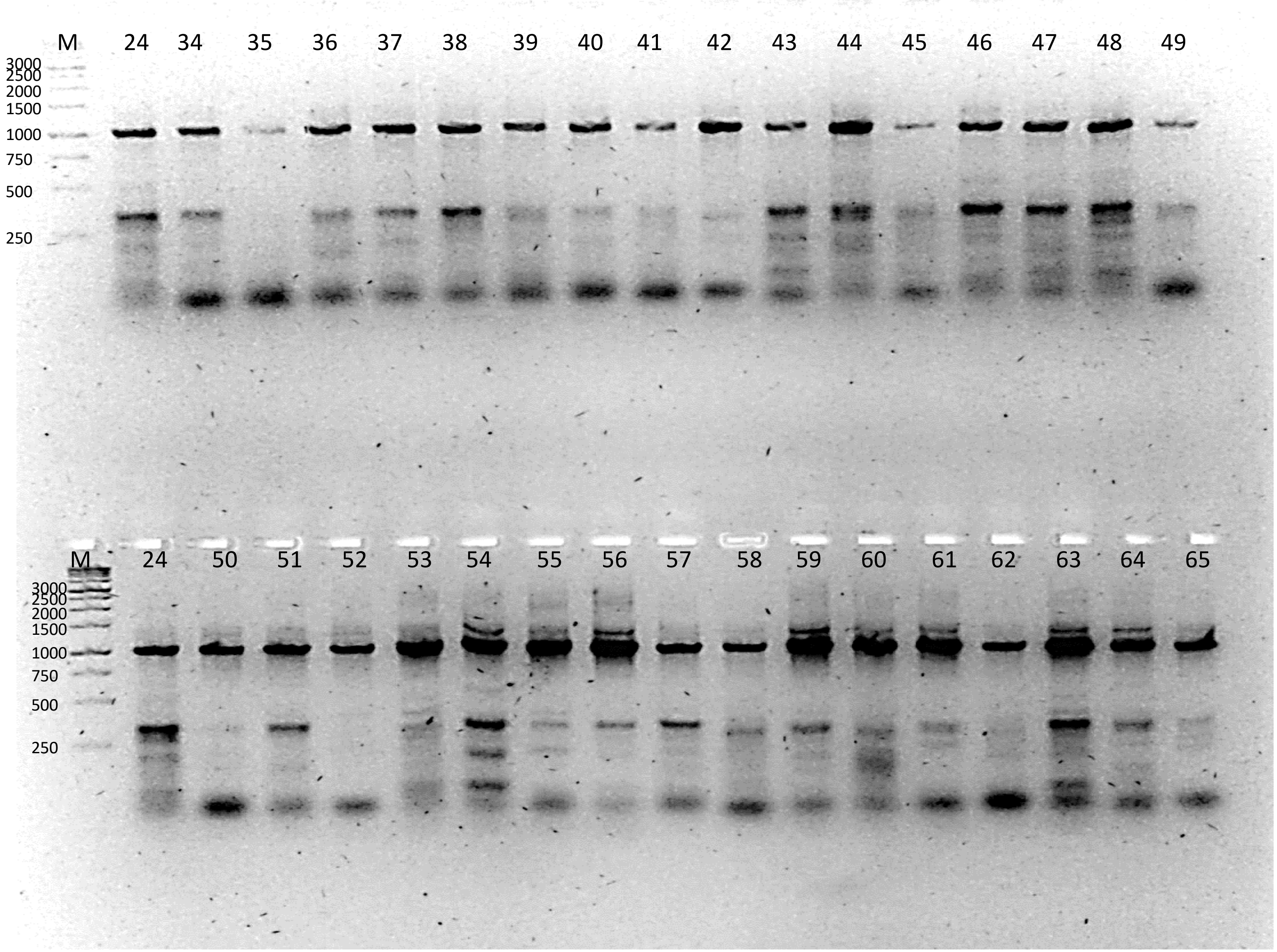

SSRgi298296818

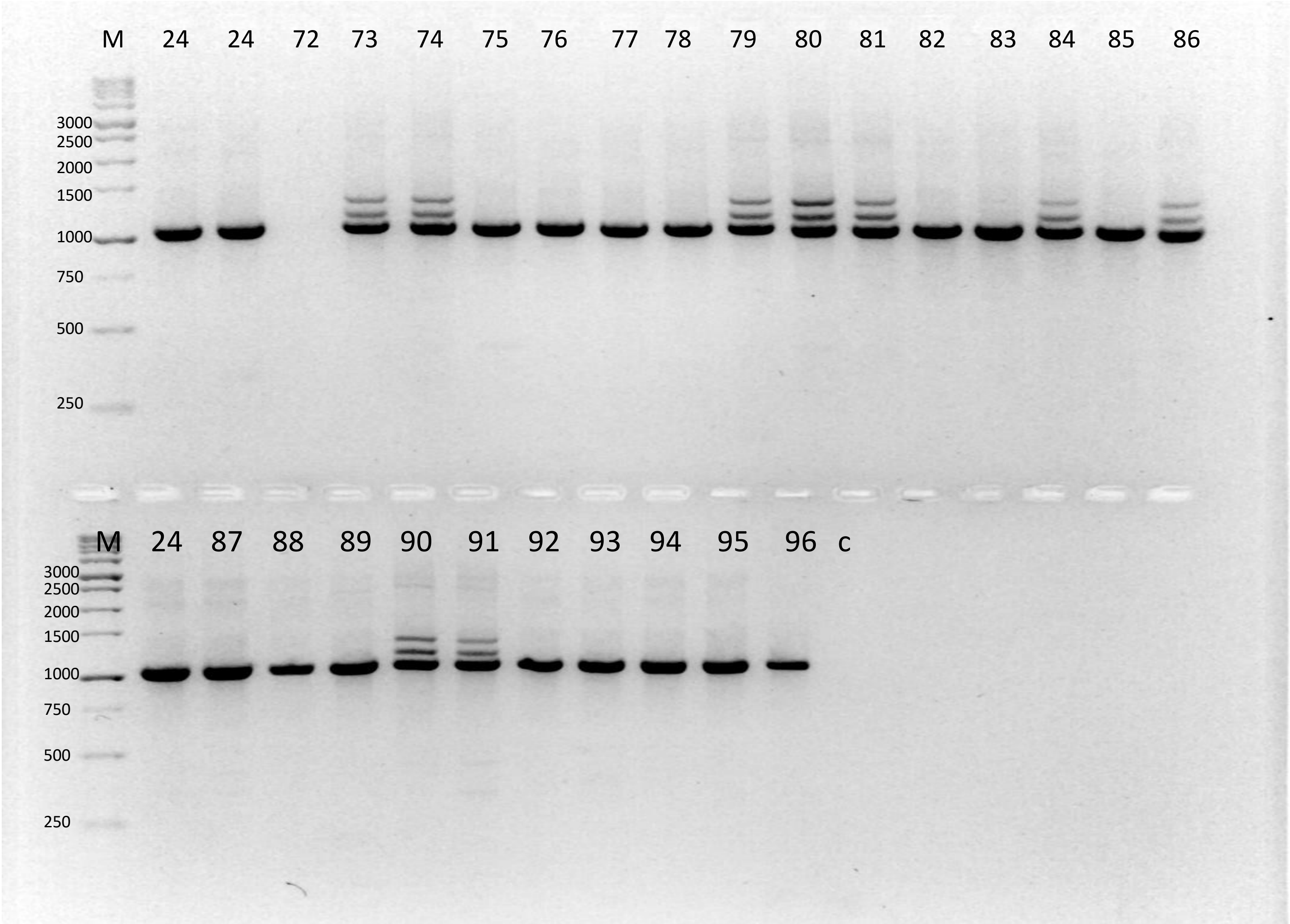

SSR357

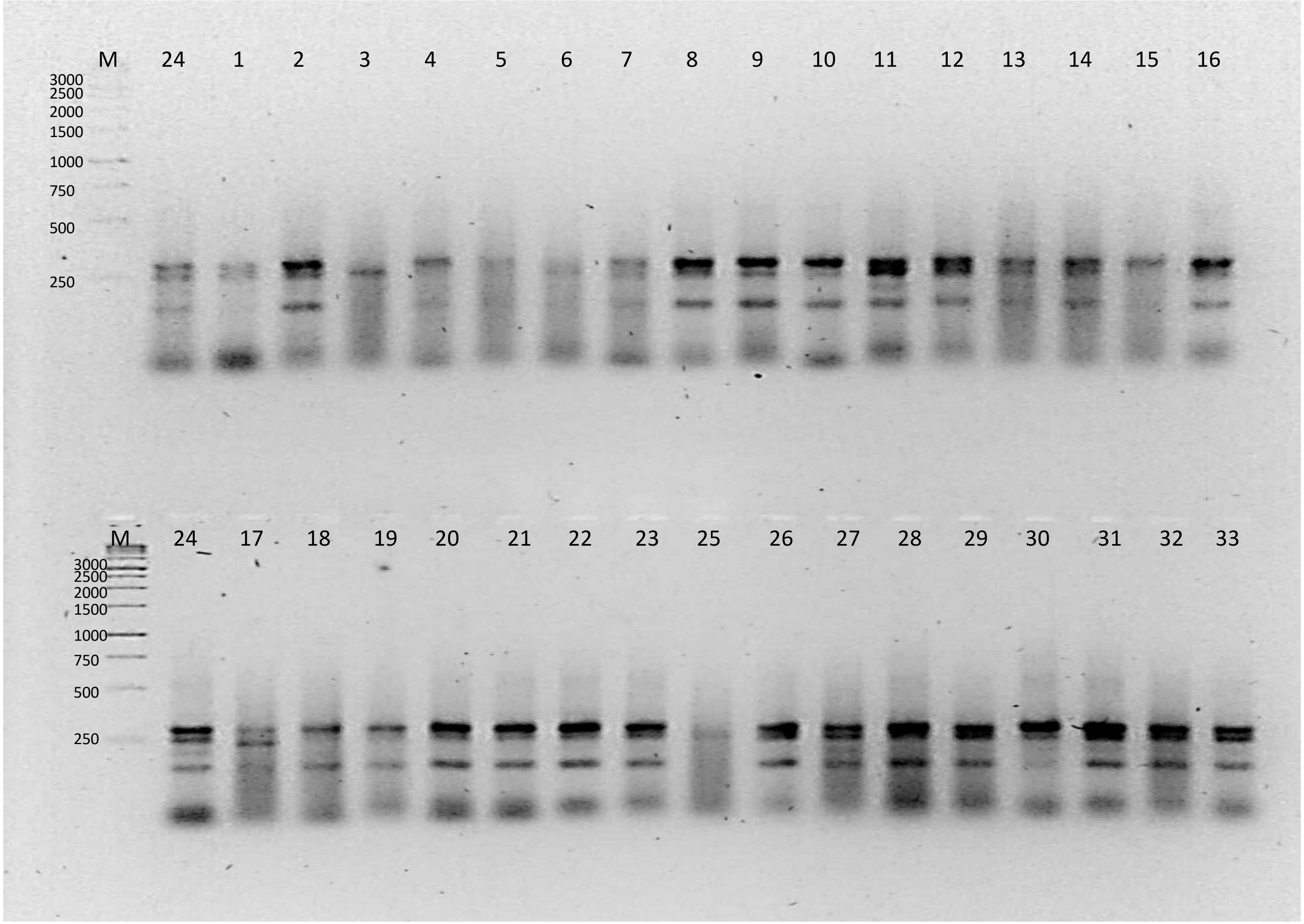
M 24 1 2 3 4 5 6 7 8 9 10 11 12 13 14 15 16
3000
2500
2000
1500
1000
750
500
250
M 24 17 18 19 20 21 22 23 25 26 27 28 29 30 31 32 33
3000
2500
2000
1500
1000
750
500
250

SSR357

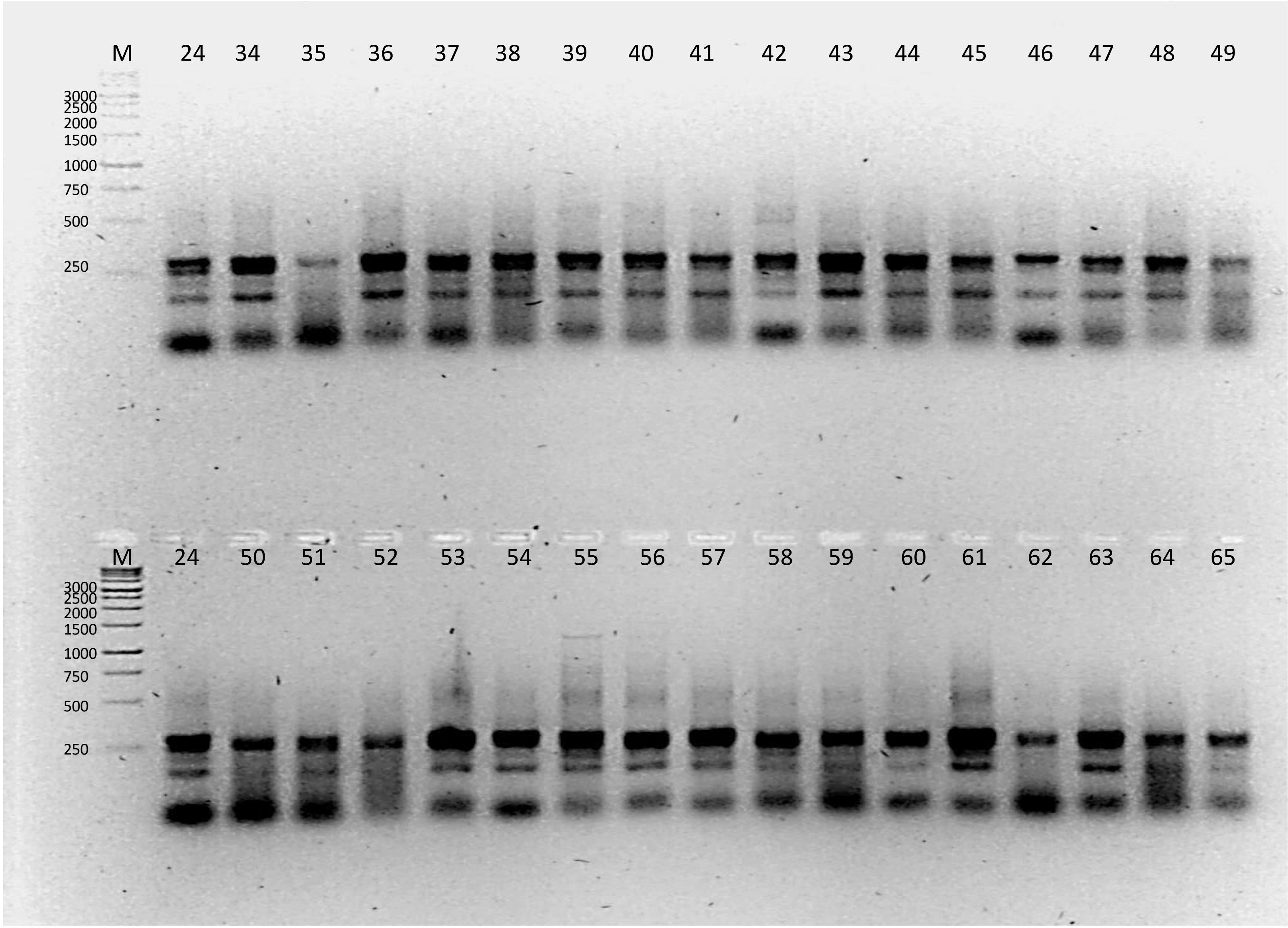
M 24 34 35 36 37 38 39 40 41 42 43 44 45 46 47 48 49
3000
2500
2000
1500
1000
750
500
250
M 24 50 51 52 53 54 55 56 57 58 59 60 61 62 63 64 65
3000
2500
2000
1500
1000
750
500
250

SSR357

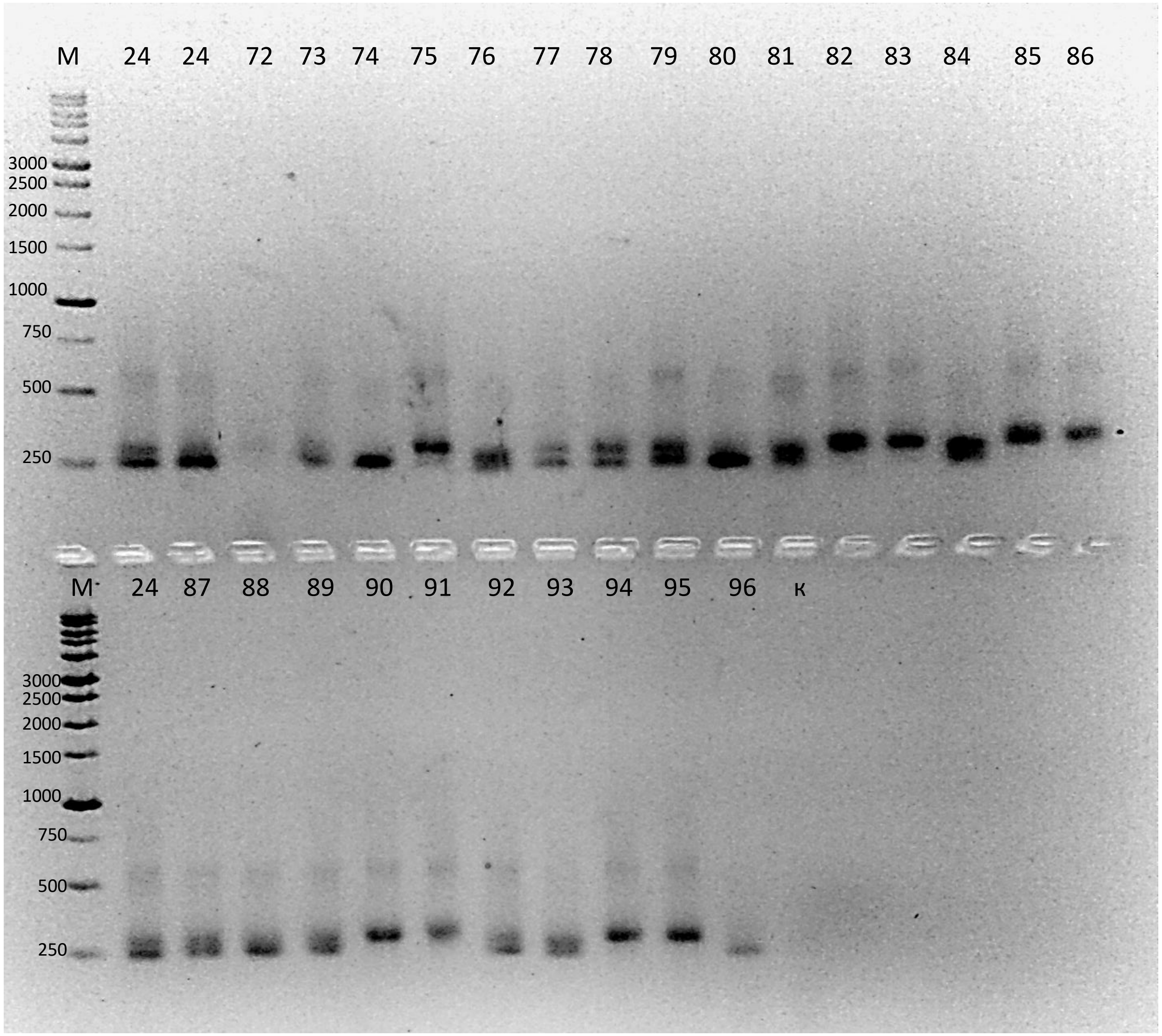
M 24 24 72 73 74 75 76 77 78 79 80 81 82 83 84 85 86
3000
2500
2000
1500
1000
750
500
250
M 24 87 88 89 90 91 92 93 94 95 96 к
3000
2500
2000
1500
1000
750
500
250
