# Supplementary material for: Transferability of ISSR, SCoT and SSR Markers for Chrysanthemum × Morifolium Ramat and Genetic Relationships Among Commercial Russian Cultivars
Source: Plants (Basel). 2021 Jun 27;10(7):1302. doi: 10.3390/plants10071302 (PMC8309030; doi:10.3390/plants10071302)
Supplement: Supplementary file 1 [file plants-10-01302-s001.zip › Suppl fig. Typical inflorescences.pdf]

Typical members of the each branch (fig 3A)

Branch I

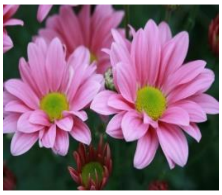

‘Grand Pink’

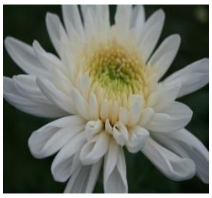

‘Wilhelmina’

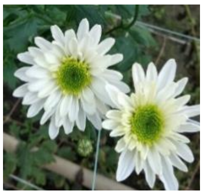

‘Mona Lisa’

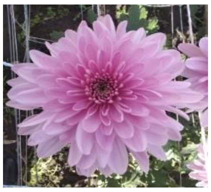

‘Ksenia’

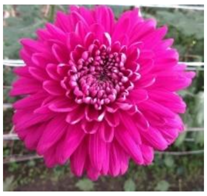

‘Desna’

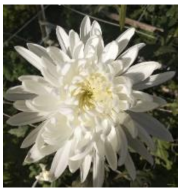

‘Gagarin’

Branch II

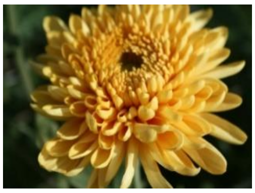

‘Princess Armgard Bronze’

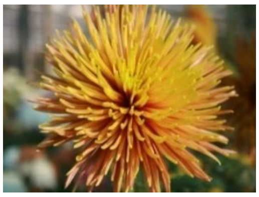

‘Saffina’

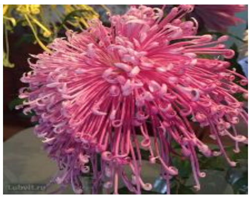

‘Spider Pink’

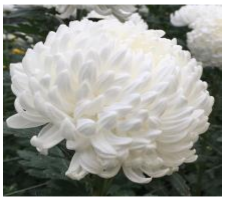

‘Rebonnet’

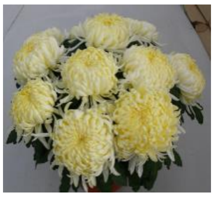

‘Angelys Jaune’

Branch IIIa

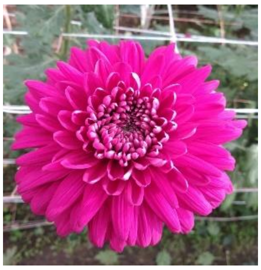

‘Desna’

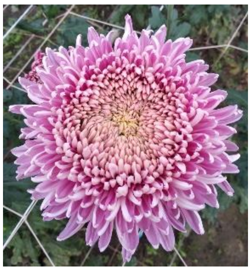

‘Cassandra pink’

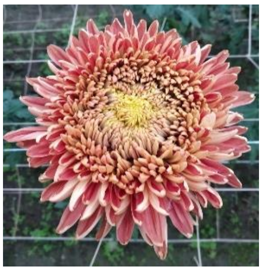

‘Cassandra bronze’

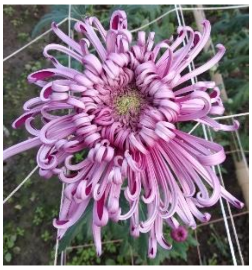

‘Saratov’

Branch IIIb

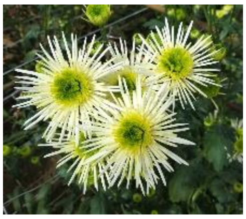
‘Vesuvio’

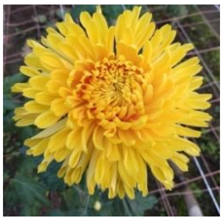
‘Izetka’

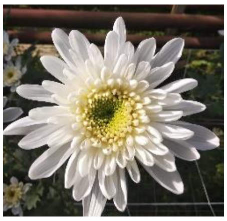
‘Baltica white’

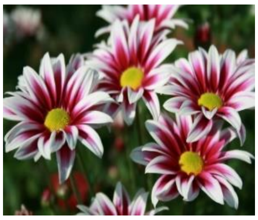
‘Harlequin’

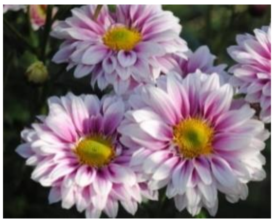
‘Nocturne’

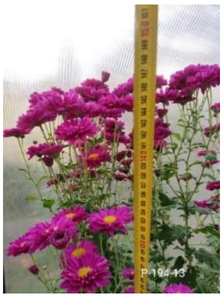
P-194-13

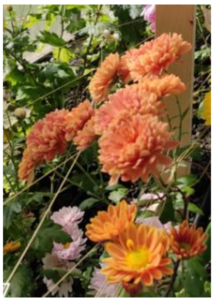
P-195-7

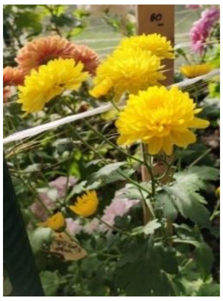
P-195-8

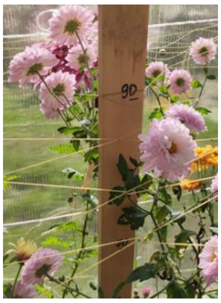
P-195-9

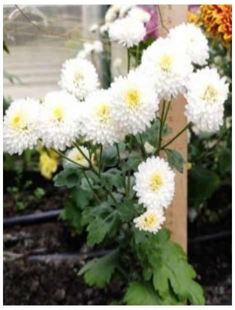
P-194-12

Branch IIIc1

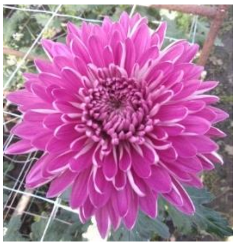
‘Sevan’

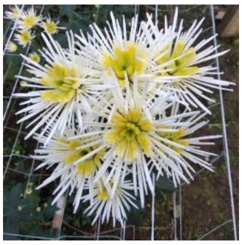
‘Annecy white’

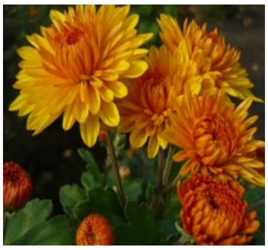
‘Zolotaya Osen``

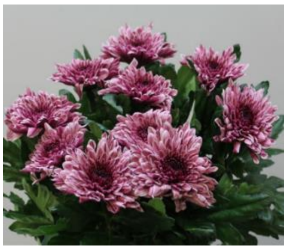
‘PIP Salmon’

BRANCH IIIc2

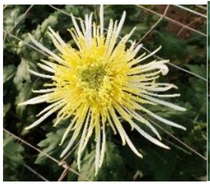
‘ Tokyo’

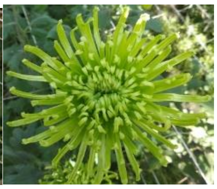
‘Anastasia green’

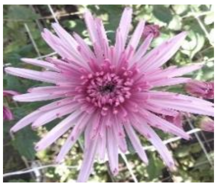
‘Anastasia pink’

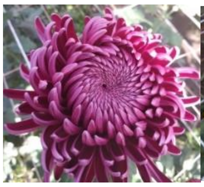
‘Bigoudi purple’

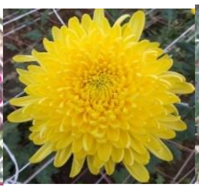
‘Ariana’

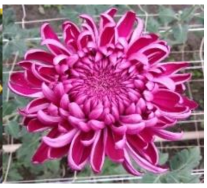
‘Gilbert’

Branch IIIc3.1

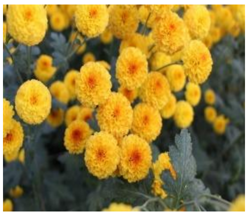
‘Focus’

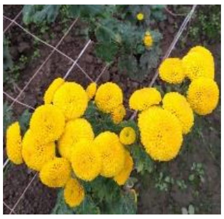
‘Statesman’

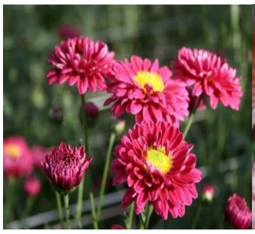
‘Goryanka’

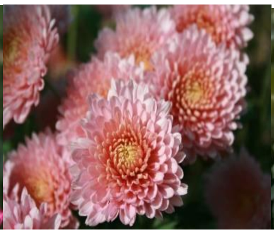
‘Rozovaya Dragocennost``’

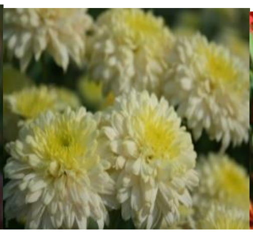
Nejnost’

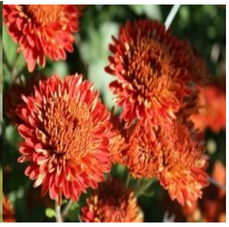
‘Krasnoe Znamya’

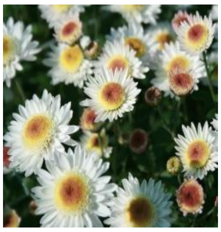
‘Medeya’

Branch IIIc3.2

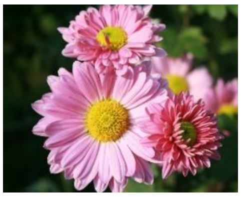
‘Sadko’

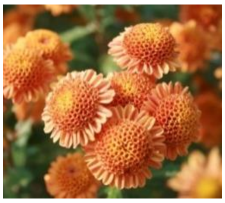
‘Nikolina’

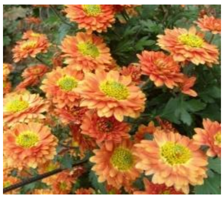
‘Nikitskaya Yubilejna’

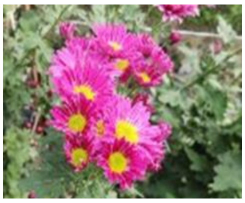
H-103-7

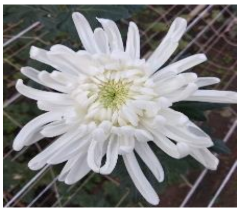
‘Regina’

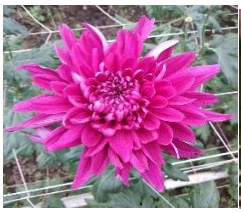
‘Rezume’

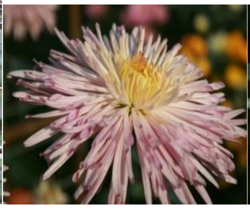
‘Mirazh’

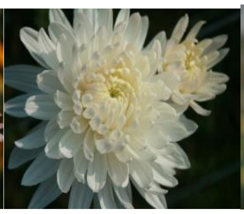
‘Princess Anna’

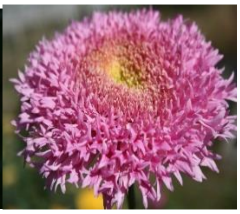
‘Etrusko’

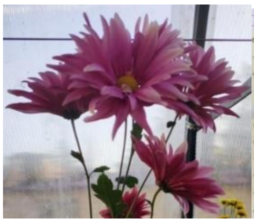
P-196-15

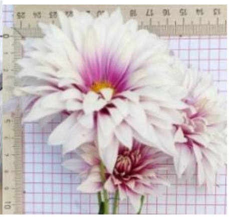
P-196-26

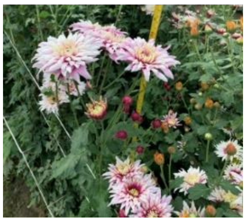
P-192-12
